# Supplementary material for: Polyketide synthase gene clusters in the ericoid mycorrhizal fungus Oidiodendron maius: genome-wide prediction, expression profiling, and functional characterization of knockout mutants
Source: Front Plant Sci. 2026 Sep 16;17:1924430. doi: 10.3389/fpls.2026.1924430 (PMC13623903; doi:10.3389/fpls.2026.1924430)
Supplement: Supplementary file 1 [file SupplementaryFile1.zip › Supplementary Materials/Supplementary Folder 1.zip!/Oidma1_AssemblyScaffolds_Repeatmasked/knownclusterblast/cluster24/jgi.p_Oidma1_165990_mibig_hits.html]

| MIBiG Protein | Description | MIBiG Cluster | MiBiG Product | % ID | % Coverage | BLAST Score | E-value |
| --- | --- | --- | --- | --- | --- | --- | --- |
| AGC45624.1 | polyketide\_synthase | BGC0001394 | Phenalamide\_biosynthetic\_gene\_cluster | 29 | 102.665482008 | 855 | 1.2e-247 |
| CCE88378.1 | polyketide\_synthase | BGC0001034 | Pellasoren\_biosynthetic\_gene\_cluster | 29 | 102.532207908 | 754 | 3.9e-217 |
| EWG54266.1 | hypothetical\_protein | BGC0001190 | Fusaric\_acid\_biosynthetic\_gene\_cluster | 36 | 57.0857396713 | 750 | 5.6e-216 |
| AAB08104.3 | polyketide\_synthase | BGC0000155\_c3 | T-toxin\_biosynthetic\_gene\_cluster | 35 | 58.7738782763 | 739 | 9.9e-213 |
| EHA28244.1 | hypothetical\_protein | BGC0001143 | Azanigerone\_biosynthetic\_gene\_cluster | 36 | 54.5091070635 | 726 | 1.1e-208 |
| BAV32159.1 | polyketide\_synthase | BGC0001373 | Sordarin\_biosynthetic\_gene\_cluster | 37 | 54.4202576633 | 724 | 4.3e-208 |
| BAN19720.1 | polyketide\_synthase | BGC0001252 | ACR-toxin\_biosynthetic\_gene\_cluster | 35 | 58.7738782763 | 708 | 3.2e-203 |
| BAC20566.1 | polyketide\_synthase | BGC0000039 | Compactin\_biosynthetic\_gene\_cluster | 33 | 65.7485561972 | 702 | 1.8e-201 |
| KGO40478.1 | Acyl\_transferase/acyl\_hydrolase/lysophospholipase | BGC0001205 | Communesin\_biosynthetic\_gene\_cluster | 32 | 65.4375832963 | 693 | 6.2e-199 |
| EHA19289.1 | hypothetical\_protein | BGC0001124 | Pyranonigrin\_E\_biosynthetic\_gene\_cluster | 35 | 57.6188360729 | 691 | 4e-198 |
| AHV78252.1 | ResS1 | BGC0001246 | Trans-resorcylide\_biosynthetic\_gene\_cluster | 35 | 57.1301643714 | 686 | 7.6e-197 |
| CAP95405.1 |  | BGC0001404 | Sorbicillin\_biosynthetic\_gene\_cluster | 35 | 54.997778765 | 683 | 6.5e-196 |
| ABA02240.1 | polyketide\_synthase | BGC0000098 | Monacolin\_K\_biosynthetic\_gene\_cluster | 35 | 52.332296757 | 673 | 8.7e-193 |
| CBF87072.1 | polyketide\_synthase,\_putative\_(Eurofung) | BGC0001290 | Emericellamide\_A\_/\_emericellamide\_B\_biosynthetic\_gene\_cluster | 37 | 53.7094624611 | 668 | 2.8e-191 |
| ABB76806.1 | polyketide\_synthase\_2 | BGC0000155\_c2 | T-toxin\_biosynthetic\_gene\_cluster | 35 | 55.7085739671 | 668 | 3.7e-191 |
| ACD39767.1 | reducing\_polyketide\_synthase | BGC0000077 | Hypothemycin\_biosynthetic\_gene\_cluster | 35 | 57.1301643714 | 666 | 1.4e-190 |
| AAD34559.1 | polyketide\_synthase | BGC0000089 | Lovastatin\_biosynthetic\_gene\_cluster | 35 | 53.2652154598 | 666 | 1.4e-190 |
| ACD39758.1 | reducing\_polyketide\_synthase | BGC0000076 | Hypothemycin\_biosynthetic\_gene\_cluster | 35 | 57.1301643714 | 664 | 4.1e-190 |
| AGC95324.1 | CurS1 | BGC0000045 | Dehydrocurvularin\_biosynthetic\_gene\_cluster | 35 | 54.2425588627 | 663 | 9e-190 |
| AHV78245.1 | LasS1 | BGC0001245 | Lasiodiplodin\_biosynthetic\_gene\_cluster | 34 | 57.5299866726 | 659 | 1.7e-188 |
| ACD39774.1 | reducing\_polyketide\_synthase | BGC0000134 | Radicicol\_biosynthetic\_gene\_cluster | 33 | 59.3513993781 | 648 | 3.9e-185 |
| ABB90283.1 | polyketide\_synthase | BGC0001057 | Zearalenone\_biosynthetic\_gene\_cluster | 35 | 53.665037761 | 646 | 8.8e-185 |
| BAD97694.1 | Aft9-1 | BGC0000003 | AF-toxin\_biosynthetic\_gene\_cluster | 34 | 53.7538871613 | 619 | 2e-176 |
| AKL78824.1 | GLPKS3 | BGC0001187 | Xenolozoyenone\_biosynthetic\_gene\_cluster | 36 | 54.2425588627 | 612 | 2.4e-174 |
| ACZ57548.1 | polyketide\_synthase | BGC0000046 | Depudecin\_biosynthetic\_gene\_cluster | 33 | 53.5317636606 | 596 | 1.8e-169 |
| ACS68554.1 | hybrid\_PKS-NRPS\_protein | BGC0001026 | NG-391\_biosynthetic\_gene\_cluster | 34 | 56.6859173701 | 592 | 2e-168 |
| AAT28740.1 | FUSS | BGC0000064 | Fusarin\_biosynthetic\_gene\_cluster | 33 | 56.2860950689 | 589 | 1.3e-167 |
| AFP73394.1 | FusA | BGC0001268 | Fusarin\_biosynthetic\_gene\_cluster | 33 | 56.4637938694 | 583 | 9.1e-166 |
| EAW09117.1 | hybrid\_NRPS/PKS\_enzyme,\_putative | BGC0000983 | Cytochalasin\_biosynthetic\_gene\_cluster | 32 | 60.5064415815 | 574 | 4.2e-163 |
| BAJ14522.1 | polyketide\_synthase | BGC0001253\_c4 | ACT-Toxin\_I\_biosynthetic\_gene\_cluster | 36 | 47.1790315415 | 573 | 1.2e-162 |
| EAU38971.1 | hypothetical\_protein | BGC0001122 | Isoflavipucine\_biosynthetic\_gene\_cluster | 36 | 47.3123056419 | 572 | 1.6e-162 |
| AIA58899.1 | HRPKS | BGC0001141 | Brefeldin\_biosynthetic\_gene\_cluster | 32 | 56.9080408707 | 569 | 1.8e-161 |
| ctg1\_orf0002 |  | BGC0001068 | Pyripyropene\_A\_biosynthetic\_gene\_cluster | 35 | 46.0239893381 | 559 | 1.8e-158 |
| AGO86662.1 | equisetin\_synthetase | BGC0001255 | Equisetin\_biosynthetic\_gene\_cluster | 32 | 56.6414926699 | 556 | 1.2e-157 |
| CBF80487.1 | hybrid\_PKS-NRPS\_(Eurofung) | BGC0000959 | Aspyridone\_biosynthetic\_gene\_cluster | 36 | 45.4908929365 | 552 | 1.7e-156 |
| ADN43685.1 | DmbS | BGC0001136 | Desmethylbassianin\_biosynthetic\_gene\_cluster | 33 | 58.5517547757 | 552 | 2.2e-156 |
| BAQ25466.1 | polyketide\_synthase | BGC0001264 | Betaenone\_A\_/\_betaenone\_B\_/\_betaenone\_C\_biosynthetic\_gene\_cluster | 35 | 46.6903598401 | 550 | 6.5e-156 |
| EAL89230.2 | LovB-like\_polyketide\_synthase,\_putative | BGC0000129 | Pyripyropene\_biosynthetic\_gene\_cluster | 35 | 46.5126610395 | 548 | 4.2e-155 |
| BAK26562.1 | PKS-NRPS\_hybrid | BGC0000977 | Cyclopiazonic\_acid\_biosynthetic\_gene\_cluster | 35 | 45.002221235 | 545 | 2.7e-154 |
| ABA02239.1 | polyketide\_synthase | BGC0000098 | Monacolin\_K\_biosynthetic\_gene\_cluster | 33 | 53.7538871613 | 544 | 6.1e-154 |
| AAV66110.2 | fusaridione\_A\_synthetase | BGC0000992 | Fusaridione\_A\_biosynthetic\_gene\_cluster | 36 | 45.5353176366 | 542 | 1.8e-153 |
| CBX87032.1 | BcBOA9\_protein | BGC0001243\_c3 | Botryenalol\_/\_botrydial\_/\_dihydrobotrydial\_/\_botryendial\_/ | 31 | 57.4411372723 | 542 | 3e-153 |
| AMY15068.1 | hexaketide\_synthase\_MF-SQHKS | BGC0001339 | Squalestatin\_S1\_biosynthetic\_gene\_cluster | 33 | 53.3984895602 | 539 | 2e-152 |
| CAO91861.1 | putative\_PKS-NRPS\_protein | BGC0000968 | Chaetoglobosin\_biosynthetic\_gene\_cluster | 33 | 54.331408263 | 534 | 4.8e-151 |
| CCT72377.1 | probable\_polyketide\_synthase | BGC0001305 | Fujikurins\_biosynthetic\_gene\_cluster | 34 | 47.3567303421 | 531 | 4.1e-150 |
| EAL85129.1 | polyketide\_synthase,\_putative | BGC0001067 | Fumagillin\_biosynthetic\_gene\_cluster | 31 | 56.7303420702 | 531 | 5.4e-150 |
| BAC20564.1 | polyketide\_synthase | BGC0000039 | Compactin\_biosynthetic\_gene\_cluster | 33 | 53.3096401599 | 531 | 5.4e-150 |
| EAL85113.2 | hybrid\_PKS-NRPS\_enzyme,\_putative | BGC0001037 | Pseurotin\_A\_biosynthetic\_gene\_cluster | 32 | 57.6188360729 | 528 | 3.5e-149 |
| AAF19810.1 | MtaB | BGC0001024 | Myxothiazol\_biosynthetic\_gene\_cluster | 32 | 52.3767214571 | 520 | 9.5e-147 |
| CCE88376.1 | polyketide\_synthase | BGC0001034 | Pellasoren\_biosynthetic\_gene\_cluster | 31 | 52.5099955575 | 511 | 4.4e-144 |
| BAG17643.1 | putative\_NRPS-type-I\_PKS\_fusion\_protein | BGC0001043 | SGR\_PTMs\_biosynthetic\_gene\_cluster | 31 | 57.7521101733 | 507 | 8.3e-143 |
| CAQ43076.1 | polyketide\_synthase | BGC0000970 | Chondrochloren\_biosynthetic\_gene\_cluster | 33 | 53.0875166593 | 507 | 1.1e-142 |
| EAQ86385.1 | hypothetical\_protein | BGC0001405 | Chaetoviridin\_/\_chaetomugilin\_biosynthetic\_gene\_cluster | 37 | 35.7618836073 | 504 | 7e-142 |
| AIT55263.1 | GulF | BGC0000072 | Gulmirecin\_biosynthetic\_gene\_cluster | 32 | 54.331408263 | 503 | 9.2e-142 |
| CBD77748.1 | polyketide\_synthase | BGC0000974 | Crocacin\_biosynthetic\_gene\_cluster | 32 | 53.4429142603 | 503 | 1.2e-141 |
| ACB46196.1 | polyketide\_synthase | BGC0000989 | Epothilone\_biosynthetic\_gene\_cluster | 32 | 52.7321190582 | 501 | 4.5e-141 |
| ADB12492.1 | EpoE | BGC0000990 | Epothilone\_biosynthetic\_gene\_cluster | 32 | 52.6432696579 | 501 | 5.9e-141 |
| AAF62884.1 | EpoE | BGC0000991 | Epothilone\_biosynthetic\_gene\_cluster | 32 | 52.6432696579 | 501 | 5.9e-141 |
| EFL02193.1 | amino\_acid\_adenylation\_domain-containing\_protein | BGC0000996 | Frontalamides\_biosynthetic\_gene\_cluster | 30 | 59.2625499778 | 500 | 1.3e-140 |
| ANI24099.1 | polyketide\_synthase | BGC0001235 | Antalid\_biosynthetic\_gene\_cluster | 32 | 53.0430919591 | 500 | 1.3e-140 |
| AAF26922.1 | polyketide\_synthase | BGC0000988 | Epothilone\_biosynthetic\_gene\_cluster | 32 | 52.7321190582 | 497 | 8.6e-140 |
| AMY15057.1 | tetraketide\_synthase\_MF-SQTKS | BGC0001339 | Squalestatin\_S1\_biosynthetic\_gene\_cluster | 34 | 43.1808085295 | 496 | 1.9e-139 |
| ABL86391.1 | hybrid\_polyketide\_synthase\_and\_nonribosomal\_peptide\_synthetase | BGC0000999 | Heat-stable\_antifungal\_factor\_biosynthetic\_gene\_cluster | 32 | 50.8218569525 | 495 | 3.3e-139 |
| CAQ43075.1 | polyketide\_synthase | BGC0000970 | Chondrochloren\_biosynthetic\_gene\_cluster | 32 | 52.8209684585 | 495 | 4.3e-139 |
| AAA79984.2 | soraphen\_polyketide\_synthase\_B | BGC0000147 | Soraphen\_biosynthetic\_gene\_cluster | 32 | 52.5099955575 | 494 | 7.3e-139 |
| ACR33078.1 | polyketide\_synthase | BGC0000017 | Anatoxin\_biosynthetic\_gene\_cluster | 31 | 51.3549533541 | 493 | 1.2e-138 |
| AIW82279.1 | PuwB | BGC0001125 | Puwainaphycins\_biosynthetic\_gene\_cluster | 32 | 52.4211461573 | 492 | 2.1e-138 |
| CAQ18830.1 | polyketide\_synthase | BGC0000954 | Ajudazol\_biosynthetic\_gene\_cluster | 31 | 52.9098178587 | 491 | 6.1e-138 |
| AEE88280.1 | CurJ | BGC0000976 | Curacin\_biosynthetic\_gene\_cluster | 30 | 53.2652154598 | 489 | 2.3e-137 |
| AAT70105.1 | CurJ | BGC0001165 | Curacin\_biosynthetic\_gene\_cluster | 30 | 53.2652154598 | 489 | 2.3e-137 |
| CAQ34920.1 | polyketide\_synthase | BGC0000986 | Dkxanthene\_biosynthetic\_gene\_cluster | 30 | 57.5299866726 | 489 | 2.3e-137 |
| AGC45622.1 | polyketide\_synthase | BGC0001394 | Phenalamide\_biosynthetic\_gene\_cluster | 31 | 52.2434473567 | 488 | 4e-137 |
| AGC45620.1 | polyketide\_synthase | BGC0001394 | Phenalamide\_biosynthetic\_gene\_cluster | 31 | 53.7538871613 | 488 | 5.2e-137 |
| CAG28678.1 | polyketide\_synthase | BGC0001023 | Myxochromide\_biosynthetic\_gene\_cluster | 33 | 44.1137272323 | 487 | 8.9e-137 |
| CAD19090.1 | StiF\_protein | BGC0000153 | Stigmatellin\_biosynthetic\_gene\_cluster | 31 | 52.3767214571 | 485 | 3.4e-136 |
| AEE88279.1 | CurK | BGC0000976 | Curacin\_biosynthetic\_gene\_cluster | 30 | 53.5761883607 | 483 | 9.8e-136 |
| AAT70106.1 | CurK | BGC0001165 | Curacin\_biosynthetic\_gene\_cluster | 30 | 53.5761883607 | 483 | 9.8e-136 |
| XP\_001220460.1 | hypothetical\_protein | BGC0001182 | Chaetoglobosins\_biosynthetic\_gene\_cluster | 34 | 50.7330075522 | 481 | 4.9e-135 |
| ALD82522.1 | polyketide\_synthase | BGC0001212 | Nannocystin\_biosynthetic\_gene\_cluster | 32 | 52.1545979565 | 480 | 1.4e-134 |
| CAL69597.1 | nonribosomal\_peptide\_synthase | BGC0001049 | Tenellin\_biosynthetic\_gene\_cluster | 36 | 40.4264771213 | 479 | 1.9e-134 |
| AAK57187.1 | MxaC | BGC0001022 | Myxalamid\_biosynthetic\_gene\_cluster | 31 | 53.7983118614 | 478 | 3.2e-134 |
| BAJ16467.1 | polyketide\_synthase | BGC0000058 | FD-891\_biosynthetic\_gene\_cluster | 31 | 52.7765437583 | 476 | 2e-133 |
| EAA65604.1 | hypothetical\_protein | BGC0000022 | Asperfuranone\_biosynthetic\_gene\_cluster | 37 | 34.9622390049 | 476 | 2e-133 |
| ADM46358.1 | polyketide\_synthase | BGC0000106 | Naphthomycin\_biosynthetic\_gene\_cluster | 32 | 52.8209684585 | 475 | 4.6e-133 |
| CAJ46690.1 | polyketide\_synthase | BGC0000969 | Chondramide\_biosynthetic\_gene\_cluster | 31 | 52.1545979565 | 475 | 4.6e-133 |
| AAK57189.1 | MxaE | BGC0001022 | Myxalamid\_biosynthetic\_gene\_cluster | 32 | 47.7565526433 | 474 | 7.8e-133 |
| CAL58681.1 | polyketide\_synthase | BGC0000149 | Spirangien\_biosynthetic\_gene\_cluster | 31 | 53.0430919591 | 473 | 1e-132 |
| AAK57188.1 | MxaD | BGC0001022 | Myxalamid\_biosynthetic\_gene\_cluster | 30 | 52.3767214571 | 471 | 6.6e-132 |
| BAF02922.1 | type\_I\_polyketide\_synthase | BGC0000073 | Halstoctacosanolide\_biosynthetic\_gene\_cluster | 30 | 53.1319413594 | 470 | 8.6e-132 |
| BAD83684.1 | PKSN\_polyketide\_synthase\_for\_alternapyrone\_biosynthesis | BGC0000012 | Alternapyrone\_biosynthetic\_gene\_cluster | 32 | 43.2696579298 | 468 | 3.3e-131 |
| AGC45621.1 | polyketide\_synthase | BGC0001394 | Phenalamide\_biosynthetic\_gene\_cluster | 30 | 52.5099955575 | 466 | 2.1e-130 |
| ADZ24998.1 | polyketide\_synthase | BGC0000380 | Leupyrrin\_biosynthetic\_gene\_cluster | 29 | 53.8427365615 | 465 | 2.8e-130 |
| AAK19883.1 | soraphen\_polyketide\_synthase\_A | BGC0000147 | Soraphen\_biosynthetic\_gene\_cluster | 33 | 44.0693025322 | 465 | 2.8e-130 |
| ADH04639.1 | TgaA | BGC0001051 | Thuggacin\_biosynthetic\_gene\_cluster | 31 | 52.7765437583 | 463 | 1.1e-129 |
| ADF88280.1 | polyketide\_synthase | BGC0000981 | Cylindrospermopsin\_biosynthetic\_gene\_cluster | 32 | 45.1799200355 | 463 | 1.8e-129 |
| CAQ34919.1 | polyketide\_synthase | BGC0000986 | Dkxanthene\_biosynthetic\_gene\_cluster | 30 | 54.0648600622 | 461 | 5.2e-129 |
| CAQ18833.1 | polyketide\_synthase | BGC0000954 | Ajudazol\_biosynthetic\_gene\_cluster | 35 | 39.2270102177 | 461 | 5.2e-129 |
| AAF19813.1 | MtaE | BGC0001024 | Myxothiazol\_biosynthetic\_gene\_cluster | 34 | 41.003998223 | 460 | 1.2e-128 |
| CAD19089.1 | StiE\_protein | BGC0000153 | Stigmatellin\_biosynthetic\_gene\_cluster | 33 | 41.2261217237 | 459 | 2.6e-128 |
| CAQ43078.1 | polyketide\_synthase | BGC0000970 | Chondrochloren\_biosynthetic\_gene\_cluster | 31 | 53.0875166593 | 458 | 4.4e-128 |
| BAJ16468.1 | polyketide\_synthase | BGC0000058 | FD-891\_biosynthetic\_gene\_cluster | 30 | 52.8209684585 | 458 | 5.8e-128 |
| BAJ16470.1 | polyketide\_synthase | BGC0000058 | FD-891\_biosynthetic\_gene\_cluster | 30 | 53.2652154598 | 457 | 9.8e-128 |
| CBD77734.1 | polyketide\_synthase | BGC0000974 | Crocacin\_biosynthetic\_gene\_cluster | 33 | 40.7818747223 | 456 | 1.7e-127 |
| ABX60162.1 | polyketide\_synthase | BGC0000978 | Cylindrospermopsin\_biosynthetic\_gene\_cluster | 32 | 44.8689471346 | 456 | 1.7e-127 |
| ctg1\_orf11 |  | BGC0000053 | Elaiophylin\_biosynthetic\_gene\_cluster | 30 | 52.8653931586 | 456 | 1.7e-127 |
| AAW03328.1 | CtaE | BGC0000982 | Cystothiazole\_A\_biosynthetic\_gene\_cluster | 33 | 41.670368725 | 456 | 1.7e-127 |
| CAJ46689.1 | polyketide\_synthase | BGC0000969 | Chondramide\_biosynthetic\_gene\_cluster | 30 | 52.5988449578 | 455 | 2.9e-127 |
| AJW65409.1 | type\_I\_modular\_polyketide\_synthase | BGC0001195 | Nocardiopsin\_biosynthetic\_gene\_cluster | 31 | 52.1990226566 | 455 | 3.7e-127 |
| WP\_042799407.1 | polyketide\_synthase | BGC0001283 | Arsenopolyketides\_biosynthetic\_gene\_cluster | 32 | 48.5117725455 | 455 | 4.9e-127 |
| AAY28227.1 | HbmAIII | BGC0000074 | Herbimycin\_biosynthetic\_gene\_cluster | 30 | 52.5099955575 | 453 | 1.4e-126 |
| BAH02268.1 | polyketide\_synthase | BGC0000126 | Pladienolide\_biosynthetic\_gene\_cluster | 30 | 52.7321190582 | 453 | 1.4e-126 |
| AAG23262.1 | polyketide\_synthase\_extender\_modules\_8-10 | BGC0000148 | Spinosad\_biosynthetic\_gene\_cluster | 31 | 52.2878720569 | 453 | 1.9e-126 |
| AAF62883.1 | epoD | BGC0000991 | Epothilone\_biosynthetic\_gene\_cluster | 29 | 51.0439804531 | 453 | 1.9e-126 |
| AAZ77693.1 | ChlA1 | BGC0000036 | Chlorothricin\_biosynthetic\_gene\_cluster | 30 | 53.0430919591 | 452 | 2.4e-126 |
| AEE88284.1 | CurF | BGC0000976 | Curacin\_biosynthetic\_gene\_cluster | 29 | 53.0430919591 | 452 | 2.4e-126 |
| ADM46357.1 | polyketide\_synthase | BGC0000106 | Naphthomycin\_biosynthetic\_gene\_cluster | 33 | 44.3802754331 | 452 | 3.2e-126 |
| AAT70103.1 | CurH | BGC0001165 | Curacin\_biosynthetic\_gene\_cluster | 32 | 44.4247001333 | 452 | 3.2e-126 |
| AEE88282.1 | CurH | BGC0000976 | Curacin\_biosynthetic\_gene\_cluster | 32 | 44.4247001333 | 452 | 3.2e-126 |
| ABK32259.1 | AmbE | BGC0000014 | Ambruticin\_biosynthetic\_gene\_cluster | 30 | 52.5099955575 | 451 | 5.4e-126 |
| AEP40936.1 | polyketide\_synthase\_type\_I | BGC0000021 | Apoptolidin\_biosynthetic\_gene\_cluster | 31 | 52.9542425589 | 451 | 5.4e-126 |
| AAT70101.1 | CurF | BGC0001165 | Curacin\_biosynthetic\_gene\_cluster | 29 | 53.0430919591 | 450 | 9.2e-126 |
| CAO98879.1 | polyketide\_synthase\_AufD | BGC0000023 | Aurafuron\_biosynthetic\_gene\_cluster | 32 | 46.334962239 | 450 | 1.6e-125 |
| AAC69330.1 | type\_I\_polyketide\_synthase\_PikAII | BGC0000094 | Methymycin\_/\_pikromycin\_biosynthetic\_gene\_cluster | 29 | 52.9542425589 | 450 | 1.6e-125 |
| ABB86410.1 | GelC | BGC0000067 | Geldanamycin\_biosynthetic\_gene\_cluster | 29 | 52.5988449578 | 450 | 1.6e-125 |
| AAQ84145.1 | Plm5 | BGC0000123 | Phoslactomycin\_B\_biosynthetic\_gene\_cluster | 29 | 58.0186583741 | 450 | 1.6e-125 |
| CAE02602.1 | polyketide\_synthase\_type\_I | BGC0000024 | Aureothin\_biosynthetic\_gene\_cluster | 30 | 52.6876943581 | 450 | 1.6e-125 |
| ABI93779.1 | GdmPKS | BGC0000068 | Geldanamycin\_biosynthetic\_gene\_cluster | 30 | 52.5099955575 | 449 | 2.1e-125 |
| ACB46195.1 | polyketide\_synthase | BGC0000989 | Epothilone\_biosynthetic\_gene\_cluster | 30 | 51.4438027543 | 449 | 2.1e-125 |
| AAO06918.1 | GdmAIII | BGC0000066 | Geldanamycin\_biosynthetic\_gene\_cluster | 29 | 52.5988449578 | 449 | 2.1e-125 |
| ctg1\_orf521 |  | BGC0001199 | Akaeolide\_biosynthetic\_gene\_cluster | 30 | 52.6876943581 | 448 | 3.5e-125 |
| BAO98805.1 | putative\_polyketide\_synthase | BGC0001002 | Jawsamycin\_biosynthetic\_gene\_cluster | 34 | 41.0928476233 | 447 | 7.8e-125 |
| CBD77736.1 | polyketide\_synthase | BGC0000974 | Crocacin\_biosynthetic\_gene\_cluster | 30 | 53.8871612617 | 447 | 7.8e-125 |
| AAY42396.1 | HctD | BGC0001000 | Hectochlorin\_biosynthetic\_gene\_cluster | 34 | 41.4926699245 | 446 | 2.3e-124 |
| CUW01175.1 | Type\_I\_polyketide\_synthase | BGC0001359 | PM100117\_/\_PM100118\_biosynthetic\_gene\_cluster | 29 | 53.3984895602 | 446 | 2.3e-124 |
| AEZ54377.1 | PieA4 | BGC0000124 | Piericidin\_A1\_biosynthetic\_gene\_cluster | 31 | 52.6432696579 | 446 | 2.3e-124 |
| CAQ18834.1 | polyketide\_synthase | BGC0000954 | Ajudazol\_biosynthetic\_gene\_cluster | 34 | 41.4038205242 | 445 | 3e-124 |
| AAZ94388.1 | nodular\_polyketide\_synthase | BGC0000040 | Concanamycin\_A\_biosynthetic\_gene\_cluster | 30 | 54.0204353621 | 444 | 6.6e-124 |
| EAU29808.1 | hypothetical\_protein | BGC0001400 | Citreoviridin\_biosynthetic\_gene\_cluster | 35 | 40.6486006219 | 444 | 8.6e-124 |
| BAF02927.1 | type\_I\_polyketide\_synthase | BGC0000073 | Halstoctacosanolide\_biosynthetic\_gene\_cluster | 29 | 52.6876943581 | 443 | 1.5e-123 |
| AIT55261.1 | GulD | BGC0000072 | Gulmirecin\_biosynthetic\_gene\_cluster | 35 | 39.3158596179 | 443 | 1.5e-123 |
| ADZ24997.1 | polyketide\_synthase | BGC0000380 | Leupyrrin\_biosynthetic\_gene\_cluster | 28 | 57.5744113727 | 443 | 1.9e-123 |
| ADM46359.1 | polyketide\_synthase | BGC0000106 | Naphthomycin\_biosynthetic\_gene\_cluster | 30 | 52.7765437583 | 443 | 1.9e-123 |
| ABK32288.1 | JerB | BGC0000080 | Jerangolid\_biosynthetic\_gene\_cluster | 31 | 45.4908929365 | 443 | 1.9e-123 |
| BAC68129.1 | modular\_polyketide\_synthase | BGC0000059 | Filipin\_biosynthetic\_gene\_cluster | 29 | 55.7529986673 | 442 | 2.5e-123 |
| AAT70096.1 | CurA | BGC0001165 | Curacin\_biosynthetic\_gene\_cluster | 33 | 41.4482452243 | 442 | 3.3e-123 |
| CAD19086.1 | StiB\_protein | BGC0000153 | Stigmatellin\_biosynthetic\_gene\_cluster | 34 | 41.670368725 | 442 | 3.3e-123 |
| ABK32256.1 | AmbB | BGC0000014 | Ambruticin\_biosynthetic\_gene\_cluster | 31 | 44.6023989338 | 442 | 3.3e-123 |
| AEZ64504.1 | Herc | BGC0001065 | Herboxidiene\_biosynthetic\_gene\_cluster | 29 | 52.7765437583 | 442 | 3.3e-123 |
| AEE88289.1 | CurA | BGC0000976 | Curacin\_biosynthetic\_gene\_cluster | 33 | 41.4482452243 | 442 | 3.3e-123 |
| BAJ16469.1 | polyketide\_synthase | BGC0000058 | FD-891\_biosynthetic\_gene\_cluster | 30 | 51.3105286539 | 441 | 5.6e-123 |
| ABX60163.1 | polyketide\_synthase | BGC0000978 | Cylindrospermopsin\_biosynthetic\_gene\_cluster | 33 | 42.4255886273 | 441 | 5.6e-123 |
| ADF88275.1 | polyketide\_synthase | BGC0000981 | Cylindrospermopsin\_biosynthetic\_gene\_cluster | 33 | 41.0484229231 | 441 | 5.6e-123 |
| AEP40934.1 | polyketide\_synthase\_type\_I | BGC0000021 | Apoptolidin\_biosynthetic\_gene\_cluster | 32 | 44.4691248334 | 441 | 7.3e-123 |
| AHN85651.1 | Phn2 | BGC0000122 | Phenylnannolone\_A\_biosynthetic\_gene\_cluster | 30 | 52.7321190582 | 441 | 7.3e-123 |
| ADM46360.1 | polyketide\_synthase | BGC0000106 | Naphthomycin\_biosynthetic\_gene\_cluster | 30 | 52.6876943581 | 441 | 7.3e-123 |
| ADH04657.1 | TugA | BGC0001342 | Thuggacin\_biosynthetic\_gene\_cluster | 32 | 44.1137272323 | 441 | 7.3e-123 |
| AFV30249.1 | polyketide\_synthase | BGC0000075 | Hygrocin\_biosynthetic\_gene\_cluster | 29 | 52.9542425589 | 441 | 7.3e-123 |
| CAD29794.1 | peptide\_synthetase | BGC0001015 | Microcystin\_biosynthetic\_gene\_cluster | 33 | 41.9369169258 | 440 | 1.6e-122 |
| AAS98781.1 | JamJ | BGC0001001 | Jamaicamide\_biosynthetic\_gene\_cluster | 28 | 58.7294535762 | 439 | 2.1e-122 |
| AAP85335.1 | type\_I\_PKS | BGC0000233 | Hedamycin\_biosynthetic\_gene\_cluster | 29 | 52.5544202577 | 439 | 2.1e-122 |
| ctg1\_orf29 |  | BGC0000096 | Midecamycin\_biosynthetic\_gene\_cluster | 29 | 57.6188360729 | 439 | 2.1e-122 |
| ADF88277.1 | polyketide\_synthase | BGC0000981 | Cylindrospermopsin\_biosynthetic\_gene\_cluster | 33 | 42.4255886273 | 439 | 2.8e-122 |
| CAD19088.1 | StiD\_protein | BGC0000153 | Stigmatellin\_biosynthetic\_gene\_cluster | 33 | 40.8707241226 | 438 | 4.7e-122 |
| AIT55259.1 | GulB | BGC0000072 | Gulmirecin\_biosynthetic\_gene\_cluster | 33 | 40.7818747223 | 438 | 4.7e-122 |
| ABK32257.1 | AmbC | BGC0000014 | Ambruticin\_biosynthetic\_gene\_cluster | 30 | 52.3767214571 | 438 | 4.7e-122 |
| AJW65407.1 | type\_I\_modular\_polyketide\_synthase | BGC0001195 | Nocardiopsin\_biosynthetic\_gene\_cluster | 33 | 43.8471790315 | 438 | 6.2e-122 |
| ANF07288.1 | hrPKS | BGC0001385 | Byssochlamic\_acid\_/\_agnestadrides\_biosynthetic\_gene\_cluster | 33 | 38.6050644158 | 437 | 1.1e-121 |
| AAB66506.1 | tylactone\_synthase\_modules\_4\_&\_5 | BGC0000166 | Tylactone\_biosynthetic\_gene\_cluster | 32 | 44.5135495335 | 436 | 1.4e-121 |
| CQR60493.1 | Polyketide\_synthase,\_type\_I,\_modules:\_9\_and\_10 | BGC0001287 | Chaxamycin\_A\_/\_chaxamycin\_B\_/\_chaxamycin\_C\_/\_chaxamycin\_D | 29 | 54.1537094625 | 436 | 1.4e-121 |
| CQR60496.1 | Polyketide\_synthase,\_type\_I,\_modules:\_4,\_5\_and\_6 | BGC0001287 | Chaxamycin\_A\_/\_chaxamycin\_B\_/\_chaxamycin\_C\_/\_chaxamycin\_D | 31 | 52.2878720569 | 436 | 1.4e-121 |
| AAG23263.1 | polyketide\_synthase\_extender\_modules\_5-7 | BGC0000148 | Spinosad\_biosynthetic\_gene\_cluster | 33 | 40.8262994225 | 436 | 1.8e-121 |
| CAQ18832.1 | polyketide\_synthase | BGC0000954 | Ajudazol\_biosynthetic\_gene\_cluster | 30 | 53.2207907597 | 436 | 1.8e-121 |
| ADH04641.1 | TgaC | BGC0001051 | Thuggacin\_biosynthetic\_gene\_cluster | 30 | 53.4873389605 | 436 | 2.3e-121 |
| AMB48442.1 | polyketide\_synthase | BGC0001357 | Carbamidocyclophane\_biosynthetic\_gene\_cluster | 34 | 41.0484229231 | 436 | 2.3e-121 |
| AFU82616.1 | polyketide\_synthase | BGC0000998 | Guadinomine\_biosynthetic\_gene\_cluster | 30 | 52.5988449578 | 435 | 3.1e-121 |
| ABV97155.1 | Acyl\_transferase | BGC0000137 | Rifamycin\_biosynthetic\_gene\_cluster | 30 | 52.8653931586 | 435 | 3.1e-121 |
| ADF88276.1 | polyketide\_synthase | BGC0000981 | Cylindrospermopsin\_biosynthetic\_gene\_cluster | 28 | 51.5326521546 | 435 | 3.1e-121 |
| AFV96138.1 | polyketide\_synthase | BGC0001064 | Cylindrocyclophane\_biosynthetic\_gene\_cluster | 34 | 40.9595735229 | 435 | 5.2e-121 |
| ABX60152.1 | polyketide\_synthase | BGC0000978 | Cylindrospermopsin\_biosynthetic\_gene\_cluster | 28 | 51.5770768547 | 434 | 6.8e-121 |
| AEZ54378.1 | PieA5 | BGC0000124 | Piericidin\_A1\_biosynthetic\_gene\_cluster | 32 | 45.0466459351 | 434 | 6.8e-121 |
| BAJ16471.1 | polyketide\_synthase | BGC0000058 | FD-891\_biosynthetic\_gene\_cluster | 30 | 52.6876943581 | 434 | 8.9e-121 |
| CAO85893.1 | modular\_polyketide\_synthase\_NorA | BGC0000110 | Neoaureothin\_biosynthetic\_gene\_cluster | 30 | 52.5544202577 | 434 | 8.9e-121 |
| BAD08373.1 | polyketide\_synthase\_modules\_1-3 | BGC0000167 | Vicenistatin\_biosynthetic\_gene\_cluster | 31 | 52.4211461573 | 433 | 1.2e-120 |
| AEP40940.1 | polyketide\_synthase\_type\_I | BGC0000021 | Apoptolidin\_biosynthetic\_gene\_cluster | 30 | 52.2434473567 | 433 | 1.2e-120 |
| BAQ21939.1 | putative\_type\_I\_polyketide\_synthase | BGC0001204 | Versipelostatin\_biosynthetic\_gene\_cluster | 30 | 53.1319413594 | 433 | 1.5e-120 |
| AAF00958.1 | McyE | BGC0001017 | Microcystin\_biosynthetic\_gene\_cluster | 33 | 40.9151488227 | 433 | 1.5e-120 |
| ABX60153.1 | polyketide\_synthase | BGC0000978 | Cylindrospermopsin\_biosynthetic\_gene\_cluster | 32 | 41.003998223 | 433 | 1.5e-120 |
| AAK73502.1 | AmphJ | BGC0000015 | Amphotericin\_biosynthetic\_gene\_cluster | 29 | 55.4864504665 | 433 | 2e-120 |
| AAF62882.1 | EpoC | BGC0000991 | Epothilone\_biosynthetic\_gene\_cluster | 30 | 53.2207907597 | 433 | 2e-120 |
| AAK57190.1 | MxaF | BGC0001022 | Myxalamid\_biosynthetic\_gene\_cluster | 32 | 39.4047090182 | 432 | 2.6e-120 |
| AHB82064.1 | polyketide\_synthase | BGC0001231 | Microsclerodermins\_biosynthetic\_gene\_cluster | 34 | 40.2932030209 | 432 | 3.4e-120 |
| AAS98783.1 | JamL | BGC0001001 | Jamaicamide\_biosynthetic\_gene\_cluster | 29 | 52.1545979565 | 431 | 4.4e-120 |
| CAL58685.1 | polyketide\_synthase | BGC0000149 | Spirangien\_biosynthetic\_gene\_cluster | 33 | 40.9595735229 | 431 | 4.4e-120 |
| BAH02269.1 | polyketide\_synthase | BGC0000126 | Pladienolide\_biosynthetic\_gene\_cluster | 29 | 53.8871612617 | 431 | 5.8e-120 |
| ADB12490.1 | EpoC | BGC0000990 | Epothilone\_biosynthetic\_gene\_cluster | 30 | 53.2207907597 | 431 | 5.8e-120 |
| AEZ54379.1 | PieA6 | BGC0000124 | Piericidin\_A1\_biosynthetic\_gene\_cluster | 33 | 40.7818747223 | 431 | 5.8e-120 |
| AFV30247.1 | polyketide\_synthase | BGC0000075 | Hygrocin\_biosynthetic\_gene\_cluster | 30 | 52.2434473567 | 431 | 5.8e-120 |
| CAL58684.1 | polyketide\_synthase | BGC0000149 | Spirangien\_biosynthetic\_gene\_cluster | 33 | 41.5815193247 | 431 | 5.8e-120 |
| AEZ54374.1 | PieA1 | BGC0000124 | Piericidin\_A1\_biosynthetic\_gene\_cluster | 30 | 52.8209684585 | 431 | 5.8e-120 |
| AGI99496.1 | Type\_I\_polyketide\_synthase | BGC0001004 | Lobophorin\_biosynthetic\_gene\_cluster | 30 | 52.6876943581 | 431 | 7.5e-120 |
| ABV97153.1 | Beta-ketoacyl\_synthase | BGC0000137 | Rifamycin\_biosynthetic\_gene\_cluster | 29 | 52.4211461573 | 430 | 9.9e-120 |
| ctg1\_orf10 |  | BGC0000053 | Elaiophylin\_biosynthetic\_gene\_cluster | 30 | 51.9324744558 | 430 | 1.3e-119 |
| CAQ18828.1 | polyketide\_synthase | BGC0000954 | Ajudazol\_biosynthetic\_gene\_cluster | 29 | 53.1763660595 | 430 | 1.3e-119 |
| CAD89776.1 | MelE\_protein | BGC0001010 | Melithiazol\_biosynthetic\_gene\_cluster | 32 | 41.0928476233 | 430 | 1.3e-119 |
| CAQ43077.1 | polyketide\_synthase | BGC0000970 | Chondrochloren\_biosynthetic\_gene\_cluster | 30 | 54.2869835629 | 430 | 1.3e-119 |
| CBA11584.1 | polyketide\_synthase\_type\_I | BGC0001046 | Streptolydigin\_biosynthetic\_gene\_cluster | 29 | 51.4882274545 | 430 | 1.3e-119 |
| AFI57006.1 | QmnA2 | BGC0000133 | Quartromicin\_biosynthetic\_gene\_cluster | 29 | 53.0430919591 | 430 | 1.3e-119 |
| ACB46194.1 | polyketide\_synthase | BGC0000989 | Epothilone\_biosynthetic\_gene\_cluster | 31 | 50.5108840515 | 430 | 1.7e-119 |
| AAF26920.1 | polyketide\_synthase | BGC0000988 | Epothilone\_biosynthetic\_gene\_cluster | 30 | 53.2207907597 | 430 | 1.7e-119 |
| CAD19085.1 | StiA\_protein | BGC0000153 | Stigmatellin\_biosynthetic\_gene\_cluster | 34 | 37.7609951133 | 429 | 2.2e-119 |
| AFU82617.1 | polyketide\_synthase | BGC0000998 | Guadinomine\_biosynthetic\_gene\_cluster | 32 | 45.1354953354 | 429 | 2.2e-119 |
| ADX66472.1 | ScnS1 | BGC0000108 | Natamycin\_biosynthetic\_gene\_cluster | 30 | 53.0875166593 | 429 | 2.2e-119 |
| AAC01714.1 | RifE | BGC0000136 | Rifamycin\_biosynthetic\_gene\_cluster | 30 | 52.7765437583 | 429 | 2.2e-119 |
| AAG23266.1 | polyketide\_synthase\_extender\_modules\_3-4 | BGC0000148 | Spinosad\_biosynthetic\_gene\_cluster | 32 | 40.8707241226 | 429 | 2.9e-119 |
| AAS98782.1 | JamK | BGC0001001 | Jamaicamide\_biosynthetic\_gene\_cluster | 34 | 37.9386939138 | 429 | 2.9e-119 |
| BAO66529.1 | type\_I\_polyketide\_synthase | BGC0000042 | Cremimycin\_biosynthetic\_gene\_cluster | 30 | 53.3096401599 | 429 | 2.9e-119 |
| ACR33079.1 | polyketide\_synthase | BGC0000017 | Anatoxin\_biosynthetic\_gene\_cluster | 32 | 41.4482452243 | 428 | 3.7e-119 |
| ACN64831.1 | PokM1 | BGC0001061 | Polyketomycin\_biosynthetic\_gene\_cluster | 32 | 44.7800977343 | 428 | 3.7e-119 |
| CAC22144.1 | putative\_type\_I\_polyketide\_synthase | BGC0000038 | Coelimycin\_biosynthetic\_gene\_cluster | 32 | 44.7356730342 | 428 | 4.9e-119 |
| ADC79618.1 | BafAIII | BGC0000028 | Bafilomycin\_biosynthetic\_gene\_cluster | 30 | 52.4655708574 | 428 | 6.4e-119 |
| ABB05103.1 | LipPks2 | BGC0001003 | Lipomycin\_biosynthetic\_gene\_cluster | 31 | 52.5099955575 | 427 | 8.3e-119 |
| AEZ53945.1 | polyketide\_synthase | BGC0000144 | Salinomycin\_biosynthetic\_gene\_cluster | 32 | 45.1354953354 | 427 | 1.1e-118 |
| ADH04660.1 | TugD | BGC0001342 | Thuggacin\_biosynthetic\_gene\_cluster | 30 | 53.6206130609 | 427 | 1.1e-118 |
| ACB37740.1 | putative\_type\_I\_polyketide\_synthase | BGC0000162 | Tetrocarcin\_A\_biosynthetic\_gene\_cluster | 31 | 44.3802754331 | 427 | 1.1e-118 |
| AHH25595.1 | PKS | BGC0000957 | Ansatrienin\_(mycotrienin)\_biosynthetic\_gene\_cluster | 29 | 52.0657485562 | 426 | 1.4e-118 |
| AAC01711.1 | RifB | BGC0000136 | Rifamycin\_biosynthetic\_gene\_cluster | 29 | 50.9551310529 | 426 | 1.4e-118 |
| ACB46488.1 | polyketide\_synthase | BGC0000082 | Kijanimicin\_biosynthetic\_gene\_cluster | 30 | 52.2878720569 | 426 | 1.4e-118 |
| ABV97152.1 | Beta-ketoacyl\_synthase | BGC0000137 | Rifamycin\_biosynthetic\_gene\_cluster | 29 | 52.9098178587 | 426 | 2.4e-118 |
| ADC79619.1 | BafAIV | BGC0000028 | Bafilomycin\_biosynthetic\_gene\_cluster | 30 | 52.7321190582 | 425 | 3.2e-118 |
| ACN69988.1 | polyketide\_synthase | BGC0000079 | Indanomycin\_biosynthetic\_gene\_cluster | 30 | 52.6432696579 | 425 | 3.2e-118 |
| AAX98189.1 | polyketide\_synthase\_type\_I | BGC0000052 | ECO-02301\_biosynthetic\_gene\_cluster | 31 | 46.2016881386 | 425 | 4.1e-118 |
| BAE93730.1 | type\_I\_polyketide\_synthase | BGC0000164 | Tetronomycin\_biosynthetic\_gene\_cluster | 29 | 53.5761883607 | 425 | 5.4e-118 |
| CAQ52622.1 | type\_I\_polyketide\_synthase,\_modules\_4-5 | BGC0001066 | Kendomycin\_biosynthetic\_gene\_cluster | 30 | 52.4211461573 | 425 | 5.4e-118 |
| AEH42491.1 | polyketide\_synthase | BGC0000032 | Calcimycin\_biosynthetic\_gene\_cluster | 31 | 44.0248778321 | 424 | 7.1e-118 |
| WP\_043476519.1 | polyketide\_synthase | BGC0001232 | Leucanicidin\_biosynthetic\_gene\_cluster | 30 | 53.5761883607 | 424 | 7.1e-118 |
| BAK64638.1 | polyketide\_synthase | BGC0000135 | Reveromycin\_biosynthetic\_gene\_cluster | 29 | 54.2425588627 | 424 | 9.2e-118 |
| AEZ53946.1 | polyketide\_synthase | BGC0000144 | Salinomycin\_biosynthetic\_gene\_cluster | 32 | 44.0693025322 | 424 | 9.2e-118 |
| CAC22145.1 | putative\_type\_I\_polyketide\_synthase | BGC0000038 | Coelimycin\_biosynthetic\_gene\_cluster | 29 | 53.3096401599 | 423 | 1.2e-117 |
| AAT70108.1 | CurM | BGC0001165 | Curacin\_biosynthetic\_gene\_cluster | 32 | 41.7592181253 | 423 | 1.2e-117 |
| AEE88277.1 | CurM | BGC0000976 | Curacin\_biosynthetic\_gene\_cluster | 32 | 41.7592181253 | 423 | 1.2e-117 |
| ACV42478.1 | polyketide\_synthase | BGC0000043 | Curacin\_A\_biosynthetic\_gene\_cluster | 32 | 41.7592181253 | 423 | 1.2e-117 |
| AFI57005.1 | QmnA1 | BGC0000133 | Quartromicin\_biosynthetic\_gene\_cluster | 29 | 52.9542425589 | 423 | 1.6e-117 |
| AAM70355.1 | CalO5 | BGC0000033 | Calicheamicin\_biosynthetic\_gene\_cluster | 32 | 43.8027543314 | 423 | 1.6e-117 |
| AAX98191.1 | polyketide\_synthase\_type\_I | BGC0000052 | ECO-02301\_biosynthetic\_gene\_cluster | 29 | 53.8427365615 | 423 | 2.1e-117 |
| ABB88533.1 | polyketide\_synthase\_type\_I | BGC0000049 | E-492\_/\_E-975\_biosynthetic\_gene\_cluster | 32 | 41.1372723234 | 422 | 2.7e-117 |
| AAO62582.1 | polyketide\_synthase\_peptide\_sythetase\_fusion\_protein | BGC0001016 | Microcystin\_biosynthetic\_gene\_cluster | 32 | 41.7592181253 | 422 | 2.7e-117 |
| CCE88381.1 | polyketide\_synthase | BGC0001034 | Pellasoren\_biosynthetic\_gene\_cluster | 29 | 52.8209684585 | 422 | 2.7e-117 |
| BAQ25512.1 | type\_I\_polyketide\_synthase | BGC0001288 | Maklamicin\_biosynthetic\_gene\_cluster | 30 | 52.4655708574 | 422 | 2.7e-117 |
| AEU11005.1 | NpnA | BGC0001029 | Nostophycin\_biosynthetic\_gene\_cluster | 33 | 41.0484229231 | 422 | 3.5e-117 |
| CAO98849.1 | polyketide\_synthase\_AufF | BGC0000023 | Aurafuron\_biosynthetic\_gene\_cluster | 32 | 46.4238116393 | 422 | 3.5e-117 |
| AGY30677.1 | Ann5 | BGC0001298 | 4-Z-annimycin\_biosynthetic\_gene\_cluster | 32 | 44.6912483341 | 422 | 3.5e-117 |
| BAC76492.1 | lankamycin\_synthase\_LkmAII | BGC0000085 | Lankamycin\_biosynthetic\_gene\_cluster | 33 | 40.6486006219 | 422 | 3.5e-117 |
| AAF19814.1 | MtaF | BGC0001024 | Myxothiazol\_biosynthetic\_gene\_cluster | 31 | 44.1137272323 | 422 | 3.5e-117 |
| AAS98777.1 | JamE | BGC0001001 | Jamaicamide\_biosynthetic\_gene\_cluster | 32 | 40.9595735229 | 421 | 4.6e-117 |
| ABW96542.1 | type\_I\_modular\_polyketide\_synthase | BGC0000159 | Tautomycin\_biosynthetic\_gene\_cluster | 31 | 44.1581519325 | 421 | 4.6e-117 |
| AAF71767.1 | NysJ | BGC0000115 | Nystatin\_biosynthetic\_gene\_cluster | 29 | 52.7765437583 | 421 | 4.6e-117 |
| AAK73514.1 | AmphC | BGC0000015 | Amphotericin\_biosynthetic\_gene\_cluster | 29 | 52.4211461573 | 421 | 6e-117 |
| AAO65806.1 | monensin\_polyketide\_synthase\_modules\_11\_and\_12 | BGC0000100 | Monensin\_biosynthetic\_gene\_cluster | 31 | 44.6023989338 | 421 | 7.8e-117 |
| AFV30250.1 | polyketide\_synthase | BGC0000075 | Hygrocin\_biosynthetic\_gene\_cluster | 28 | 53.0875166593 | 420 | 1e-116 |
| CAQ34928.1 | polyketide\_synthase | BGC0000986 | Dkxanthene\_biosynthetic\_gene\_cluster | 35 | 36.4282541093 | 420 | 1e-116 |
| AFV30251.1 | polyketide\_synthase | BGC0000075 | Hygrocin\_biosynthetic\_gene\_cluster | 29 | 52.4211461573 | 420 | 1.3e-116 |
| BAE93731.1 | type\_I\_polyketide\_synthase | BGC0000164 | Tetronomycin\_biosynthetic\_gene\_cluster | 29 | 53.2652154598 | 420 | 1.3e-116 |
| AGY30676.1 | Ann4 | BGC0001298 | 4-Z-annimycin\_biosynthetic\_gene\_cluster | 31 | 44.1137272323 | 420 | 1.7e-116 |
| BAF02925.1 | type\_I\_polyketide\_synthase | BGC0000073 | Halstoctacosanolide\_biosynthetic\_gene\_cluster | 29 | 53.1763660595 | 420 | 1.7e-116 |
| ACR50791.1 | putative\_polyketide\_synthase | BGC0000163 | Tetronasin\_biosynthetic\_gene\_cluster | 32 | 46.2905375389 | 419 | 2.3e-116 |
| AKD43753.1 | HerB | BGC0001349 | Heronamide\_biosynthetic\_gene\_cluster | 30 | 52.0657485562 | 419 | 2.3e-116 |
| ADX66461.1 | ScnS2 | BGC0000108 | Natamycin\_biosynthetic\_gene\_cluster | 29 | 52.6876943581 | 418 | 3.9e-116 |
| ALP32046.1 | CycF | BGC0001293 | Cyclizidine\_biosynthetic\_gene\_cluster | 29 | 52.7321190582 | 418 | 3.9e-116 |
| AFL48527.1 | laidlomycin\_polyketide\_synthase\_(module\_3\_and\_module\_4) | BGC0000084 | Laidlomycin\_biosynthetic\_gene\_cluster | 29 | 53.3096401599 | 418 | 3.9e-116 |
| BAQ21940.1 | putative\_Type\_I\_polyketide\_synthase | BGC0001204 | Versipelostatin\_biosynthetic\_gene\_cluster | 29 | 56.9080408707 | 418 | 3.9e-116 |
| AAQ82567.1 | FscE | BGC0000061 | FR-008\_biosynthetic\_gene\_cluster | 30 | 53.1763660595 | 418 | 3.9e-116 |
| AAX98188.1 | polyketide\_synthase\_type\_I | BGC0000052 | ECO-02301\_biosynthetic\_gene\_cluster | 30 | 53.0430919591 | 418 | 3.9e-116 |
| CAD89773.1 | MelB\_protein | BGC0001009 | Melithiazol\_biosynthetic\_gene\_cluster | 32 | 40.9595735229 | 418 | 3.9e-116 |
| AEZ54375.1 | PieA2 | BGC0000124 | Piericidin\_A1\_biosynthetic\_gene\_cluster | 33 | 40.6041759218 | 418 | 3.9e-116 |
| AAQ82564.1 | FscC | BGC0000061 | FR-008\_biosynthetic\_gene\_cluster | 30 | 52.7765437583 | 418 | 3.9e-116 |
| AEZ53949.1 | polyketide\_synthase | BGC0000144 | Salinomycin\_biosynthetic\_gene\_cluster | 29 | 53.6206130609 | 418 | 5.1e-116 |
| CBD77732.1 | polyketide\_synthase | BGC0000974 | Crocacin\_biosynthetic\_gene\_cluster | 33 | 41.4038205242 | 418 | 5.1e-116 |
| BAK64649.1 | polyketide\_synthase | BGC0000135 | Reveromycin\_biosynthetic\_gene\_cluster | 34 | 38.5606397157 | 418 | 5.1e-116 |
| AAQ82568.1 | FscD | BGC0000061 | FR-008\_biosynthetic\_gene\_cluster | 34 | 41.0484229231 | 418 | 6.6e-116 |
| CAI94682.1 | putative\_polyketide\_synthase | BGC0000141 | Rubradirin\_biosynthetic\_gene\_cluster | 33 | 40.2932030209 | 417 | 8.6e-116 |
| AAZ77696.1 | ChlA3 | BGC0000036 | Chlorothricin\_biosynthetic\_gene\_cluster | 30 | 53.2652154598 | 417 | 8.6e-116 |
| CAC20931.1 | PimS1\_protein | BGC0000125 | Pimaricin\_biosynthetic\_gene\_cluster | 30 | 53.0430919591 | 417 | 8.6e-116 |
| AAS79460.1 | polyketide\_synthase\_subunit | BGC0000035 | Chalcomycin\_biosynthetic\_gene\_cluster | 29 | 53.9315859618 | 417 | 8.6e-116 |
| AFL48532.1 | laidlomycin\_polyketide\_synthase\_(module\_11\_and\_module\_12) | BGC0000084 | Laidlomycin\_biosynthetic\_gene\_cluster | 31 | 44.335850733 | 417 | 8.6e-116 |
| AAG23264.1 | polyketide\_synthase\_loading\_and\_extender\_module\_1 | BGC0000148 | Spinosad\_biosynthetic\_gene\_cluster | 33 | 41.003998223 | 417 | 8.6e-116 |
| CUW01176.1 | Type\_I\_polyketide\_synthase | BGC0001359\_c2 | PM100117\_/\_PM100118\_biosynthetic\_gene\_cluster | 29 | 53.665037761 | 417 | 1.1e-115 |
| ACO94483.1 | polyketide\_synthase\_type\_I | BGC0000097 | ML-449\_biosynthetic\_gene\_cluster | 31 | 47.6232785429 | 417 | 1.1e-115 |
| BAP34739.1 | type\_I\_polyketide\_synthase | BGC0000078 | Incednine\_biosynthetic\_gene\_cluster | 29 | 52.6432696579 | 416 | 1.5e-115 |
| AAM81585.1 | putative\_type\_I\_polyketide\_synthase | BGC0000047 | Dihydrochalcomycin\_biosynthetic\_gene\_cluster | 29 | 53.9315859618 | 416 | 1.5e-115 |
| AIT55264.1 | GulA | BGC0000072 | Gulmirecin\_biosynthetic\_gene\_cluster | 33 | 39.5824078187 | 416 | 1.9e-115 |
| CAJ88187.2 | putative\_type\_I\_modular\_polyketide\_synthase | BGC0000151 | Stambomycin\_biosynthetic\_gene\_cluster | 29 | 54.4646823634 | 416 | 2.5e-115 |
| ACF35445.1 | MbcAI | BGC0000090 | Macbecin\_biosynthetic\_gene\_cluster | 30 | 53.3096401599 | 416 | 2.5e-115 |
| ACR50775.1 | polyketide\_synthase | BGC0000163 | Tetronasin\_biosynthetic\_gene\_cluster | 29 | 52.2878720569 | 416 | 2.5e-115 |
| AAW03325.1 | CtaB | BGC0000982 | Cystothiazole\_A\_biosynthetic\_gene\_cluster | 34 | 38.6494891159 | 416 | 2.5e-115 |
| AMK48247.1 | 6-methylsalicylic\_acid\_synthase | BGC0001351\_c2 | Cahuitamycins\_biosynthetic\_gene\_cluster | 32 | 43.669480231 | 415 | 3.3e-115 |
| CQR60494.1 | Polyketide\_synthase,\_type\_I,\_module\_8 | BGC0001287 | Chaxamycin\_A\_/\_chaxamycin\_B\_/\_chaxamycin\_C\_/\_chaxamycin\_D | 31 | 44.1137272323 | 415 | 3.3e-115 |
| AAC01712.2 | RifC | BGC0000136 | Rifamycin\_biosynthetic\_gene\_cluster | 28 | 52.1545979565 | 415 | 3.3e-115 |
| ANC94965.1 | AlmHII | BGC0001396 | Aldgamycin\_biosynthetic\_gene\_cluster | 29 | 53.9315859618 | 415 | 3.3e-115 |
| ABC87510.1 | polyketide\_synthase | BGC0001011 | Meridamycin\_biosynthetic\_gene\_cluster | 29 | 52.5099955575 | 415 | 4.3e-115 |
| AEC13080.1 | fosB | BGC0000060 | Fostriecin\_biosynthetic\_gene\_cluster | 29 | 53.3540648601 | 415 | 4.3e-115 |
| ctg1\_orf21 |  | BGC0001013 | Meridamycin\_biosynthetic\_gene\_cluster | 29 | 52.5099955575 | 415 | 4.3e-115 |
| CAJ88176.1 | putative\_polyketide\_synthase\_B | BGC0000151 | Stambomycin\_biosynthetic\_gene\_cluster | 30 | 52.5099955575 | 415 | 5.6e-115 |
| AKD43768.1 | HerA1 | BGC0001349 | Heronamide\_biosynthetic\_gene\_cluster | 29 | 52.2878720569 | 415 | 5.6e-115 |
| BAH02270.1 | polyketide\_synthase | BGC0000126 | Pladienolide\_biosynthetic\_gene\_cluster | 29 | 52.5988449578 | 415 | 5.6e-115 |
| ACN69990.1 | polyketide\_synthase | BGC0000079 | Indanomycin\_biosynthetic\_gene\_cluster | 30 | 50.999555753 | 414 | 7.3e-115 |
| AAZ77673.1 | ChlB1 | BGC0000036 | Chlorothricin\_biosynthetic\_gene\_cluster | 32 | 44.335850733 | 414 | 9.5e-115 |
| ABY21540.1 | AngAIII | BGC0000018 | Angolamycin\_biosynthetic\_gene\_cluster | 31 | 44.7800977343 | 413 | 1.2e-114 |
| CQR60497.1 | Polyketide\_synthase,\_type\_I,\_modules:\_loading,\_1,\_2\_and\_3 | BGC0001287 | Chaxamycin\_A\_/\_chaxamycin\_B\_/\_chaxamycin\_C\_/\_chaxamycin\_D | 31 | 46.9569080409 | 413 | 1.2e-114 |
| BAR73017.1 | putative\_PKS\_(KS-AT-KR-ACP-KS-AT-DH-KR-ACP) | BGC0001194 | Hitachimycin\_biosynthetic\_gene\_cluster | 30 | 53.3096401599 | 413 | 1.2e-114 |
| AAC46026.1 | polyketide\_synthase\_modules\_4\_and\_5 | BGC0000113 | Niddamycin\_biosynthetic\_gene\_cluster | 30 | 48.9115948467 | 413 | 1.2e-114 |
| BAO66528.1 | type\_I\_polyketide\_synthase | BGC0000042 | Cremimycin\_biosynthetic\_gene\_cluster | 32 | 44.0693025322 | 413 | 1.6e-114 |
| CBA11583.1 | polyketide\_synthase\_type\_I | BGC0001046 | Streptolydigin\_biosynthetic\_gene\_cluster | 28 | 53.0430919591 | 413 | 2.1e-114 |
| CUW01191.1 | Type\_I\_polyketide\_synthase | BGC0001359\_c2 | PM100117\_/\_PM100118\_biosynthetic\_gene\_cluster | 31 | 47.001332741 | 413 | 2.1e-114 |
| WP\_043476516.1 | beta-ketoacyl\_synthase | BGC0001232 | Leucanicidin\_biosynthetic\_gene\_cluster | 30 | 52.2878720569 | 413 | 2.1e-114 |
| AAW03329.1 | CtaF | BGC0000982 | Cystothiazole\_A\_biosynthetic\_gene\_cluster | 33 | 40.8262994225 | 412 | 2.8e-114 |
| AKD43764.1 | HerF | BGC0001349 | Heronamide\_biosynthetic\_gene\_cluster | 29 | 52.3767214571 | 412 | 2.8e-114 |
| AAX98185.1 | polyketide\_synthase\_type\_I | BGC0000052 | ECO-02301\_biosynthetic\_gene\_cluster | 31 | 45.3576188361 | 412 | 2.8e-114 |
| CQR60495.1 | Polyketide\_synthase,\_type\_I,\_module\_7 | BGC0001287 | Chaxamycin\_A\_/\_chaxamycin\_B\_/\_chaxamycin\_C\_/\_chaxamycin\_D | 29 | 52.4211461573 | 412 | 3.6e-114 |
| CAC20921.1 | PimS2\_protein | BGC0000125 | Pimaricin\_biosynthetic\_gene\_cluster | 33 | 41.2261217237 | 411 | 4.7e-114 |
| BAF85843.1 | modular\_polyketide\_synthase | BGC0000109 | Nemadectin\_biosynthetic\_gene\_cluster | 29 | 53.3984895602 | 411 | 4.7e-114 |
| CBD77738.1 | polyketide\_synthase | BGC0000974 | Crocacin\_biosynthetic\_gene\_cluster | 34 | 37.5832963127 | 411 | 4.7e-114 |
| CAA60459.1 | polyketide\_synthase | BGC0001040 | Rapamycin\_biosynthetic\_gene\_cluster | 30 | 52.1101732563 | 411 | 6.2e-114 |
| ADC45515.1 | modular\_polyketide\_synthase | BGC0000093 | Meilingmycin\_biosynthetic\_gene\_cluster | 31 | 52.3767214571 | 411 | 6.2e-114 |
| AEK75503.1 | type\_1\_polyketide\_synthase | BGC0000001 | Abyssomicin\_biosynthetic\_gene\_cluster | 29 | 52.3767214571 | 411 | 6.2e-114 |
| AAF71768.1 | NysK | BGC0000115 | Nystatin\_biosynthetic\_gene\_cluster | 31 | 44.8245224345 | 411 | 6.2e-114 |
| ABB88522.1 | polyketide\_synthase\_type\_I | BGC0000050 | E-837\_biosynthetic\_gene\_cluster | 32 | 45.6241670369 | 411 | 6.2e-114 |
| AAC46024.1 | polyketide\_synthase\_modules\_1\_and\_2 | BGC0000113 | Niddamycin\_biosynthetic\_gene\_cluster | 30 | 52.5544202577 | 411 | 6.2e-114 |
| ACO94499.1 | polyketide\_synthase\_type\_I | BGC0000097 | ML-449\_biosynthetic\_gene\_cluster | 30 | 51.9768991559 | 411 | 6.2e-114 |
| BAQ21947.1 | putative\_type\_I\_polyketide\_synthase | BGC0001204 | Versipelostatin\_biosynthetic\_gene\_cluster | 31 | 44.7800977343 | 411 | 8.1e-114 |
| EPE34340.1 | Thiolase-like\_protein | BGC0001035 | Pneumocandin\_biosynthetic\_gene\_cluster | 31 | 43.4473567303 | 411 | 8.1e-114 |
| AHH34186.1 | polyketide\_synthase | BGC0001161 | 1-heptadecene\_biosynthetic\_gene\_cluster | 33 | 42.1590404265 | 411 | 8.1e-114 |
| ABC84470.1 | NIGAVIII | BGC0000114 | Nigericin\_biosynthetic\_gene\_cluster | 29 | 52.5099955575 | 410 | 1.1e-113 |
| ACO94456.1 | polyketide\_synthase\_type\_I | BGC0000029 | BE-14106\_biosynthetic\_gene\_cluster | 33 | 41.4038205242 | 410 | 1.1e-113 |
| AKD43522.1 | Type\_I\_polyketide\_synthase | BGC0001409 | Dutomycin\_biosynthetic\_gene\_cluster | 29 | 52.9542425589 | 410 | 1.4e-113 |
| ACY06287.1 | type\_I\_polyketide\_synthase | BGC0001042 | Sanglifehrin\_A\_biosynthetic\_gene\_cluster | 32 | 41.003998223 | 409 | 2.4e-113 |
| AAM81586.2 | putative\_type\_I\_polyketide\_synthase | BGC0000047 | Dihydrochalcomycin\_biosynthetic\_gene\_cluster | 31 | 44.2914260329 | 409 | 2.4e-113 |
| ACF35447.1 | MbcAIII | BGC0000090 | Macbecin\_biosynthetic\_gene\_cluster | 29 | 52.8209684585 | 409 | 3.1e-113 |
| AAY89049.1 | polyketide\_synthase | BGC0001069 | Chivosazole\_biosynthetic\_gene\_cluster | 34 | 39.9822301199 | 408 | 4e-113 |
| AHB82053.1 | polyketide\_synthase | BGC0001019 | Microsclerodermins\_biosynthetic\_gene\_cluster | 32 | 39.7601066193 | 408 | 4e-113 |
| BAR73007.1 | putative\_PKS\_(ACP-KS-AT-DH-KR-ACP-KS-AT-DH-ER-KR-ACP) | BGC0001194 | Hitachimycin\_biosynthetic\_gene\_cluster | 29 | 51.1772545535 | 408 | 4e-113 |
| AAG13918.1 | megalomicin\_6-deoxyerythronolide\_B\_synthase\_2 | BGC0000092 | Megalomicin\_biosynthetic\_gene\_cluster | 33 | 41.003998223 | 408 | 5.2e-113 |
| ADM46356.1 | polyketide\_synthase | BGC0000106 | Naphthomycin\_biosynthetic\_gene\_cluster | 31 | 44.0248778321 | 408 | 5.2e-113 |
| AIT55262.1 | GulE | BGC0000072 | Gulmirecin\_biosynthetic\_gene\_cluster | 31 | 41.1816970235 | 408 | 5.2e-113 |
| CAC20920.1 | PimS3\_protein | BGC0000125 | Pimaricin\_biosynthetic\_gene\_cluster | 32 | 43.669480231 | 408 | 6.8e-113 |
| ABB52544.1 | putative\_type\_I\_polyketide\_synthase | BGC0000047 | Dihydrochalcomycin\_biosynthetic\_gene\_cluster | 32 | 41.4482452243 | 408 | 6.8e-113 |
| BAH02271.1 | polyketide\_synthase | BGC0000126 | Pladienolide\_biosynthetic\_gene\_cluster | 32 | 41.8480675255 | 408 | 6.8e-113 |
| CAE45669.1 | borrelidin\_polyketide\_synthase,\_type\_I | BGC0000031 | Borrelidin\_biosynthetic\_gene\_cluster | 28 | 52.8653931586 | 408 | 6.8e-113 |
| ABJ97438.1 | MerB | BGC0001012 | Meridamycin\_biosynthetic\_gene\_cluster | 29 | 52.5099955575 | 407 | 8.9e-113 |
| WP\_048832936.1 | polyketide\_synthase | BGC0001348 | JBIR-100\_biosynthetic\_gene\_cluster | 29 | 54.4202576633 | 407 | 8.9e-113 |
| ABY66019.1 | 6-methylsalicylic\_acid\_synthase | BGC0001008 | Maduropeptin\_biosynthetic\_gene\_cluster | 33 | 37.7609951133 | 407 | 8.9e-113 |
| BAC76491.1 | lankamycin\_synthase\_LkmAIII | BGC0000085 | Lankamycin\_biosynthetic\_gene\_cluster | 32 | 42.1590404265 | 407 | 8.9e-113 |
| CAD89777.1 | MelF\_protein | BGC0001009 | Melithiazol\_biosynthetic\_gene\_cluster | 32 | 40.7374500222 | 407 | 8.9e-113 |
| AFU82615.1 | polyketide\_synthase | BGC0000998 | Guadinomine\_biosynthetic\_gene\_cluster | 32 | 40.6930253221 | 407 | 1.2e-112 |
| BAR73019.1 | putative\_PKS\_(KS-AT-DH-KR-ACP-TE) | BGC0001194 | Hitachimycin\_biosynthetic\_gene\_cluster | 30 | 52.2878720569 | 407 | 1.2e-112 |
| ABV97151.1 | AMP-dependent\_synthetase\_and\_ligase | BGC0000137 | Rifamycin\_biosynthetic\_gene\_cluster | 31 | 44.0248778321 | 407 | 1.2e-112 |
| ACR50774.1 | polyketide\_synthase | BGC0000163 | Tetronasin\_biosynthetic\_gene\_cluster | 29 | 53.5761883607 | 407 | 1.2e-112 |
| AGI99497.1 | type\_I\_polyketide\_synthase | BGC0001004 | Lobophorin\_biosynthetic\_gene\_cluster | 30 | 50.6885828521 | 406 | 1.5e-112 |
| AAS98200.1 | MSAS-type\_polyketide\_synthase | BGC0001273 | Asperlactone\_biosynthetic\_gene\_cluster | 29 | 44.2470013327 | 406 | 1.5e-112 |
| ACB37755.1 | putative\_type\_I\_polyketide\_synthase | BGC0000162 | Tetrocarcin\_A\_biosynthetic\_gene\_cluster | 28 | 52.7765437583 | 406 | 2e-112 |
| AGZ15473.1 | putative\_type\_I\_polyketide\_synthase | BGC0001036 | Polyoxypeptin\_biosynthetic\_gene\_cluster | 31 | 44.6023989338 | 406 | 2e-112 |
| OAQ83760.1 | polyketide\_synthase | BGC0001358 | Leucinostatins\_biosynthetic\_gene\_cluster | 31 | 44.7800977343 | 406 | 2.6e-112 |
| AGC09484.1 | LobS1 | BGC0001183 | Lobophorin\_biosynthetic\_gene\_cluster | 32 | 44.2025766326 | 405 | 3.4e-112 |
| ctg1\_orf524 |  | BGC0001199 | Akaeolide\_biosynthetic\_gene\_cluster | 32 | 40.8262994225 | 405 | 3.4e-112 |
| CAL58683.1 | polyketide\_synthase | BGC0000149 | Spirangien\_biosynthetic\_gene\_cluster | 32 | 40.7818747223 | 405 | 3.4e-112 |
| AAF71766.1 | NysI | BGC0000115 | Nystatin\_biosynthetic\_gene\_cluster | 32 | 41.670368725 | 405 | 3.4e-112 |
| AFP87523.1 | type\_I\_polyketide\_synthase | BGC0001159 | Marinopyrrole\_biosynthetic\_gene\_cluster | 33 | 39.8933807197 | 405 | 4.4e-112 |
| ABB88535.1 | polyketide\_synthase\_type\_I | BGC0000049\_c2 | E-492\_/\_E-975\_biosynthetic\_gene\_cluster | 29 | 53.0430919591 | 405 | 4.4e-112 |
| CAM00064.1 | EryAII\_Erythromycin\_polyketide\_synthase\_modules\_3\_and\_4 | BGC0000055 | Erythromycin\_biosynthetic\_gene\_cluster | 33 | 37.7609951133 | 405 | 4.4e-112 |
| AEP40939.1 | polyketide\_synthase\_type\_I | BGC0000021 | Apoptolidin\_biosynthetic\_gene\_cluster | 29 | 52.1990226566 | 405 | 4.4e-112 |
| BAK64650.1 | polyketide\_synthase | BGC0000135 | Reveromycin\_biosynthetic\_gene\_cluster | 30 | 46.2016881386 | 404 | 5.8e-112 |
| AHE80995.1 | PieA5 | BGC0001169 | Piericidin\_A1\_biosynthetic\_gene\_cluster | 31 | 44.7356730342 | 404 | 5.8e-112 |
| BAQ25511.1 | type\_I\_polyketide\_synthase | BGC0001288 | Maklamicin\_biosynthetic\_gene\_cluster | 29 | 53.3984895602 | 404 | 7.6e-112 |
| AAK73501.1 | AmphI | BGC0000015 | Amphotericin\_biosynthetic\_gene\_cluster | 32 | 41.2261217237 | 404 | 7.6e-112 |
| AEU11006.1 | NpnB | BGC0001029 | Nostophycin\_biosynthetic\_gene\_cluster | 33 | 41.4038205242 | 404 | 9.9e-112 |
| BAC57030.1 | protomycinolide\_IV\_synthase\_3 | BGC0000102 | Mycinamicin\_biosynthetic\_gene\_cluster | 28 | 57.2190137717 | 404 | 9.9e-112 |
| CUW01171.1 | Type\_I\_polyketide\_synthase | BGC0001359 | PM100117\_/\_PM100118\_biosynthetic\_gene\_cluster | 32 | 43.4917814305 | 404 | 9.9e-112 |
| AHA12079.1 | polyketide\_synthase\_type\_1 | BGC0001172 | Chlorizidine\_A\_biosynthetic\_gene\_cluster | 32 | 41.4482452243 | 404 | 9.9e-112 |
| AEC13070.1 | fosD | BGC0000060 | Fostriecin\_biosynthetic\_gene\_cluster | 32 | 41.1372723234 | 404 | 9.9e-112 |
| CAD19092.1 | StiH\_protein | BGC0000153 | Stigmatellin\_biosynthetic\_gene\_cluster | 32 | 40.8707241226 | 403 | 1.3e-111 |
| BAO66542.1 | type\_I\_polyketide\_synthase | BGC0000042 | Cremimycin\_biosynthetic\_gene\_cluster | 30 | 45.3131941359 | 403 | 1.7e-111 |
| AAG13919.1 | megalomicin\_6-deoxyerythronolide\_B\_synthase\_3 | BGC0000092 | Megalomicin\_biosynthetic\_gene\_cluster | 34 | 37.4500222124 | 403 | 1.7e-111 |
| CAD19093.1 | StiJ\_protein | BGC0000153 | Stigmatellin\_biosynthetic\_gene\_cluster | 30 | 46.2461128387 | 403 | 1.7e-111 |
| ACO94460.1 | polyketide\_synthase\_type\_I | BGC0000029 | BE-14106\_biosynthetic\_gene\_cluster | 29 | 52.332296757 | 403 | 1.7e-111 |
| AEP40935.1 | polyketide\_synthase\_type\_I | BGC0000021 | Apoptolidin\_biosynthetic\_gene\_cluster | 31 | 41.4926699245 | 403 | 2.2e-111 |
| CCP20047.1 | divK\_protein | BGC0001119 | Divergolide\_biosynthetic\_gene\_cluster | 33 | 41.4482452243 | 403 | 2.2e-111 |
| ANC94964.1 | AlmHIII | BGC0001396 | Aldgamycin\_biosynthetic\_gene\_cluster | 31 | 44.2914260329 | 402 | 2.9e-111 |
| AJW65410.1 | type\_I\_modular\_polyketide\_synthase | BGC0001195 | Nocardiopsin\_biosynthetic\_gene\_cluster | 29 | 52.4211461573 | 402 | 2.9e-111 |
| AJW65408.1 | type\_I\_modular\_polyketide\_synthase | BGC0001195 | Nocardiopsin\_biosynthetic\_gene\_cluster | 34 | 37.4944469125 | 402 | 2.9e-111 |
| AAZ94387.1 | modular\_polyketide\_synthase | BGC0000040 | Concanamycin\_A\_biosynthetic\_gene\_cluster | 33 | 37.316748112 | 401 | 4.9e-111 |
| BAD08358.1 | polyketide\_synthase\_modules\_4 | BGC0000167 | Vicenistatin\_biosynthetic\_gene\_cluster | 30 | 46.9124833407 | 401 | 6.4e-111 |
| ABC84469.1 | NigAIX | BGC0000114 | Nigericin\_biosynthetic\_gene\_cluster | 30 | 45.668591737 | 401 | 6.4e-111 |
| ABY83164.1 | Azi26 | BGC0000960 | Azinomycin\_B\_biosynthetic\_gene\_cluster | 31 | 44.8689471346 | 401 | 6.4e-111 |
| CAL58687.1 | polyketide\_synthase | BGC0000149 | Spirangien\_biosynthetic\_gene\_cluster | 31 | 45.2243447357 | 401 | 6.4e-111 |
| BAB69195.1 | modular\_polyketide\_synthase | BGC0000117 | Oligomycin\_biosynthetic\_gene\_cluster | 33 | 37.0946246113 | 401 | 6.4e-111 |
| AEU17898.1 | putative\_type\_I\_PKS | BGC0001072 | Simocyclinone\_biosynthetic\_gene\_cluster | 32 | 41.3149711239 | 401 | 6.4e-111 |
| CAJ88177.1 | putative\_type\_I\_polyketide\_synthase | BGC0000151 | Stambomycin\_biosynthetic\_gene\_cluster | 30 | 52.2434473567 | 401 | 6.4e-111 |
| CUW01172.1 | Type\_I\_polyketide\_synthase | BGC0001359 | PM100117\_/\_PM100118\_biosynthetic\_gene\_cluster | 31 | 41.4926699245 | 401 | 8.4e-111 |
| AHH99925.1 | PKS\_I | BGC0000002 | Aculeximycin\_biosynthetic\_gene\_cluster | 29 | 52.5988449578 | 401 | 8.4e-111 |
| WP\_063764077.1 | hypothetical\_protein | BGC0001348 | JBIR-100\_biosynthetic\_gene\_cluster | 31 | 43.2252332297 | 401 | 8.4e-111 |
| BAC76493.1 | lankamycin\_synthase\_LkmAI | BGC0000085 | Lankamycin\_biosynthetic\_gene\_cluster | 32 | 40.6041759218 | 401 | 8.4e-111 |
| ADC79639.1 | TamAIII | BGC0001052 | Tirandamycin\_biosynthetic\_gene\_cluster | 29 | 52.8653931586 | 400 | 1.1e-110 |
| AAM81584.2 | putative\_type\_I\_polyketide\_synthase | BGC0000047 | Dihydrochalcomycin\_biosynthetic\_gene\_cluster | 33 | 41.1816970235 | 400 | 1.1e-110 |
| CAE46843.1 | Type\_I\_modular\_polyketide\_synthase | BGC0000103 | Mycolactone\_biosynthetic\_gene\_cluster | 31 | 44.9133718347 | 400 | 1.1e-110 |
| CAE46851.1 | Type\_I\_modular\_polyketide\_synthase | BGC0000103 | Mycolactone\_biosynthetic\_gene\_cluster | 31 | 44.9133718347 | 400 | 1.1e-110 |
| AAO65796.1 | monensin\_polyketide\_synthase\_loading\_module\_and\_module\_1 | BGC0000100 | Monensin\_biosynthetic\_gene\_cluster | 29 | 53.3096401599 | 400 | 1.4e-110 |
| CAO85896.1 | protein\_modular\_polyketide\_synthase\_NorA' | BGC0000110 | Neoaureothin\_biosynthetic\_gene\_cluster | 30 | 45.668591737 | 400 | 1.4e-110 |
| ctg1\_orf7 |  | BGC0000053 | Elaiophylin\_biosynthetic\_gene\_cluster | 32 | 41.3149711239 | 399 | 1.9e-110 |
| AAC46025.1 | polyketide\_synthase\_module\_3 | BGC0000113 | Niddamycin\_biosynthetic\_gene\_cluster | 31 | 44.2470013327 | 399 | 1.9e-110 |
| AAF71776.1 | NysC | BGC0000115 | Nystatin\_biosynthetic\_gene\_cluster | 29 | 52.5544202577 | 399 | 1.9e-110 |
| AAU93806.2 | polyketide\_synthase\_modules\_3\_and\_4 | BGC0000054 | Erythromycin\_biosynthetic\_gene\_cluster | 33 | 38.9160373167 | 399 | 2.4e-110 |
| CAM00065.1 | EryAIII\_Erythromycin\_polyketide\_synthase\_modules\_5\_and\_6 | BGC0000055 | Erythromycin\_biosynthetic\_gene\_cluster | 32 | 40.6486006219 | 399 | 2.4e-110 |
| ADC45534.1 | modular\_polyketide\_synthase | BGC0000093 | Meilingmycin\_biosynthetic\_gene\_cluster | 32 | 42.4255886273 | 399 | 2.4e-110 |
| ADX66459.1 | ScnS4 | BGC0000108 | Natamycin\_biosynthetic\_gene\_cluster | 31 | 44.0248778321 | 399 | 2.4e-110 |
| ABJ97437.1 | MerA | BGC0001012 | Meridamycin\_biosynthetic\_gene\_cluster | 32 | 43.4473567303 | 399 | 3.2e-110 |
| ANC94966.1 | AlmHI | BGC0001396 | Aldgamycin\_biosynthetic\_gene\_cluster | 32 | 41.5815193247 | 399 | 3.2e-110 |
| AAK73503.1 | AmphK | BGC0000015 | Amphotericin\_biosynthetic\_gene\_cluster | 32 | 44.2025766326 | 399 | 3.2e-110 |
| ADH04658.1 | TugB | BGC0001342 | Thuggacin\_biosynthetic\_gene\_cluster | 31 | 41.3149711239 | 399 | 3.2e-110 |
| AEH42490.1 | polyketide\_synthase | BGC0000032 | Calcimycin\_biosynthetic\_gene\_cluster | 31 | 40.5597512217 | 399 | 3.2e-110 |
| AFL48525.1 | laidlomycin\_polyketide\_synthase\_(loading\_module\_and\_module\_1) | BGC0000084 | Laidlomycin\_biosynthetic\_gene\_cluster | 28 | 52.4655708574 | 399 | 3.2e-110 |
| AAS79461.1 | polyketide\_synthase\_subunit | BGC0000035 | Chalcomycin\_biosynthetic\_gene\_cluster | 30 | 44.4691248334 | 398 | 4.2e-110 |
| AAQ84156.1 | Plm1 | BGC0000123 | Phoslactomycin\_B\_biosynthetic\_gene\_cluster | 29 | 54.2869835629 | 398 | 4.2e-110 |
| AGC09499.1 | LobS4 | BGC0001183 | Lobophorin\_biosynthetic\_gene\_cluster | 30 | 47.0457574411 | 398 | 5.4e-110 |
| ABB52545.1 | putative\_type\_I\_polyketide\_synthase | BGC0000047 | Dihydrochalcomycin\_biosynthetic\_gene\_cluster | 32 | 41.8480675255 | 398 | 7.1e-110 |
| AAC69331.1 | type\_I\_polyketide\_synthase\_PikAIII | BGC0000094 | Methymycin\_/\_pikromycin\_biosynthetic\_gene\_cluster | 31 | 41.4038205242 | 397 | 9.2e-110 |
| BAE93729.1 | type\_I\_polyketide\_synthase | BGC0000164 | Tetronomycin\_biosynthetic\_gene\_cluster | 27 | 53.3096401599 | 397 | 9.2e-110 |
| AGI99482.1 | Type\_I\_polyketide\_synthase | BGC0001004 | Lobophorin\_biosynthetic\_gene\_cluster | 30 | 47.2678809418 | 397 | 1.2e-109 |
| BAC68125.1 | modular\_polyketide\_synthase | BGC0000059 | Filipin\_biosynthetic\_gene\_cluster | 32 | 40.9595735229 | 397 | 1.2e-109 |
| AAM77986.1 | iterative\_type\_I\_polyketide\_synthase | BGC0000112 | Neocarzinostatin\_biosynthetic\_gene\_cluster | 31 | 43.9360284318 | 397 | 1.2e-109 |
| AEU17897.1 | putative\_type\_I\_PKS | BGC0001072 | Simocyclinone\_biosynthetic\_gene\_cluster | 31 | 45.2243447357 | 396 | 1.6e-109 |
| ctg1\_orf523 |  | BGC0001199 | Akaeolide\_biosynthetic\_gene\_cluster | 31 | 44.2025766326 | 396 | 1.6e-109 |
| AFV96142.1 | polyketide\_synthase | BGC0001064 | Cylindrocyclophane\_biosynthetic\_gene\_cluster | 30 | 43.6250555309 | 396 | 2.1e-109 |
| ABC87509.1 | polyketide\_synthase | BGC0001011 | Meridamycin\_biosynthetic\_gene\_cluster | 30 | 53.0430919591 | 396 | 2.1e-109 |
| CAC20919.1 | PimS4\_protein | BGC0000125 | Pimaricin\_biosynthetic\_gene\_cluster | 30 | 43.9360284318 | 396 | 2.1e-109 |
| ctg1\_orf20 |  | BGC0001013 | Meridamycin\_biosynthetic\_gene\_cluster | 30 | 53.0430919591 | 396 | 2.1e-109 |
| CAE45670.1 | borrelidin\_polyketide\_synthase,\_type\_I | BGC0000031 | Borrelidin\_biosynthetic\_gene\_cluster | 31 | 40.6930253221 | 396 | 2.1e-109 |
| AAX98190.1 | polyketide\_synthase\_type\_I | BGC0000052 | ECO-02301\_biosynthetic\_gene\_cluster | 32 | 41.0928476233 | 396 | 2.7e-109 |
| ACO94488.1 | polyketide\_synthase\_type\_I | BGC0000097 | ML-449\_biosynthetic\_gene\_cluster | 29 | 52.5544202577 | 395 | 3.5e-109 |
| AAS79459.1 | polyketide\_synthase\_subunit | BGC0000035 | Chalcomycin\_biosynthetic\_gene\_cluster | 33 | 39.3158596179 | 395 | 3.5e-109 |
| ABV83223.1 | CppK | BGC0000116 | Nystatin-like\_Pseudonocardia\_polyene\_biosynthetic\_gene\_cluster | 31 | 44.0693025322 | 395 | 3.5e-109 |
| AAC01713.1 | RifD | BGC0000136 | Rifamycin\_biosynthetic\_gene\_cluster | 30 | 44.335850733 | 395 | 3.5e-109 |
| CBZ41585.1 | putative\_modular\_polyketide\_synthase | BGC0000151 | Stambomycin\_biosynthetic\_gene\_cluster | 29 | 52.5544202577 | 395 | 4.6e-109 |
| ABV83229.1 | CppB | BGC0000116 | Nystatin-like\_Pseudonocardia\_polyene\_biosynthetic\_gene\_cluster | 33 | 41.4482452243 | 395 | 4.6e-109 |
| BAP34733.1 | type\_I\_polyketide\_synthase | BGC0000078 | Incednine\_biosynthetic\_gene\_cluster | 28 | 52.9098178587 | 395 | 4.6e-109 |
| ABV83221.1 | CppI | BGC0000116 | Nystatin-like\_Pseudonocardia\_polyene\_biosynthetic\_gene\_cluster | 30 | 46.6015104398 | 395 | 4.6e-109 |
| AGZ15475.1 | putative\_type\_1\_modular\_polyketide\_synthase | BGC0001036 | Polyoxypeptin\_biosynthetic\_gene\_cluster | 33 | 37.3611728121 | 394 | 6e-109 |
| AHH99926.1 | PKS\_I | BGC0000002 | Aculeximycin\_biosynthetic\_gene\_cluster | 32 | 40.5153265215 | 394 | 6e-109 |
| ACB46471.1 | polyketide\_synthase | BGC0000082 | Kijanimicin\_biosynthetic\_gene\_cluster | 29 | 52.6432696579 | 394 | 6e-109 |
| AAM54075.1 | polyketide\_synthase | BGC0000020 | Ansamitocin\_biosynthetic\_gene\_cluster | 30 | 52.332296757 | 394 | 6e-109 |
| ANC94962.1 | AlmHV | BGC0001396 | Aldgamycin\_biosynthetic\_gene\_cluster | 32 | 41.8480675255 | 394 | 6e-109 |
| AAB66505.1 | tylactone\_synthase\_module\_3 | BGC0000166 | Tylactone\_biosynthetic\_gene\_cluster | 28 | 54.0648600622 | 394 | 6e-109 |
| ADU86003.1 | putative\_modular\_polyketide\_synthase | BGC0000165 | Tiacumicin\_B\_biosynthetic\_gene\_cluster | 32 | 37.7609951133 | 394 | 7.8e-109 |
| CAA60460.1 | polyketide\_synthase | BGC0001040 | Rapamycin\_biosynthetic\_gene\_cluster | 29 | 53.0875166593 | 394 | 7.8e-109 |
| AAF71775.1 | NysB | BGC0000115 | Nystatin\_biosynthetic\_gene\_cluster | 32 | 41.8036428254 | 394 | 7.8e-109 |
| ANC94963.1 | AlmHIV | BGC0001396 | Aldgamycin\_biosynthetic\_gene\_cluster | 32 | 41.4482452243 | 394 | 1e-108 |
| CAQ64686.1 | lasalocid\_modular\_polyketide\_synthase | BGC0000087 | Lasalocid\_biosynthetic\_gene\_cluster | 34 | 37.7609951133 | 393 | 1.3e-108 |
| AHB82051.1 | polyketide\_synthase | BGC0001019 | Microsclerodermins\_biosynthetic\_gene\_cluster | 30 | 49.0892936473 | 393 | 1.3e-108 |
| CAI94713.1 | putative\_polyketide\_synthase | BGC0000141 | Rubradirin\_biosynthetic\_gene\_cluster | 28 | 52.9098178587 | 393 | 1.7e-108 |
| AAQ84157.1 | Plm2-3 | BGC0000123 | Phoslactomycin\_B\_biosynthetic\_gene\_cluster | 31 | 42.9142603287 | 393 | 2.3e-108 |
| AEZ53952.1 | polyketide\_synthase | BGC0000144 | Salinomycin\_biosynthetic\_gene\_cluster | 30 | 45.8907152377 | 393 | 2.3e-108 |
| ALP32043.1 | CycC | BGC0001293 | Cyclizidine\_biosynthetic\_gene\_cluster | 29 | 50.3776099511 | 393 | 2.3e-108 |
| BAQ25507.1 | type\_I\_polyketide\_synthase | BGC0001288 | Maklamicin\_biosynthetic\_gene\_cluster | 33 | 38.5606397157 | 393 | 2.3e-108 |
| BAO66519.1 | type\_I\_polyketide\_synthase | BGC0000042 | Cremimycin\_biosynthetic\_gene\_cluster | 29 | 52.5544202577 | 392 | 3e-108 |
| ACN69992.1 | polyketide\_synthase | BGC0000079 | Indanomycin\_biosynthetic\_gene\_cluster | 32 | 41.670368725 | 392 | 3.9e-108 |
| CAD55506.1 | putative\_type\_I\_polyketide\_synthase | BGC0000038 | Coelimycin\_biosynthetic\_gene\_cluster | 31 | 44.1137272323 | 392 | 3.9e-108 |
| AEZ53947.1 | polyketide\_synthase | BGC0000144 | Salinomycin\_biosynthetic\_gene\_cluster | 31 | 43.8471790315 | 392 | 3.9e-108 |
| ACO94471.1 | polyketide\_synthase\_type\_I | BGC0000029 | BE-14106\_biosynthetic\_gene\_cluster | 30 | 51.6215015549 | 392 | 3.9e-108 |
| AAC69332.1 | type\_I\_polyketide\_synthase\_PikAIV | BGC0000094 | Methymycin\_/\_pikromycin\_biosynthetic\_gene\_cluster | 31 | 40.5597512217 | 391 | 5.1e-108 |
| ACY13415.1 | KR\_domain\_protein | BGC0001367 | Haliamide\_biosynthetic\_gene\_cluster | 33 | 39.9822301199 | 391 | 5.1e-108 |
| AEC13079.1 | fosA | BGC0000060 | Fostriecin\_biosynthetic\_gene\_cluster | 28 | 52.998667259 | 391 | 5.1e-108 |
| ADU86002.1 | putative\_modular\_polyketide\_synthase | BGC0000165 | Tiacumicin\_B\_biosynthetic\_gene\_cluster | 29 | 52.1990226566 | 391 | 5.1e-108 |
| AAZ94390.1 | modular\_polyketide\_synthase | BGC0000040 | Concanamycin\_A\_biosynthetic\_gene\_cluster | 30 | 44.2470013327 | 391 | 8.7e-108 |
| ABK32263.1 | AmbH | BGC0000014 | Ambruticin\_biosynthetic\_gene\_cluster | 30 | 41.3593958241 | 391 | 8.7e-108 |
| AAC68815.1 | FK506\_polyketide\_synthase | BGC0000353 | FK506\_biosynthetic\_gene\_cluster | 31 | 45.9795646379 | 391 | 8.7e-108 |
| ADC79638.1 | TamAII | BGC0001052 | Tirandamycin\_biosynthetic\_gene\_cluster | 31 | 41.4482452243 | 391 | 8.7e-108 |
| ACB37743.1 | putative\_type\_I\_polyketide\_synthase | BGC0000162 | Tetrocarcin\_A\_biosynthetic\_gene\_cluster | 31 | 41.5370946246 | 390 | 1.1e-107 |
| BAA20102.2 | 6-methylsalicylic\_acid\_synthase | BGC0001276 | 6-methylsalicyclic\_acid\_biosynthetic\_gene\_cluster | 29 | 48.4673478454 | 390 | 1.1e-107 |
| CBA11582.1 | polyketide\_synthase\_type\_I | BGC0001046 | Streptolydigin\_biosynthetic\_gene\_cluster | 32 | 40.9595735229 | 390 | 1.1e-107 |
| AJO72736.1 | Type\_I\_modular\_polyketide\_synthase | BGC0001381 | Brasilinolide\_biosynthetic\_gene\_cluster | 32 | 41.4038205242 | 390 | 1.1e-107 |
| ABC87512.1 | polyketide\_synthase | BGC0001011 | Meridamycin\_biosynthetic\_gene\_cluster | 31 | 44.0693025322 | 390 | 1.1e-107 |
| ctg1\_orf23 |  | BGC0001013 | Meridamycin\_biosynthetic\_gene\_cluster | 31 | 44.0693025322 | 390 | 1.1e-107 |
| ctg1\_orf254 |  | BGC0001200 | Lorneic\_acid\_A\_biosynthetic\_gene\_cluster | 31 | 44.0248778321 | 390 | 1.5e-107 |
| BAQ25483.1 | type\_I\_polyketide\_synthase | BGC0001288 | Maklamicin\_biosynthetic\_gene\_cluster | 33 | 39.2270102177 | 390 | 1.5e-107 |
| ABJ97439.1 | MerC | BGC0001012 | Meridamycin\_biosynthetic\_gene\_cluster | 30 | 46.0684140382 | 390 | 1.5e-107 |
| AAC38075.1 | polyketide\_synthase\_type\_I | BGC0000127 | Pyoluteorin\_biosynthetic\_gene\_cluster | 30 | 39.0937361173 | 389 | 1.9e-107 |
| AAX98184.1 | polyketide\_synthase\_type\_I | BGC0000052 | ECO-02301\_biosynthetic\_gene\_cluster | 31 | 42.0257663261 | 389 | 2.5e-107 |
| CAA60462.1 | polyketide\_synthase | BGC0001040 | Rapamycin\_biosynthetic\_gene\_cluster | 28 | 52.2434473567 | 389 | 2.5e-107 |
| AHH99920.1 | PKS\_I | BGC0000002 | Aculeximycin\_biosynthetic\_gene\_cluster | 32 | 40.8262994225 | 389 | 3.3e-107 |
| BAC68126.1 | modular\_polyketide\_synthase | BGC0000059 | Filipin\_biosynthetic\_gene\_cluster | 31 | 41.7147934251 | 389 | 3.3e-107 |
| AAC69329.1 | type\_I\_polyketide\_synthase\_PikAI | BGC0000094 | Methymycin\_/\_pikromycin\_biosynthetic\_gene\_cluster | 31 | 40.8262994225 | 388 | 4.3e-107 |
| AAP42858.1 | NanA4 | BGC0000105 | Nanchangmycin\_biosynthetic\_gene\_cluster | 28 | 47.5788538427 | 388 | 4.3e-107 |
| AAK83194.1 | polyketide\_synthase | BGC0000026 | Avilamycin\_A\_biosynthetic\_gene\_cluster | 32 | 38.7383385162 | 388 | 5.6e-107 |
| AMB48441.1 | polyketide\_synthase | BGC0001357 | Carbamidocyclophane\_biosynthetic\_gene\_cluster | 30 | 41.2261217237 | 388 | 5.6e-107 |
| ACR50773.1 | polyketide\_synthase | BGC0000163 | Tetronasin\_biosynthetic\_gene\_cluster | 31 | 44.1581519325 | 388 | 7.3e-107 |
| AEU17899.1 | putative\_type\_I\_PKS | BGC0001072 | Simocyclinone\_biosynthetic\_gene\_cluster | 33 | 41.0928476233 | 388 | 7.3e-107 |
| ctg1\_orf256 |  | BGC0001200 | Lorneic\_acid\_A\_biosynthetic\_gene\_cluster | 31 | 44.5135495335 | 388 | 7.3e-107 |
| EAU32819.1 | 6-methylsalicylic\_acid\_synthase | BGC0000160 | Terreic\_acid\_biosynthetic\_gene\_cluster | 29 | 48.4673478454 | 387 | 9.6e-107 |
| ADC45586.1 | modular\_polyketide\_synthase | BGC0000093 | Meilingmycin\_biosynthetic\_gene\_cluster | 33 | 37.2278987117 | 387 | 1.2e-106 |
| AEK75502.1 | type\_1\_polyketide\_synthase | BGC0000001 | Abyssomicin\_biosynthetic\_gene\_cluster | 29 | 52.332296757 | 387 | 1.2e-106 |
| CAD17792.1 | probable\_non\_ribosomal\_peptide\_synthetase\_protein | BGC0001363 | Ralsolamycin\_biosynthetic\_gene\_cluster | 34 | 37.4944469125 | 387 | 1.2e-106 |
| AHE80994.1 | PieA4 | BGC0001169 | Piericidin\_A1\_biosynthetic\_gene\_cluster | 31 | 44.9133718347 | 386 | 1.6e-106 |
| ctg1\_orf30 |  | BGC0000096 | Midecamycin\_biosynthetic\_gene\_cluster | 32 | 42.7809862283 | 386 | 2.1e-106 |
| ABC84458.1 | NigAIII | BGC0000114 | Nigericin\_biosynthetic\_gene\_cluster | 27 | 58.7738782763 | 386 | 2.1e-106 |
| AAD03048.1 | type\_I\_polyketide\_synthase | BGC0000041 | Coronafacic\_acid\_biosynthetic\_gene\_cluster | 30 | 44.1137272323 | 385 | 3.6e-106 |
| CAD89775.1 | MelD\_protein | BGC0001010 | Melithiazol\_biosynthetic\_gene\_cluster | 27 | 54.1981341626 | 385 | 3.6e-106 |
| AAC46027.1 | polyketide\_synthase\_module\_6 | BGC0000113 | Niddamycin\_biosynthetic\_gene\_cluster | 32 | 42.6477121279 | 385 | 3.6e-106 |
| CAO85897.1 | modular\_polyketide\_synthase\_NorB | BGC0000110 | Neoaureothin\_biosynthetic\_gene\_cluster | 30 | 44.3802754331 | 385 | 3.6e-106 |
| ABP55223.1 | beta-ketoacyl\_synthase | BGC0000142 | Salinilactam\_biosynthetic\_gene\_cluster | 33 | 37.2278987117 | 385 | 3.6e-106 |
| ABB88536.1 | polyketide\_synthase\_type\_I | BGC0000049\_c2 | E-492\_/\_E-975\_biosynthetic\_gene\_cluster | 31 | 43.003109729 | 384 | 6.2e-106 |
| ALA09355.1 | type\_I\_modular\_PKS | BGC0001303 | Lobosamide\_biosynthetic\_gene\_cluster | 32 | 41.4926699245 | 384 | 6.2e-106 |
| BAG85027.1 | putative\_polyketide\_synthase | BGC0000086 | Lasalocid\_biosynthetic\_gene\_cluster | 32 | 40.9151488227 | 384 | 8.1e-106 |
| CAQ64687.1 | lasalocid\_modular\_polyketide\_synthase | BGC0000087 | Lasalocid\_biosynthetic\_gene\_cluster | 32 | 40.9151488227 | 384 | 8.1e-106 |
| AHB82065.1 | polyketide\_synthase | BGC0001231 | Microsclerodermins\_biosynthetic\_gene\_cluster | 37 | 29.5868502888 | 384 | 8.1e-106 |
| AEZ53953.1 | polyketide\_synthase | BGC0000144 | Salinomycin\_biosynthetic\_gene\_cluster | 31 | 40.9595735229 | 384 | 8.1e-106 |
| AAO65800.1 | monensin\_polyketide\_synthase\_modules\_7\_and\_8 | BGC0000100 | Monensin\_biosynthetic\_gene\_cluster | 28 | 53.7983118614 | 384 | 1.1e-105 |
| AAU93805.2 | polyketide\_synthase\_modules\_5\_and\_6 | BGC0000054 | Erythromycin\_biosynthetic\_gene\_cluster | 31 | 41.4926699245 | 384 | 1.1e-105 |
| AAS79463.1 | polyketide\_synthase\_subunit | BGC0000035 | Chalcomycin\_biosynthetic\_gene\_cluster | 32 | 41.9369169258 | 383 | 1.4e-105 |
| ACF35446.1 | MbcAII | BGC0000090 | Macbecin\_biosynthetic\_gene\_cluster | 32 | 40.9595735229 | 383 | 1.4e-105 |
| ALP32042.1 | CycB | BGC0001293 | Cyclizidine\_biosynthetic\_gene\_cluster | 30 | 44.2470013327 | 383 | 1.8e-105 |
| AHH99922.1 | PKS\_I | BGC0000002 | Aculeximycin\_biosynthetic\_gene\_cluster | 32 | 40.9151488227 | 383 | 1.8e-105 |
| AHA38203.1 | GphJ | BGC0000069 | Gephyronic\_acid\_biosynthetic\_gene\_cluster | 32 | 41.1372723234 | 383 | 2.4e-105 |
| ACR50782.1 | polyketide\_synthase | BGC0000163 | Tetronasin\_biosynthetic\_gene\_cluster | 31 | 41.7592181253 | 383 | 2.4e-105 |
| AAX98186.1 | polyketide\_synthase\_type\_I | BGC0000052 | ECO-02301\_biosynthetic\_gene\_cluster | 31 | 41.670368725 | 382 | 3.1e-105 |
| BAO66541.1 | type\_I\_polyketide\_synthase | BGC0000042 | Cremimycin\_biosynthetic\_gene\_cluster | 31 | 45.3131941359 | 382 | 3.1e-105 |
| BAO66539.1 | type\_I\_polyketide\_synthase | BGC0000042 | Cremimycin\_biosynthetic\_gene\_cluster | 31 | 41.2705464238 | 382 | 4e-105 |
| CAO98852.1 | polyketide\_synthase\_AufI | BGC0000023 | Aurafuron\_biosynthetic\_gene\_cluster | 32 | 39.3158596179 | 382 | 4e-105 |
| OAQ83765.1 | KR\_domain-containing\_protein | BGC0001358 | Leucinostatins\_biosynthetic\_gene\_cluster | 30 | 43.8471790315 | 382 | 4e-105 |
| CAA16183.1 | polyketide\_synthase | BGC0001063 | Undecylprodigiosin\_biosynthetic\_gene\_cluster | 32 | 43.0919591293 | 381 | 5.3e-105 |
| AAU04878.1 | polyketide\_synthase | BGC0000365 | Glycopeptidolipid\_biosynthetic\_gene\_cluster | 33 | 37.4944469125 | 381 | 6.9e-105 |
| AAQ90173.1 | polyketide\_synthase\_type\_I | BGC0000128 | Pyoluteorin\_biosynthetic\_gene\_cluster | 30 | 40.6486006219 | 381 | 6.9e-105 |
| ABY21538.1 | AngAI | BGC0000018 | Angolamycin\_biosynthetic\_gene\_cluster | 32 | 40.6486006219 | 381 | 6.9e-105 |
| CAL58686.1 | polyketide\_synthase | BGC0000149 | Spirangien\_biosynthetic\_gene\_cluster | 32 | 41.3593958241 | 380 | 1.2e-104 |
| AAQ82565.1 | FscB | BGC0000061 | FR-008\_biosynthetic\_gene\_cluster | 32 | 41.003998223 | 380 | 1.2e-104 |
| CAQ34918.1 | nonribosomal\_peptide\_synthetase/\_polyketide\_synthase | BGC0000986 | Dkxanthene\_biosynthetic\_gene\_cluster | 27 | 51.665926255 | 380 | 1.5e-104 |
| ctg1\_orf253 |  | BGC0001200 | Lorneic\_acid\_A\_biosynthetic\_gene\_cluster | 31 | 44.3802754331 | 379 | 2e-104 |
| BI06\_RS39060 | polyketide\_synthase | BGC0001232 | Leucanicidin\_biosynthetic\_gene\_cluster | 31 | 42.9586850289 | 379 | 2e-104 |
| AAK73513.1 | AmphB | BGC0000015 | Amphotericin\_biosynthetic\_gene\_cluster | 31 | 41.2261217237 | 379 | 2.6e-104 |
| AHA38199.1 | GphF | BGC0000069 | Gephyronic\_acid\_biosynthetic\_gene\_cluster | 28 | 52.5544202577 | 379 | 2.6e-104 |
| BAQ21948.1 | putative\_type\_I\_polyketide\_synthase | BGC0001204 | Versipelostatin\_biosynthetic\_gene\_cluster | 31 | 41.5370946246 | 379 | 2.6e-104 |
| CAJ88184.1 | putative\_modular\_polyketide\_synthase | BGC0000151 | Stambomycin\_biosynthetic\_gene\_cluster | 31 | 42.1146157263 | 379 | 3.4e-104 |
| ctg1\_orf9 |  | BGC0000053 | Elaiophylin\_biosynthetic\_gene\_cluster | 31 | 41.4926699245 | 379 | 3.4e-104 |
| AAS79462.1 | polyketide\_synthase\_subunit | BGC0000035 | Chalcomycin\_biosynthetic\_gene\_cluster | 31 | 41.2705464238 | 379 | 3.4e-104 |
| ABO15860.1 | polyketide\_synthase | BGC0000130 | Pyrrolomycin\_biosynthetic\_gene\_cluster | 33 | 37.5832963127 | 379 | 3.4e-104 |
| ABV99085.1 | thioester\_reductase\_domain | BGC0001007 | Lymphostin\_biosynthetic\_gene\_cluster | 33 | 38.0275433141 | 378 | 4.4e-104 |
| BAF85839.1 | modular\_polyketide\_synthase | BGC0000109 | Nemadectin\_biosynthetic\_gene\_cluster | 30 | 42.0257663261 | 378 | 5.8e-104 |
| AEZ64505.1 | Herb | BGC0001065 | Herboxidiene\_biosynthetic\_gene\_cluster | 32 | 39.4491337183 | 378 | 5.8e-104 |
| IF55\_RS36525 | polyketide\_synthase | BGC0001348 | JBIR-100\_biosynthetic\_gene\_cluster | 31 | 40.8707241226 | 378 | 7.6e-104 |
| AAQ84147.1 | Plm7 | BGC0000123 | Phoslactomycin\_B\_biosynthetic\_gene\_cluster | 32 | 40.8262994225 | 378 | 7.6e-104 |
| AIG62146.1 | 6-methylsalicylic\_acid\_synthase | BGC0000120 | Patulin\_biosynthetic\_gene\_cluster | 30 | 44.1581519325 | 377 | 9.9e-104 |
| AEK75504.1 | type\_1\_polyketide\_synthase | BGC0000001 | Abyssomicin\_biosynthetic\_gene\_cluster | 31 | 43.4473567303 | 377 | 9.9e-104 |
| CAN89636.1 | putative\_polyketide\_synthase | BGC0001070 | Kirromycin\_biosynthetic\_gene\_cluster | 31 | 44.5135495335 | 377 | 1.3e-103 |
| ABP55222.1 | beta-ketoacyl\_synthase | BGC0000142 | Salinilactam\_biosynthetic\_gene\_cluster | 33 | 37.2278987117 | 377 | 1.3e-103 |
| AEC13069.1 | fosC | BGC0000060 | Fostriecin\_biosynthetic\_gene\_cluster | 33 | 37.316748112 | 377 | 1.3e-103 |
| AAS98784.1 | JamM | BGC0001001 | Jamaicamide\_biosynthetic\_gene\_cluster | 31 | 38.4717903154 | 377 | 1.3e-103 |
| BAG84248.1 | putative\_polyketide\_synthase | BGC0000257 | Prodigiosin\_biosynthetic\_gene\_cluster | 32 | 39.4047090182 | 377 | 1.3e-103 |
| AAG13917.1 | megalomicin\_6-deoxyerythronolide\_B\_synthase\_1 | BGC0000092 | Megalomicin\_biosynthetic\_gene\_cluster | 32 | 38.0719680142 | 376 | 2.2e-103 |
| AAC01710.1 | RifA | BGC0000136 | Rifamycin\_biosynthetic\_gene\_cluster | 31 | 41.670368725 | 376 | 2.2e-103 |
| AAF86393.1 | FkbB | BGC0000994 | FK520\_biosynthetic\_gene\_cluster | 31 | 43.8027543314 | 376 | 2.9e-103 |
| CUI25672.1 | Thiolactomycin\_Hybrid\_PKS-NRPS\_system | BGC0001353 | Thiolactomycin\_biosynthetic\_gene\_cluster | 30 | 44.1581519325 | 376 | 2.9e-103 |
| CAQ52626.1 | type\_I\_polyketide\_synthase,\_loading\_module\_and\_modules\_1-3 | BGC0001066 | Kendomycin\_biosynthetic\_gene\_cluster | 33 | 38.338516215 | 375 | 4.9e-103 |
| ABP55493.1 | thioester\_reductase\_domain | BGC0001006 | Lymphostin\_biosynthetic\_gene\_cluster | 31 | 44.6912483341 | 374 | 6.4e-103 |
| AAU93807.2 | polyketide\_synthase\_modules\_1\_and\_2 | BGC0000054 | Erythromycin\_biosynthetic\_gene\_cluster | 32 | 41.1372723234 | 374 | 8.4e-103 |
| AAD43562.2 | Fum1p | BGC0000062 | Fumonisin\_biosynthetic\_gene\_cluster | 29 | 45.2243447357 | 374 | 1.1e-102 |
| ACJ24875.1 | 6-methylsalicylic\_acid\_synthase | BGC0000119 | Pactamycin\_biosynthetic\_gene\_cluster | 30 | 45.9351399378 | 373 | 1.4e-102 |
| CAJ88185.2 | putative\_modular\_polyketide\_synthase | BGC0000151 | Stambomycin\_biosynthetic\_gene\_cluster | 32 | 40.6041759218 | 373 | 1.4e-102 |
| EYT83439.1 | beta-ketoacyl\_synthase | BGC0001213 | Nataxazole\_biosynthetic\_gene\_cluster | 31 | 44.1137272323 | 373 | 1.4e-102 |
| BAF92601.1 | iterative\_type\_I\_PKS | BGC0000118 | Pactamycin\_biosynthetic\_gene\_cluster | 30 | 45.9351399378 | 373 | 1.4e-102 |
| ABY21541.1 | AngAIV | BGC0000018 | Angolamycin\_biosynthetic\_gene\_cluster | 31 | 40.5153265215 | 373 | 1.4e-102 |
| CAQ34929.1 | putative\_polyketide\_synthase | BGC0000986 | Dkxanthene\_biosynthetic\_gene\_cluster | 31 | 42.2478898267 | 373 | 1.4e-102 |
| ABV83222.1 | CppJ | BGC0000116 | Nystatin-like\_Pseudonocardia\_polyene\_biosynthetic\_gene\_cluster | 31 | 43.5806308307 | 373 | 1.9e-102 |
| AAM54076.1 | polyketide\_synthase | BGC0000020 | Ansamitocin\_biosynthetic\_gene\_cluster | 34 | 37.4500222124 | 373 | 1.9e-102 |
| BI06\_RS39075 | polyketide\_synthase | BGC0001232 | Leucanicidin\_biosynthetic\_gene\_cluster | 32 | 42.8254109285 | 371 | 5.4e-102 |
| AHA12078.1 | polyketide\_synthase\_type\_1 | BGC0001172 | Chlorizidine\_A\_biosynthetic\_gene\_cluster | 30 | 45.3131941359 | 371 | 5.4e-102 |
| AGZ15472.1 | putative\_modular\_polyketide\_synthase | BGC0001036 | Polyoxypeptin\_biosynthetic\_gene\_cluster | 31 | 40.9151488227 | 371 | 5.4e-102 |
| AIW00670.1 | mellein\_synthase | BGC0001244 | (-)-Mellein\_biosynthetic\_gene\_cluster | 29 | 47.5788538427 | 371 | 9.3e-102 |
| ctg1\_orf22 |  | BGC0001013 | Meridamycin\_biosynthetic\_gene\_cluster | 31 | 40.7374500222 | 370 | 1.2e-101 |
| ABC87511.1 | polyketide\_synthase | BGC0001011 | Meridamycin\_biosynthetic\_gene\_cluster | 31 | 40.7374500222 | 370 | 1.2e-101 |
| ABG02264.1 | SalB | BGC0000143 | Salinomycin\_biosynthetic\_gene\_cluster | 32 | 42.1590404265 | 369 | 3.5e-101 |
| CAM00062.1 | EryAI\_Erythromycin\_polyketide\_synthase\_modules\_1\_and\_2 | BGC0000055 | Erythromycin\_biosynthetic\_gene\_cluster | 30 | 42.2034651266 | 369 | 3.5e-101 |
| BAC57029.1 | protomycinolide\_IV\_synthase\_2 | BGC0000102 | Mycinamicin\_biosynthetic\_gene\_cluster | 30 | 43.8916037317 | 368 | 6e-101 |
| ACB12550.1 | Fum1 | BGC0000063 | Fumonisin\_biosynthetic\_gene\_cluster | 28 | 44.8689471346 | 368 | 7.8e-101 |
| CBD77746.1 | non-ribosomal\_peptide\_synthetase/polyketide\_synthase | BGC0000974 | Crocacin\_biosynthetic\_gene\_cluster | 29 | 48.334073745 | 367 | 1e-100 |
| ABO15861.1 | polyketide\_synthase | BGC0000130 | Pyrrolomycin\_biosynthetic\_gene\_cluster | 30 | 41.2705464238 | 367 | 1.3e-100 |
| BAD08359.1 | polyketide\_synthase\_modules\_5-6 | BGC0000167 | Vicenistatin\_biosynthetic\_gene\_cluster | 31 | 41.4038205242 | 367 | 1.3e-100 |
| ACZ65476.1 | type\_I\_modular\_polyketide\_synthase | BGC0000140 | RK-682\_biosynthetic\_gene\_cluster | 33 | 37.0501999112 | 367 | 1.3e-100 |
| ABB88519.1 | polyketide\_synthase\_type\_I | BGC0000050 | E-837\_biosynthetic\_gene\_cluster | 30 | 42.3811639271 | 366 | 1.7e-100 |
| BAR73021.1 | putative\_PKS\_(KS-AT-KR-ACP) | BGC0001194 | Hitachimycin\_biosynthetic\_gene\_cluster | 31 | 41.3149711239 | 366 | 1.7e-100 |
| AHB82063.1 | polyketide\_synthase | BGC0001231 | Microsclerodermins\_biosynthetic\_gene\_cluster | 36 | 30.0755219902 | 366 | 2.3e-100 |
| CUI25702.1 | Tu3010\_Hybrid\_PKS-NRPS\_system | BGC0001352 | Thiotetronate\_Tu\_3010\_biosynthetic\_gene\_cluster | 27 | 52.7765437583 | 366 | 3e-100 |
| AGC24271.1 | PrlQ | BGC0001038 | Pyralomicin\_biosynthetic\_gene\_cluster | 31 | 38.6050644158 | 366 | 3e-100 |
| AAF86396.1 | FkbA | BGC0000994 | FK520\_biosynthetic\_gene\_cluster | 32 | 43.8916037317 | 366 | 3e-100 |
| ADC79616.1 | BafAI | BGC0000028 | Bafilomycin\_biosynthetic\_gene\_cluster | 31 | 40.8707241226 | 364 | 6.6e-100 |
| BAB69192.1 | modular\_polyketide\_synthase | BGC0000117 | Oligomycin\_biosynthetic\_gene\_cluster | 33 | 38.5606397157 | 364 | 8.7e-100 |
| CAJ88175.1 | putative\_polyketide\_synthase\_B | BGC0000151 | Stambomycin\_biosynthetic\_gene\_cluster | 31 | 42.6032874278 | 364 | 8.7e-100 |
| AHF22854.1 | MarL | BGC0000091 | Marineosin\_biosynthetic\_gene\_cluster | 32 | 39.6268325189 | 363 | 1.5e-99 |
| CUI25727.1 | Polyketide\_synthase/Nonribosomal\_peptide\_synthase | BGC0001355 | U-68204\_biosynthetic\_gene\_cluster | 27 | 53.3540648601 | 363 | 1.5e-99 |
| BAD38873.1 | polyketide\_synthase | BGC0000111 | Neocarzilin\_biosynthetic\_gene\_cluster | 29 | 44.2470013327 | 363 | 2.5e-99 |
| EHA22196.1 | polyketide\_synthase | BGC0000170 | Yanuthone\_D\_biosynthetic\_gene\_cluster | 29 | 44.6023989338 | 362 | 3.3e-99 |
| ACR50795.1 | putative\_polyketide\_synthase | BGC0000163 | Tetronasin\_biosynthetic\_gene\_cluster | 34 | 29.4091514882 | 362 | 3.3e-99 |
| AJO72742.1 | Type\_I\_modular\_polyketide\_synthase | BGC0001381 | Brasilinolide\_biosynthetic\_gene\_cluster | 32 | 37.3611728121 | 362 | 4.3e-99 |
| ACA99172.1 | Polyketide\_synthase | BGC0001160 | Nonadecene\_biosynthetic\_gene\_cluster | 31 | 40.6041759218 | 361 | 5.6e-99 |
| BAC57031.1 | protomycinolide\_IV\_synthase\_4 | BGC0000102 | Mycinamicin\_biosynthetic\_gene\_cluster | 32 | 37.7165704131 | 361 | 7.3e-99 |
| AAB66504.1 | tylactone\_synthase\_starter\_module\_and\_modules\_1\_&\_2 | BGC0000166 | Tylactone\_biosynthetic\_gene\_cluster | 30 | 41.9813416259 | 361 | 9.6e-99 |
| AAM54077.1 | polyketide\_synthase | BGC0000020 | Ansamitocin\_biosynthetic\_gene\_cluster | 31 | 40.8262994225 | 361 | 9.6e-99 |
| ALJ49910.1 | TlmH | BGC0001237 | Thiolactomycin\_biosynthetic\_gene\_cluster | 27 | 53.3096401599 | 360 | 1.3e-98 |
| CAD29795.1 | peptide\_synthetase | BGC0001015 | Microcystin\_biosynthetic\_gene\_cluster | 30 | 44.1581519325 | 360 | 1.3e-98 |
| AHB82052.1 | polyketide\_synthase | BGC0001019 | Microsclerodermins\_biosynthetic\_gene\_cluster | 35 | 30.4753442914 | 360 | 1.3e-98 |
| AHE80991.1 | PieA1 | BGC0001169 | Piericidin\_A1\_biosynthetic\_gene\_cluster | 29 | 44.9133718347 | 360 | 1.6e-98 |
| AAF00957.1 | McyG | BGC0001017 | Microcystin\_biosynthetic\_gene\_cluster | 31 | 44.6468236339 | 359 | 2.1e-98 |
| ctg1\_orf27 |  | BGC0000096 | Midecamycin\_biosynthetic\_gene\_cluster | 30 | 40.8707241226 | 359 | 2.8e-98 |
| BAG85026.1 | putative\_polyketide\_synthase | BGC0000086 | Lasalocid\_biosynthetic\_gene\_cluster | 34 | 33.984895602 | 359 | 2.8e-98 |
| ADU85988.1 | putative\_iterative\_type\_I\_polyketide\_synthase | BGC0000165 | Tiacumicin\_B\_biosynthetic\_gene\_cluster | 30 | 44.4691248334 | 358 | 6.2e-98 |
| CAE14173.1 |  | BGC0000383 | Luminmycin\_biosynthetic\_gene\_cluster | 28 | 54.331408263 | 356 | 1.8e-97 |
| AAQ82561.1 | FscA | BGC0000061 | FR-008\_biosynthetic\_gene\_cluster | 31 | 41.4038205242 | 356 | 2.4e-97 |
| AHB82054.1 | polyketide\_synthase | BGC0001019 | Microsclerodermins\_biosynthetic\_gene\_cluster | 34 | 30.6974677921 | 356 | 2.4e-97 |
| ABI91470.1 | beta-ketoacyl\_synthase | BGC0001094 | Enacyloxin\_biosynthetic\_gene\_cluster | 30 | 44.6468236339 | 356 | 3.1e-97 |
| CAQ18839.1 | hybrid\_polyketide\_synthase/nonribosomal\_polypetide\_synthetase | BGC0000954 | Ajudazol\_biosynthetic\_gene\_cluster | 32 | 41.9369169258 | 355 | 5.3e-97 |
| ADZ24995.1 | non-ribosomal\_peptide\_synthase/polyketide\_synthase | BGC0000380 | Leupyrrin\_biosynthetic\_gene\_cluster | 31 | 44.8689471346 | 355 | 5.3e-97 |
| AAN32979.1 | BarE | BGC0000962 | Barbamide\_biosynthetic\_gene\_cluster | 30 | 43.003109729 | 354 | 6.9e-97 |
| AHE80992.1 | PieA2 | BGC0001169 | Piericidin\_A1\_biosynthetic\_gene\_cluster | 28 | 45.1354953354 | 354 | 9e-97 |
| AEE88283.1 | CurG | BGC0000976 | Curacin\_biosynthetic\_gene\_cluster | 31 | 38.7383385162 | 354 | 9e-97 |
| AAT70102.1 | CurG | BGC0001165 | Curacin\_biosynthetic\_gene\_cluster | 31 | 38.7383385162 | 354 | 9e-97 |
| CAD70195.1 | non-ribosomal\_peptide\_synthetase | BGC0001047 | Syringolin\_A\_biosynthetic\_gene\_cluster | 31 | 44.6023989338 | 354 | 9e-97 |
| BAE93728.1 | type\_I\_polyketide\_synthase | BGC0000164 | Tetronomycin\_biosynthetic\_gene\_cluster | 30 | 40.7374500222 | 354 | 9e-97 |
| AAQ84144.1 | Plm4 | BGC0000123 | Phoslactomycin\_B\_biosynthetic\_gene\_cluster | 31 | 39.5379831186 | 354 | 1.2e-96 |
| CCE67070.1 | polyketide\_synthase | BGC0001242 | Fusarubin\_biosynthetic\_gene\_cluster | 27 | 53.4873389605 | 353 | 1.5e-96 |
| CCA29203.1 | non-ribosomal\_peptide\_synthetase/polyketide\_synthase | BGC0000955 | Althiomycin\_biosynthetic\_gene\_cluster | 31 | 41.3149711239 | 353 | 2e-96 |
| AHE80996.1 | PieA6 | BGC0001169 | Piericidin\_A1\_biosynthetic\_gene\_cluster | 29 | 42.3811639271 | 353 | 2e-96 |
| CAO98848.1 | polyketide\_synthase\_AufE | BGC0000023 | Aurafuron\_biosynthetic\_gene\_cluster | 35 | 30.4309195913 | 353 | 2e-96 |
| ABM21569.1 | CrpA | BGC0000975 | Cryptophycin\_biosynthetic\_gene\_cluster | 29 | 45.1799200355 | 352 | 3.4e-96 |
| AGY62753.1 | EbeA | BGC0000051 | Ebelactone\_biosynthetic\_gene\_cluster | 32 | 37.0946246113 | 352 | 3.4e-96 |
| BAP34740.1 | type\_I\_polyketide\_synthase | BGC0000078 | Incednine\_biosynthetic\_gene\_cluster | 31 | 42.8698356286 | 352 | 4.5e-96 |
| BAB69193.1 |  | BGC0000117 | Oligomycin\_biosynthetic\_gene\_cluster | 30 | 42.3811639271 | 351 | 5.8e-96 |
| CAJ76291.1 | putative\_polyketide\_synthase | BGC0000972 | Colibactin\_biosynthetic\_gene\_cluster | 31 | 37.9831186139 | 351 | 7.6e-96 |
| AAO62585.1 | peptide\_sythetase\_polyketide\_synthase\_fusion\_protein | BGC0001016 | Microcystin\_biosynthetic\_gene\_cluster | 31 | 43.3140826299 | 351 | 7.6e-96 |
| AJO72735.1 | Type\_I\_modular\_polyketide\_synthase | BGC0001381 | Brasilinolide\_biosynthetic\_gene\_cluster | 30 | 41.0484229231 | 351 | 7.6e-96 |
| AAP42860.1 | NanA6 | BGC0000105 | Nanchangmycin\_biosynthetic\_gene\_cluster | 30 | 41.4482452243 | 351 | 7.6e-96 |
| AFU82614.1 | mixed\_NRPS\_PKS | BGC0000998 | Guadinomine\_biosynthetic\_gene\_cluster | 27 | 52.7321190582 | 351 | 9.9e-96 |
| AHB82070.1 | polyketide\_synthase | BGC0001231 | Microsclerodermins\_biosynthetic\_gene\_cluster | 30 | 42.0701910262 | 349 | 2.9e-95 |
| CBF74114.1 | Conidial\_yellow\_pigment\_biosynthesis\_polyketide\_synthase\_(PKS)(EC\_2.3.1.-)\_[Source:UniProtKB/Swiss-Prot;Acc:Q03149] | BGC0000107 | Naphthopyrone\_biosynthetic\_gene\_cluster | 28 | 46.2016881386 | 348 | 6.4e-95 |
| AAX35547.1 | polyketide\_syntase\_2 | BGC0001275 | 6-methylsalicyclic\_acid\_biosynthetic\_gene\_cluster | 29 | 44.4247001333 | 348 | 6.4e-95 |
| AAO62584.1 | polyketide\_synthase\_type\_1 | BGC0001016 | Microcystin\_biosynthetic\_gene\_cluster | 33 | 31.5859617948 | 347 | 1.1e-94 |
| ABI94379.1 | tautomycetin\_biosynthetic\_PKS | BGC0000157 | Tautomycetin\_biosynthetic\_gene\_cluster | 31 | 41.1372723234 | 347 | 1.4e-94 |
| AJO72743.1 | Type\_I\_modular\_polyketide\_synthase | BGC0001381 | Brasilinolide\_biosynthetic\_gene\_cluster | 33 | 37.0501999112 | 346 | 2.4e-94 |
| ABY21542.1 | AngAV | BGC0000018 | Angolamycin\_biosynthetic\_gene\_cluster | 30 | 42.4255886273 | 346 | 3.2e-94 |
| CAF05651.1 | TubF\_protein | BGC0001053 | Tubulysin\_biosynthetic\_gene\_cluster | 31 | 38.2496668147 | 345 | 4.2e-94 |
| CAF05649.1 | TubD\_protein | BGC0001053 | Tubulysin\_biosynthetic\_gene\_cluster | 30 | 41.5370946246 | 345 | 5.4e-94 |
| AAS98787.1 | JamP | BGC0001001 | Jamaicamide\_biosynthetic\_gene\_cluster | 33 | 34.5179920036 | 345 | 5.4e-94 |
| BAE93725.1 | type\_I\_polyketide\_synthase | BGC0000164 | Tetronomycin\_biosynthetic\_gene\_cluster | 30 | 37.3611728121 | 343 | 1.6e-93 |
| CAD29793.1 | polyketide\_synthase\_type\_I | BGC0001015 | Microcystin\_biosynthetic\_gene\_cluster | 34 | 31.5859617948 | 343 | 2.1e-93 |
| ABV91286.1 | type\_I\_modular\_polyketide\_synthase | BGC0000158 | Tautomycetin\_biosynthetic\_gene\_cluster | 31 | 41.1372723234 | 343 | 2.1e-93 |
| AFP87524.1 | type\_I\_polyketide\_synthase | BGC0001159 | Marinopyrrole\_biosynthetic\_gene\_cluster | 34 | 32.341181697 | 342 | 4.6e-93 |
| AAP33839.2 | ochratoxin\_A\_polyketide\_synthase | BGC0001030 | Ochratoxin\_A\_biosynthetic\_gene\_cluster | 31 | 34.2958685029 | 341 | 7.9e-93 |
| CAD15508.1 | probable\_polyketide\_synthase\_protein | BGC0001014 | Micacocidin\_biosynthetic\_gene\_cluster | 31 | 37.4055975122 | 341 | 7.9e-93 |
| EFY94490.1 | polyketide\_synthase,\_putative | BGC0000337 | Destruxins\_biosynthetic\_gene\_cluster | 32 | 35.0955131053 | 339 | 2.3e-92 |
| BAE93739.1 | type\_I\_polyketide\_synthase-related\_protein | BGC0000164 | Tetronomycin\_biosynthetic\_gene\_cluster | 34 | 29.8533984896 | 339 | 3e-92 |
| AHB82057.1 | polyketide\_synthase | BGC0001019 | Microsclerodermins\_biosynthetic\_gene\_cluster | 32 | 34.4735673034 | 339 | 3e-92 |
| AAV97870.1 | OnnB | BGC0001105 | Onnamide\_biosynthetic\_gene\_cluster | 35 | 26.2105730786 | 339 | 3.9e-92 |
| ADH04682.1 | polyketide\_synthase | BGC0001344 | Tubulysin\_biosynthetic\_gene\_cluster | 31 | 38.2940915149 | 337 | 8.7e-92 |
| ADM79459.1 | PKS16\_protein | BGC0001266 | Grayanic\_acid\_biosynthetic\_gene\_cluster | 28 | 42.4255886273 | 336 | 1.9e-91 |
| CBF80428.1 | polyketide\_synthase,\_putative\_(JCVI) | BGC0000680 | Austinol/dehydroaustinol,\_locus\_1\_biosynthetic\_gene\_cluster | 30 | 41.1372723234 | 336 | 3.3e-91 |
| AEN83889.1 | AdaA | BGC0000156 | TAN-1612\_biosynthetic\_gene\_cluster | 29 | 44.8689471346 | 336 | 3.3e-91 |
| EAU38791.1 | hypothetical\_protein | BGC0000161 | Terrein\_biosynthetic\_gene\_cluster | 30 | 40.8262994225 | 335 | 4.3e-91 |
| ABP73645.1 | SalA | BGC0000145 | Salinosporamide\_A\_biosynthetic\_gene\_cluster | 31 | 37.3611728121 | 334 | 9.6e-91 |
| AAF00959.1 | McyD | BGC0001017 | Microcystin\_biosynthetic\_gene\_cluster | 34 | 30.5641936917 | 334 | 9.6e-91 |
| AHA38200.1 | GphG | BGC0000069 | Gephyronic\_acid\_biosynthetic\_gene\_cluster | 32 | 37.316748112 | 333 | 1.6e-90 |
| ADI24926.1 | VrtA | BGC0000168 | Viridicatumtoxin\_biosynthetic\_gene\_cluster | 29 | 41.5370946246 | 333 | 1.6e-90 |
| EAL84397.1 | polyketide\_synthase,\_putative | BGC0001118 | Endocrocin\_biosynthetic\_gene\_cluster | 29 | 41.1372723234 | 333 | 1.6e-90 |
| BAD22832.1 | polyketide\_synthase | BGC0001265 | Melanin\_biosynthetic\_gene\_cluster | 29 | 41.1372723234 | 332 | 4.8e-90 |
| ABP53498.1 | beta-ketoacyl\_synthase | BGC0001041 | Salinosporamide\_biosynthetic\_gene\_cluster | 31 | 37.3611728121 | 331 | 6.2e-90 |
| AAR87760.2 | ZmaK | BGC0001059 | Zwittermycin\_A\_biosynthetic\_gene\_cluster | 30 | 37.5388716126 | 331 | 8.1e-90 |
| AHD05619.1 | putative\_polyketide\_synthase\_subunit | BGC0001033 | Paenilamicin\_biosynthetic\_gene\_cluster | 31 | 37.7165704131 | 329 | 3.1e-89 |
| ADA69241.1 | cis-AT\_polyketide\_synthase | BGC0001071 | Nosperin\_biosynthetic\_gene\_cluster | 30 | 37.5832963127 | 328 | 5.3e-89 |
| EGD99348.1 | polyketide\_synthase | BGC0001144 | Neosartoricin\_biosynthetic\_gene\_cluster | 28 | 41.3149711239 | 328 | 6.9e-89 |
| ABI91466.1 | beta-ketoacyl\_synthase | BGC0001094 | Enacyloxin\_biosynthetic\_gene\_cluster | 30 | 41.1816970235 | 328 | 6.9e-89 |
| ALD83686.1 | tAT\_polyketide\_synthase | BGC0001300 | Anthracimycin\_biosynthetic\_gene\_cluster | 38 | 23.5450910706 | 328 | 6.9e-89 |
| CBF90097.1 | hypothetical\_protein\_similar\_to\_polyketide\_synthase\_(Broad) | BGC0001271 | Emericellin\_biosynthetic\_gene\_cluster | 29 | 37.5832963127 | 327 | 1.5e-88 |
| ACD39762.1 | non-reducing\_polyketide\_synthase | BGC0000077 | Hypothemycin\_biosynthetic\_gene\_cluster | 30 | 40.6486006219 | 326 | 3.4e-88 |
| ERM18799.1 | polyketide\_synthase | BGC0000172 | Basiliskamides\_biosynthetic\_gene\_cluster | 37 | 23.5895157708 | 325 | 4.5e-88 |
| ACD39753.1 | non-reducing\_polyketide\_synthase | BGC0000076 | Hypothemycin\_biosynthetic\_gene\_cluster | 30 | 40.6486006219 | 325 | 4.5e-88 |
| AHD05615.1 | putative\_non-ribosomal\_peptide\_ligase/\_polyketide\_synthase\_hybrid | BGC0001033 | Paenilamicin\_biosynthetic\_gene\_cluster | 31 | 37.0501999112 | 323 | 1.7e-87 |
| BAJ09789.1 | polyketide\_synthase | BGC0000146 | Solanapyrone\_biosynthetic\_gene\_cluster | 28 | 35.0955131053 | 322 | 3.8e-87 |
| CAL80821.1 | sylD-like\_NRPS/PKS | BGC0000997 | Glidobactin\_biosynthetic\_gene\_cluster | 31 | 37.9831186139 | 322 | 3.8e-87 |
| CAG23966.1 | polyketide\_synthase\_type\_I | BGC0000181 | Macrolactin\_biosynthetic\_gene\_cluster | 36 | 23.1896934696 | 321 | 6.4e-87 |
| AGO59040.1 | PtaA | BGC0000121 | Pestheic\_acid\_biosynthetic\_gene\_cluster | 29 | 41.1372723234 | 321 | 8.4e-87 |
| AAS89999.1 | PksA | BGC0000007 | Aflatoxin\_biosynthetic\_gene\_cluster | 28 | 40.8262994225 | 321 | 8.4e-87 |
| AAS90093.1 | PksA | BGC0000006 | Aflatoxin\_biosynthetic\_gene\_cluster | 28 | 40.8262994225 | 320 | 1.4e-86 |
| BAE71314.1 | polyketide\_synthase | BGC0000004 | Aflatoxin\_biosynthetic\_gene\_cluster | 28 | 40.8262994225 | 320 | 1.9e-86 |
| AAS90022.1 | PksA | BGC0000008 | Aflatoxin\_biosynthetic\_gene\_cluster | 28 | 40.8262994225 | 320 | 1.9e-86 |
| ADB12491.1 | EpoD | BGC0000990 | Epothilone\_biosynthetic\_gene\_cluster | 34 | 28.2096845846 | 319 | 2.5e-86 |
| ADH04680.1 | hybrid\_polyketide\_synthase/non-ribosomal\_peptide\_synthetase | BGC0001344 | Tubulysin\_biosynthetic\_gene\_cluster | 29 | 41.3149711239 | 319 | 2.5e-86 |
| CTQ34881.1 | AtcD;\_polyketide\_synthase,\_modules\_1-4 | BGC0001301 | Anthracimycin\_biosynthetic\_gene\_cluster | 37 | 23.32296757 | 319 | 3.2e-86 |
| CDM36726.1 | Beta-ketoacyl\_synthase | BGC0001360 | Mycophenolic\_acid\_biosynthetic\_gene\_cluster | 28 | 48.6450466459 | 319 | 4.2e-86 |
| AAS90047.1 | PksA | BGC0000009 | Aflatoxin\_biosynthetic\_gene\_cluster | 28 | 40.8262994225 | 319 | 4.2e-86 |
| AAF26921.1 | polyketide\_synthase | BGC0000988 | Epothilone\_biosynthetic\_gene\_cluster | 34 | 28.1652598845 | 318 | 5.5e-86 |
| ALD83703.1 | tAT\_polyketide\_synthase | BGC0001299 | Chlorotonil\_biosynthetic\_gene\_cluster | 37 | 23.1452687694 | 318 | 5.5e-86 |
| AIJ04683.1 | polyketide\_synthase | BGC0001383 | Macrolactin\_biosynthetic\_gene\_cluster | 36 | 23.1896934696 | 318 | 7.1e-86 |
| AJO72734.1 | Type\_I\_modular\_polyketide\_synthase | BGC0001381 | Brasilinolide\_biosynthetic\_gene\_cluster | 29 | 40.3820524211 | 317 | 1.6e-85 |
| BAV19379.1 | polyketide\_synthase | BGC0001390 | LL-Z1272beta\_biosynthetic\_gene\_cluster | 32 | 32.2967569969 | 317 | 1.6e-85 |
| AAZ95017.1 | polyketide\_synthase | BGC0000048 | Dothistromin\_biosynthetic\_gene\_cluster | 30 | 39.8933807197 | 317 | 1.6e-85 |
| AGN74892.1 | nonribosomal\_peptide\_synthetase/polyketide\_synthase\_hybrid\_protein | BGC0000459 | Griseoviridin\_/\_viridogrisein\_biosynthetic\_gene\_cluster | 36 | 25.0555308752 | 316 | 2.1e-85 |
| CAN89632.1 | putative\_polyketide\_synthase | BGC0001070 | Kirromycin\_biosynthetic\_gene\_cluster | 36 | 24.0781874722 | 316 | 2.7e-85 |
| EAL89339.1 | polyketide\_synthase,\_putative | BGC0001403 | Trypacidin\_biosynthetic\_gene\_cluster | 30 | 37.2278987117 | 316 | 3.5e-85 |
| ABO15888.1 | polyketide\_synthase | BGC0000132 | Pyrrolomycin\_biosynthetic\_gene\_cluster | 33 | 30.8751665926 | 316 | 3.5e-85 |
| AAD38786.1 | polyketide\_synthase | BGC0001257 | 1,3,6,8-tetrahydroxynaphthalene\_biosynthetic\_gene\_cluster | 29 | 41.0484229231 | 315 | 6e-85 |
| ADI59534.1 | CorL | BGC0001091 | Corallopyronin\_biosynthetic\_gene\_cluster | 36 | 25.677476677 | 314 | 1e-84 |
| CDG12864.1 | non-ribosomal\_peptide\_synthetase | BGC0001415 | Althiomycin\_biosynthetic\_gene\_cluster | 31 | 34.4291426033 | 314 | 1.3e-84 |
| AKQ22699.1 | malonyl\_CoA-acyl\_carrier\_protein\_transacylase | BGC0001186 | Misakinolide\_biosynthetic\_gene\_cluster | 36 | 24.6112838738 | 314 | 1.3e-84 |
| ctg1\_orf6 |  | BGC0001109 | Pederin\_biosynthetic\_gene\_cluster | 38 | 23.7227898712 | 313 | 1.8e-84 |
| AAS47564.1 | mixed\_type\_I\_polyketide\_synthase/nonribosomal\_peptide\_synthetase | BGC0001108 | Pederin\_biosynthetic\_gene\_cluster | 38 | 23.7227898712 | 313 | 1.8e-84 |
| AHD05614.1 | putative\_non-ribosomal\_peptide\_ligase/\_polyketide\_synthase\_hybrid | BGC0001033 | Paenilamicin\_biosynthetic\_gene\_cluster | 29 | 41.5370946246 | 313 | 1.8e-84 |
| AIU36103.1 | LglD | BGC0000180 | Legioliulin\_biosynthetic\_gene\_cluster | 37 | 22.8342958685 | 312 | 3e-84 |
| ADD82939.1 | Bat1 | BGC0001099 | Kalimantacin\_/\_batumin\_biosynthetic\_gene\_cluster | 35 | 24.9666814749 | 312 | 3.9e-84 |
| ALD83702.1 | tAT\_polyketide\_synthase | BGC0001299 | Chlorotonil\_biosynthetic\_gene\_cluster | 37 | 23.5895157708 | 312 | 5.1e-84 |
| CAG23958.2 | polyketide\_synthase | BGC0001089 | Bacillaene\_biosynthetic\_gene\_cluster | 36 | 23.2785428698 | 312 | 5.1e-84 |
| AAY89050.1 | polyketide\_synthase | BGC0001069 | Chivosazole\_biosynthetic\_gene\_cluster | 36 | 23.7672145713 | 312 | 5.1e-84 |
| EJK79843.1 | amino\_acid\_adenylation\_enzyme/thioester\_reductase\_family\_protein | BGC0000436 | Syringolin\_A\_biosynthetic\_gene\_cluster | 29 | 42.6032874278 | 311 | 6.7e-84 |
| AAN59953.1 | polyketide\_synthase\_1 | BGC0001258 | 1,8-dihydroxynaphthalene\_biosynthetic\_gene\_cluster | 27 | 43.2252332297 | 311 | 1.1e-83 |
| AAY89053.1 | polyketide\_synthase | BGC0001069 | Chivosazole\_biosynthetic\_gene\_cluster | 35 | 24.1670368725 | 311 | 1.1e-83 |
| AAF15892.2 | NosB | BGC0001028 | Nostopeptolide\_biosynthetic\_gene\_cluster | 30 | 34.4291426033 | 309 | 2.5e-83 |
| IF55\_RS32375 | beta-ketoacyl\_synthase | BGC0001348 | JBIR-100\_biosynthetic\_gene\_cluster | 33 | 26.2105730786 | 309 | 3.3e-83 |
| AMH40421.1 | PKS | BGC0001350 | Phormidolide\_biosynthetic\_gene\_cluster | 38 | 23.9004886717 | 309 | 4.3e-83 |
| AEH59109.1 | keto-hydroxyglutarate-aldolase/polyketide\_synthase | BGC0000385 | Lysobactin\_biosynthetic\_gene\_cluster | 38 | 23.6339404709 | 308 | 5.6e-83 |
| CAN89634.1 | putative\_polyketide\_synthase | BGC0001070 | Kirromycin\_biosynthetic\_gene\_cluster | 37 | 23.5895157708 | 308 | 5.6e-83 |
| AKA54627.1 | PKS | BGC0001216 | Splenocin\_biosynthetic\_gene\_cluster | 29 | 41.670368725 | 308 | 5.6e-83 |
| AFD30954.1 | CrmA | BGC0000966 | Caerulomycin\_A\_biosynthetic\_gene\_cluster | 29 | 42.2034651266 | 308 | 5.6e-83 |
| CAN93347.1 | Polyketide\_synthase | BGC0000179 | Etnangien\_biosynthetic\_gene\_cluster | 35 | 23.8560639716 | 308 | 7.4e-83 |
| AHE80993.1 | PieA3 | BGC0001169 | Piericidin\_A1\_biosynthetic\_gene\_cluster | 32 | 31.4526876944 | 308 | 7.4e-83 |
| BAP05593.1 | CalE | BGC0000967 | Calyculin\_biosynthetic\_gene\_cluster | 35 | 23.678365171 | 308 | 7.4e-83 |
| AAS92537.1 | PKS1 | BGC0001044 | Sirodesmin\_biosynthetic\_gene\_cluster | 29 | 40.9595735229 | 307 | 9.6e-83 |
| AMH40443.1 | PKS | BGC0001350 | Phormidolide\_biosynthetic\_gene\_cluster | 35 | 26.0328742781 | 307 | 1.6e-82 |
| ALI92655.1 | CitS\_citrinin\_polyketide\_synthase | BGC0001338 | Citrinin\_biosynthetic\_gene\_cluster | 30 | 37.9386939138 | 307 | 1.6e-82 |
| AHB38498.1 | EpxE | BGC0000346 | Epoxomicin\_biosynthetic\_gene\_cluster | 30 | 39.0937361173 | 306 | 2.1e-82 |
| ACD39770.1 | non-reducing\_polyketide\_synthase | BGC0000134 | Radicicol\_biosynthetic\_gene\_cluster | 29 | 37.0501999112 | 306 | 2.1e-82 |
| ALD83688.1 | tAT\_polyketide\_synthase | BGC0001300 | Anthracimycin\_biosynthetic\_gene\_cluster | 35 | 23.4118169702 | 306 | 2.8e-82 |
| AAY32964.1 | DszA | BGC0001093 | Disorazole\_biosynthetic\_gene\_cluster | 35 | 26.8769435806 | 306 | 2.8e-82 |
| ABM63529.1 | BryX | BGC0000174 | Bryostatin\_biosynthetic\_gene\_cluster | 33 | 27.3656152821 | 306 | 2.8e-82 |
| AHV78247.1 | LasS2 | BGC0001245 | Lasiodiplodin\_biosynthetic\_gene\_cluster | 30 | 36.9169258108 | 305 | 3.7e-82 |
| CBK62733.1 |  | BGC0001115 | Thiomarinol\_biosynthetic\_gene\_cluster | 35 | 23.5895157708 | 305 | 4.8e-82 |
| CCA89328.1 | mixed\_trans-AT\_type\_I\_polyketide\_synthase/nonribosomal\_peptide\_synthetase | BGC0001111 | Rhizopodins\_biosynthetic\_gene\_cluster | 36 | 23.5006663705 | 305 | 6.2e-82 |
| CAO85898.1 | modular\_polyketide\_synthase\_NorC | BGC0000110 | Neoaureothin\_biosynthetic\_gene\_cluster | 32 | 29.320302088 | 305 | 6.2e-82 |
| AMH40422.1 | PKS | BGC0001350 | Phormidolide\_biosynthetic\_gene\_cluster | 34 | 23.32296757 | 304 | 8.2e-82 |
| CCT67991.1 | bikaverin\_cluster-polyketide\_synthase | BGC0000030 | Bikaverin\_biosynthetic\_gene\_cluster | 29 | 41.4482452243 | 304 | 8.2e-82 |
| ADN68477.1 | SorB | BGC0000184 | Sorangicin\_biosynthetic\_gene\_cluster | 37 | 23.678365171 | 304 | 8.2e-82 |
| AKQ22698.1 | malonyl\_CoA-acyl\_carrier\_protein\_transacylase | BGC0001186 | Misakinolide\_biosynthetic\_gene\_cluster | 36 | 23.678365171 | 304 | 8.2e-82 |
| EED18001.1 | polyketide\_synthase,\_putative | BGC0000154 | Stipitatic\_acid\_biosynthetic\_gene\_cluster | 28 | 41.5815193247 | 304 | 1.1e-81 |
| ABB90282.1 | polyketide\_synthase | BGC0001057 | Zearalenone\_biosynthetic\_gene\_cluster | 28 | 40.5597512217 | 304 | 1.1e-81 |
| AAY89052.1 | polyketide\_synthase | BGC0001069 | Chivosazole\_biosynthetic\_gene\_cluster | 37 | 23.3673922701 | 303 | 1.8e-81 |
| ADN68484.1 | SorI | BGC0000184 | Sorangicin\_biosynthetic\_gene\_cluster | 34 | 25.8996001777 | 303 | 1.8e-81 |
| ADN68476.1 | SorA | BGC0000184 | Sorangicin\_biosynthetic\_gene\_cluster | 35 | 23.7672145713 | 303 | 1.8e-81 |
| AGS06823.1 | type\_I\_polyketide\_synthase,\_PedI-like\_protein | BGC0001092 | Diaphorin\_biosynthetic\_gene\_cluster | 35 | 23.7227898712 | 303 | 1.8e-81 |
| CAQ34917.1 | polyketide\_synthase | BGC0000986 | Dkxanthene\_biosynthetic\_gene\_cluster | 32 | 29.4980008885 | 303 | 1.8e-81 |
| CAQ43079.1 | polyketide\_synthase | BGC0000970 | Chondrochloren\_biosynthetic\_gene\_cluster | 35 | 25.1443802754 | 303 | 2.4e-81 |
| ABI91469.1 | beta-ketoacyl\_synthase | BGC0001094 | Enacyloxin\_biosynthetic\_gene\_cluster | 33 | 29.2758773878 | 303 | 2.4e-81 |
| CBF70387.1 | polyketide\_synthase,\_putative\_(JCVI) | BGC0000684 | Asperthecin\_biosynthetic\_gene\_cluster | 28 | 42.7809862283 | 302 | 3.1e-81 |
| CAP95404.1 |  | BGC0001404 | Sorbicillin\_biosynthetic\_gene\_cluster | 28 | 41.3593958241 | 302 | 4e-81 |
| EAU29529.1 | hypothetical\_protein | BGC0000682 | Terretonin\_biosynthetic\_gene\_cluster | 34 | 25.011106175 | 302 | 4e-81 |
| CCA89326.1 | mixed\_trans-AT\_type\_I\_polyketide\_synthase/nonribosomal\_peptide\_synthetase | BGC0001111 | Rhizopodins\_biosynthetic\_gene\_cluster | 37 | 23.8116392714 | 302 | 5.3e-81 |
| ADD82941.1 | Bat3 | BGC0001099 | Kalimantacin\_/\_batumin\_biosynthetic\_gene\_cluster | 36 | 24.4780097734 | 301 | 6.9e-81 |
| AGC09485.1 | LobS2 | BGC0001183 | Lobophorin\_biosynthetic\_gene\_cluster | 36 | 23.6339404709 | 301 | 9e-81 |
| ADD82940.1 | Bat2 | BGC0001099 | Kalimantacin\_/\_batumin\_biosynthetic\_gene\_cluster | 36 | 24.4780097734 | 301 | 9e-81 |
| AAY89051.1 | hybrid\_nonribosomal\_peptide\_synthetase/polyketide\_synthase | BGC0001069 | Chivosazole\_biosynthetic\_gene\_cluster | 35 | 23.7672145713 | 300 | 1.2e-80 |
| ACH72912.1 | AflC | BGC0000011 | Aflatoxin/sterigmatocystin\_biosynthetic\_gene\_cluster | 28 | 39.8489560195 | 300 | 1.5e-80 |
| ADH01663.1 | putative\_polyketide\_synthase\_PKS3 | BGC0000099 | Monascorubrin\_biosynthetic\_gene\_cluster | 29 | 41.5370946246 | 300 | 1.5e-80 |
| CAE14178.1 |  | BGC0001180 | Isopropylstilbene\_biosynthetic\_gene\_cluster | 35 | 23.2341181697 | 300 | 1.5e-80 |
| ABS90470.1 | NRPS/PKS | BGC0001106 | Oxazolomycin\_biosynthetic\_gene\_cluster | 34 | 27.1434917814 | 300 | 2e-80 |
| CAJ76298.1 | putative\_hybrid\_polyketide-non-ribosomal\_peptide\_synthetase | BGC0000972 | Colibactin\_biosynthetic\_gene\_cluster | 30 | 37.6277210129 | 299 | 2.6e-80 |
| WP\_010639241.1 | beta-ketoacyl\_synthase | BGC0000958 | Antimycin\_biosynthetic\_gene\_cluster | 29 | 41.0484229231 | 299 | 2.6e-80 |
| AFR69333.1 | polyketide\_synthase\_SpiC1 | BGC0001045 | Spiruchostatin\_biosynthetic\_gene\_cluster | 35 | 23.011994669 | 299 | 2.6e-80 |
| CAL69891.1 | RhiD\_protein | BGC0001112 | Rhizoxins\_biosynthetic\_gene\_cluster | 34 | 25.1888049756 | 299 | 2.6e-80 |
| AAO65790.1 | ketosynthase-like\_protein | BGC0000100 | Monensin\_biosynthetic\_gene\_cluster | 32 | 30.653043092 | 299 | 2.6e-80 |
| AFV52200.1 | polyketide\_synthase\_module | BGC0000081 | Kedarcidin\_biosynthetic\_gene\_cluster | 29 | 42.0701910262 | 299 | 3.4e-80 |
| CAG23977.1 | polyketide\_synthase\_type\_I | BGC0000176 | Difficidin\_biosynthetic\_gene\_cluster | 34 | 24.9666814749 | 299 | 3.4e-80 |
| CCH26295.1 | putative\_polyketide\_synthase | BGC0001158\_c6 | Bikaverin\_biosynthetic\_gene\_cluster | 28 | 41.1816970235 | 299 | 4.5e-80 |
| AIJ04681.1 | polyketide\_synthase | BGC0001383 | Macrolactin\_biosynthetic\_gene\_cluster | 33 | 25.011106175 | 299 | 4.5e-80 |
| ABS90475.1 | PKS | BGC0001106 | Oxazolomycin\_biosynthetic\_gene\_cluster | 37 | 23.4118169702 | 298 | 5.8e-80 |
| CAJ57409.1 | polyketide\_synthase\_type\_I | BGC0000176 | Difficidin\_biosynthetic\_gene\_cluster | 35 | 23.3673922701 | 298 | 7.6e-80 |
| ADI59531.1 | CorI | BGC0001091 | Corallopyronin\_biosynthetic\_gene\_cluster | 34 | 25.9440248778 | 298 | 7.6e-80 |
| ADA82581.1 | trans-AT\_polyketide\_synthase | BGC0001110 | Psymberin\_biosynthetic\_gene\_cluster | 34 | 24.2558862728 | 298 | 7.6e-80 |
| ABC34832.1 | polyketide\_synthase | BGC0000186 | Thailandamide\_biosynthetic\_gene\_cluster | 34 | 23.4562416704 | 298 | 7.6e-80 |
| CAG23964.1 | polyketide\_synthase\_type\_I | BGC0000181 | Macrolactin\_biosynthetic\_gene\_cluster | 33 | 25.011106175 | 297 | 1e-79 |
| AGY62759.1 | EbeG | BGC0000051 | Ebelactone\_biosynthetic\_gene\_cluster | 34 | 23.2785428698 | 297 | 1.3e-79 |
| CBJ89760.1 | Polyketide\_synthase\_involved\_in\_xenocoumacin\_synthesis | BGC0001054 | Xenocoumacin\_biosynthetic\_gene\_cluster | 30 | 37.0501999112 | 297 | 1.7e-79 |
| CBW45749.1 | hybrid\_PKS/NRPS\_(PKSI) | BGC0000952\_c3 | Pristinamycin\_biosynthetic\_gene\_cluster | 35 | 23.4118169702 | 297 | 1.7e-79 |
| BAP05595.1 | CalG | BGC0000967 | Calyculin\_biosynthetic\_gene\_cluster | 37 | 22.345624167 | 296 | 2.2e-79 |
| ADN68483.1 | SorH | BGC0000184 | Sorangicin\_biosynthetic\_gene\_cluster | 35 | 23.9004886717 | 296 | 2.9e-79 |
| AAN85523.1 | polyketide\_synthase | BGC0001101 | Leinamycin\_biosynthetic\_gene\_cluster | 35 | 24.344735673 | 296 | 2.9e-79 |
| ACM79805.1 | ZmaA | BGC0001059 | Zwittermycin\_A\_biosynthetic\_gene\_cluster | 29 | 35.1399378054 | 295 | 3.8e-79 |
| ADY00130.1 | polyketide\_synthase | BGC0000104 | Mycophenolic\_acid\_biosynthetic\_gene\_cluster | 26 | 52.9098178587 | 295 | 3.8e-79 |
| ACY01401.1 | AT-less\_polyketide\_synthase | BGC0000083 | Lactimidomycin\_biosynthetic\_gene\_cluster | 35 | 23.8116392714 | 295 | 6.5e-79 |
| AAC49191.1 | putative\_polyketide\_synthase | BGC0000152 | Sterigmatocystin\_biosynthetic\_gene\_cluster | 27 | 40.6041759218 | 295 | 6.5e-79 |
| CAG23978.1 | polyketide\_synthase\_type\_I | BGC0000176 | Difficidin\_biosynthetic\_gene\_cluster | 34 | 24.0337627721 | 294 | 8.4e-79 |
| AFX60332.1 | polyketide\_synthase | BGC0001032 | Oocydin\_A\_biosynthetic\_gene\_cluster | 33 | 24.6112838738 | 294 | 1.1e-78 |
| ABC84465.1 | NigAX | BGC0000114 | Nigericin\_biosynthetic\_gene\_cluster | 31 | 30.0310972901 | 294 | 1.1e-78 |
| AAT69682.1 | polyketide\_synthase | BGC0001247 | Cercosporin\_biosynthetic\_gene\_cluster | 27 | 45.002221235 | 294 | 1.4e-78 |
| CTQ34883.1 | AtcF;\_polyketide\_synthase,\_modules\_8-10 | BGC0001301 | Anthracimycin\_biosynthetic\_gene\_cluster | 35 | 23.1452687694 | 294 | 1.4e-78 |
| ABX37384.1 | Beta-ketoacyl\_synthase | BGC0000984 | Delftibactin\_biosynthetic\_gene\_cluster | 31 | 34.9178143048 | 294 | 1.4e-78 |
| ADH01487.1 | polyketide\_synthase | BGC0000995 | FR901464\_biosynthetic\_gene\_cluster | 34 | 25.5442025766 | 293 | 1.9e-78 |
| AIC32693.1 | FR9DEF | BGC0001113 | Spliceostatin\_/\_FR901464\_biosynthetic\_gene\_cluster | 34 | 25.5442025766 | 293 | 1.9e-78 |
| CAN89635.1 | putative\_polyketide\_synthase | BGC0001070 | Kirromycin\_biosynthetic\_gene\_cluster | 37 | 21.7236783652 | 293 | 1.9e-78 |
| BAB69199.1 | modular\_polyketide\_synthase | BGC0000117 | Oligomycin\_biosynthetic\_gene\_cluster | 38 | 20.0355397601 | 293 | 2.5e-78 |
| ABF92489.1 | mixed\_type\_I\_polyketide\_synthase\_-\_peptide\_synthetase | BGC0001025 | Myxovirescin\_biosynthetic\_gene\_cluster | 36 | 23.011994669 | 293 | 2.5e-78 |
| AAY32965.1 | DszB | BGC0001093 | Disorazole\_biosynthetic\_gene\_cluster | 34 | 25.2776543758 | 293 | 2.5e-78 |
| ERM18797.1 | polyketide\_synthase | BGC0000172 | Basiliskamides\_biosynthetic\_gene\_cluster | 34 | 25.2776543758 | 293 | 2.5e-78 |
| CAL69889.1 | RhiB\_protein | BGC0001112 | Rhizoxins\_biosynthetic\_gene\_cluster | 32 | 27.6321634829 | 293 | 2.5e-78 |
| AGC95321.1 | CurS2 | BGC0000045 | Dehydrocurvularin\_biosynthetic\_gene\_cluster | 29 | 41.4038205242 | 292 | 3.2e-78 |
| ALD83704.1 | tAT\_polyketide\_synthase | BGC0001299 | Chlorotonil\_biosynthetic\_gene\_cluster | 36 | 22.0346512661 | 292 | 3.2e-78 |
| AHB38509.1 | EpnH | BGC0000345 | Eponemycin\_biosynthetic\_gene\_cluster | 29 | 40.337627721 | 292 | 4.2e-78 |
| CAG23957.2 | NRPS/PKS\_protein | BGC0001089 | Bacillaene\_biosynthetic\_gene\_cluster | 35 | 24.7001332741 | 292 | 4.2e-78 |
| CAN93348.1 | polyketide\_synthase | BGC0000179 | Etnangien\_biosynthetic\_gene\_cluster | 34 | 23.7227898712 | 292 | 5.5e-78 |
| AAM12913.2 | MmpD | BGC0000182 | Mupirocin\_biosynthetic\_gene\_cluster | 34 | 23.2341181697 | 291 | 9.3e-78 |
| ACR12418.1 | modular\_polyketide\_synthase,\_type\_I\_PKS | BGC0000185 | Tartrolon\_biosynthetic\_gene\_cluster | 33 | 25.011106175 | 290 | 1.2e-77 |
| ACY01402.1 | AT-less\_polyketide\_synthase | BGC0000083 | Lactimidomycin\_biosynthetic\_gene\_cluster | 34 | 23.8116392714 | 290 | 1.6e-77 |
| ADI59532.1 | CorJ | BGC0001091 | Corallopyronin\_biosynthetic\_gene\_cluster | 35 | 24.1226121724 | 290 | 2.1e-77 |
| CCA89327.1 | trans-AT\_type\_I\_polyketide\_synthase | BGC0001111 | Rhizopodins\_biosynthetic\_gene\_cluster | 34 | 23.678365171 | 289 | 2.7e-77 |
| AEC04363.1 | polyketide\_synthase | BGC0000178 | Elansolid\_biosynthetic\_gene\_cluster | 36 | 23.011994669 | 289 | 2.7e-77 |
| ADN68478.1 | SorC | BGC0000184 | Sorangicin\_biosynthetic\_gene\_cluster | 33 | 25.4997778765 | 289 | 2.7e-77 |
| CCC21123.1 | type-I\_polyketide\_synthases | BGC0000171 | 9-methylstreptimidone\_biosynthetic\_gene\_cluster | 34 | 23.4118169702 | 289 | 3.5e-77 |
| BAC76471.1 | type\_I\_polyketide\_synthase\_LkcF | BGC0001100 | Lankacidin\_biosynthetic\_gene\_cluster | 35 | 23.4118169702 | 289 | 4.6e-77 |
| AFX60313.1 | polyketide\_synthase | BGC0001031 | Oocydin\_A\_biosynthetic\_gene\_cluster | 35 | 23.8116392714 | 289 | 4.6e-77 |
| AEH42473.1 | polyketide\_synthase | BGC0000032 | Calcimycin\_biosynthetic\_gene\_cluster | 33 | 29.8533984896 | 289 | 4.6e-77 |
| ABC36687.1 | polyketide\_synthase | BGC0000964 | Burkholdac\_biosynthetic\_gene\_cluster | 35 | 23.2785428698 | 288 | 6e-77 |
| CAG23960.2 | hybrid\_NRPS/PKS | BGC0001089 | Bacillaene\_biosynthetic\_gene\_cluster | 34 | 23.5006663705 | 288 | 7.9e-77 |
| ACR50796.1 | putative\_polyketide\_synthase | BGC0000163 | Tetronasin\_biosynthetic\_gene\_cluster | 35 | 24.1226121724 | 288 | 7.9e-77 |
| CAE02606.1 | polyketide\_synthase\_type\_I | BGC0000024 | Aureothin\_biosynthetic\_gene\_cluster | 30 | 29.8089737894 | 287 | 1e-76 |
| AFX60311.1 | polyketide\_synthase | BGC0001031 | Oocydin\_A\_biosynthetic\_gene\_cluster | 34 | 25.9440248778 | 287 | 1e-76 |
| AGN74894.1 | nonribosomal\_peptide\_synthetase/polyketide\_synthase\_hybrid\_protein | BGC0000459 | Griseoviridin\_/\_viridogrisein\_biosynthetic\_gene\_cluster | 37 | 24.3891603732 | 287 | 1.3e-76 |
| AFO59866.1 | ChxE | BGC0000175 | Cycloheximide\_/\_actiphenol\_biosynthetic\_gene\_cluster | 34 | 24.7445579742 | 287 | 1.3e-76 |
| AKA59448.1 | polyketide\_synthase | BGC0001203 | Clarexpoxcin\_biosynthetic\_gene\_cluster | 29 | 36.9169258108 | 287 | 1.8e-76 |
| CBW45748.1 | hybrid\_PKS/NRPS\_(PKSI) | BGC0000952\_c3 | Pristinamycin\_biosynthetic\_gene\_cluster | 34 | 23.7227898712 | 287 | 1.8e-76 |
| AEC04357.1 | polyketide\_synthase | BGC0000178 | Elansolid\_biosynthetic\_gene\_cluster | 34 | 24.4780097734 | 287 | 1.8e-76 |
| CAN93351.1 | polyketide\_synthase | BGC0000179 | Etnangien\_biosynthetic\_gene\_cluster | 35 | 23.4118169702 | 287 | 1.8e-76 |
| ctg1\_orf8 |  | BGC0001109 | Pederin\_biosynthetic\_gene\_cluster | 35 | 21.8569524656 | 286 | 2.3e-76 |
| BAE93740.1 | type\_I\_polyketide\_synthase-related\_protein | BGC0000164 | Tetronomycin\_biosynthetic\_gene\_cluster | 35 | 27.0990670813 | 286 | 2.3e-76 |
| AAS47562.1 | mixed\_type\_I\_polyketide\_synthase\_-\_peptide\_synthetase | BGC0001108 | Pederin\_biosynthetic\_gene\_cluster | 35 | 21.8569524656 | 286 | 2.3e-76 |
| CAG23965.1 | polyketide\_synthase\_type\_I | BGC0000181 | Macrolactin\_biosynthetic\_gene\_cluster | 33 | 23.4562416704 | 286 | 2.3e-76 |
| ABF85931.1 | non-ribosomal\_peptide\_synthase/polyketide\_synthase\_Ta1 | BGC0001025 | Myxovirescin\_biosynthetic\_gene\_cluster | 34 | 24.2558862728 | 286 | 3e-76 |
| CAG23959.2 | polyketide\_synthase | BGC0001089 | Bacillaene\_biosynthetic\_gene\_cluster | 33 | 24.8334073745 | 286 | 3e-76 |
| ABI91464.1 | beta-ketoacyl\_synthase | BGC0001094 | Enacyloxin\_biosynthetic\_gene\_cluster | 32 | 29.6312749889 | 285 | 3.9e-76 |
| AIJ04680.1 | polyketide\_synthase | BGC0001383 | Macrolactin\_biosynthetic\_gene\_cluster | 32 | 23.4562416704 | 285 | 3.9e-76 |
| AFX60309.1 | polyketide\_synthase | BGC0001031 | Oocydin\_A\_biosynthetic\_gene\_cluster | 31 | 25.8107507774 | 285 | 5.1e-76 |
| ALD83687.1 | tAT\_polyketide\_synthase | BGC0001300 | Anthracimycin\_biosynthetic\_gene\_cluster | 35 | 23.9449133718 | 285 | 5.1e-76 |
| CAL69890.1 | RhiC\_protein | BGC0001112 | Rhizoxins\_biosynthetic\_gene\_cluster | 32 | 23.2785428698 | 285 | 6.7e-76 |
| ACR13997.1 | modular\_polyketide\_synthase,\_type\_I\_PKS | BGC0000185 | Tartrolon\_biosynthetic\_gene\_cluster | 35 | 23.5450910706 | 285 | 6.7e-76 |
| ABP57747.1 | DepC | BGC0000993 | FK228\_biosynthetic\_gene\_cluster | 34 | 23.0564193692 | 285 | 6.7e-76 |
| AGN71604.1 | conidial\_yellow\_pigment\_biosynthesis\_polyketide\_synthase | BGC0000027 | Azaphilone\_biosynthetic\_gene\_cluster | 27 | 41.4038205242 | 284 | 1.1e-75 |
| AFX60336.1 | polyketide\_synthase | BGC0001032 | Oocydin\_A\_biosynthetic\_gene\_cluster | 35 | 23.8116392714 | 284 | 1.1e-75 |
| ACY01391.1 | AT-less\_polyketide\_synthase | BGC0000177 | Dorrigocin\_/\_migrastatin\_biosynthetic\_gene\_cluster | 33 | 23.989338072 | 284 | 1.1e-75 |
| ABC33986.1 | polyketide\_synthase,\_putative | BGC0000186 | Thailandamide\_biosynthetic\_gene\_cluster | 36 | 23.1452687694 | 284 | 1.5e-75 |
| CTQ34882.1 | AtcE;\_polyketide\_synthase,\_modules\_5-7 | BGC0001301 | Anthracimycin\_biosynthetic\_gene\_cluster | 33 | 24.2558862728 | 284 | 1.5e-75 |
| CBW45741.1 | hybrid\_PKS/NRPS\_(PKSI) | BGC0000952\_c3 | Pristinamycin\_biosynthetic\_gene\_cluster | 35 | 23.2785428698 | 284 | 1.5e-75 |
| CBJ89764.1 | Polyketide\_synthase\_involved\_in\_xenocoumacin\_synthesis | BGC0001054 | Xenocoumacin\_biosynthetic\_gene\_cluster | 30 | 36.8725011106 | 284 | 1.5e-75 |
| AKA59437.1 | polyketide\_synthase | BGC0001202 | Landepoxcin\_biosynthetic\_gene\_cluster | 30 | 33.5406486006 | 283 | 1.9e-75 |
| AFX60334.1 | polyketide\_synthase | BGC0001032 | Oocydin\_A\_biosynthetic\_gene\_cluster | 35 | 24.1670368725 | 283 | 1.9e-75 |
| ADI59533.1 | CorK | BGC0001091 | Corallopyronin\_biosynthetic\_gene\_cluster | 33 | 23.989338072 | 283 | 2.5e-75 |
| ACY01400.1 | AT-less\_polyketide\_synthase | BGC0000083 | Lactimidomycin\_biosynthetic\_gene\_cluster | 32 | 24.3003109729 | 283 | 2.5e-75 |
| AFN27480.1 | pks\_BonA | BGC0000173 | Bongkrekic\_acid\_biosynthetic\_gene\_cluster | 34 | 26.2549977788 | 283 | 2.5e-75 |
| ACY01390.1 | AT-less\_polyketide\_synthase | BGC0000177 | Dorrigocin\_/\_migrastatin\_biosynthetic\_gene\_cluster | 34 | 21.9458018658 | 283 | 2.5e-75 |
| AKQ22696.1 | malonyl\_CoA-acyl\_carrier\_protein\_transacylase | BGC0001186 | Misakinolide\_biosynthetic\_gene\_cluster | 34 | 25.2332296757 | 282 | 3.3e-75 |
| AGS06887.1 | mixed\_type\_I\_polyketide\_synthase/non-ribosomal\_peptide\_synthetase,\_PedF-like\_protein | BGC0001092\_c2 | Diaphorin\_biosynthetic\_gene\_cluster | 34 | 23.5006663705 | 282 | 3.3e-75 |
| CCA89329.1 | trans-AT\_type\_I\_polyketide\_synthase | BGC0001111 | Rhizopodins\_biosynthetic\_gene\_cluster | 36 | 23.4562416704 | 282 | 4.3e-75 |
| CBK62731.1 |  | BGC0001115 | Thiomarinol\_biosynthetic\_gene\_cluster | 34 | 24.5224344736 | 282 | 4.3e-75 |
| CBK62724.1 |  | BGC0001115 | Thiomarinol\_biosynthetic\_gene\_cluster | 32 | 23.7672145713 | 282 | 5.7e-75 |
| AFK83819.1 | PtzC | BGC0001107 | Patellazole\_biosynthetic\_gene\_cluster | 36 | 21.8569524656 | 282 | 5.7e-75 |
| ACY01392.1 | AT-less\_polyketide\_synthase | BGC0000177 | Dorrigocin\_/\_migrastatin\_biosynthetic\_gene\_cluster | 33 | 23.5450910706 | 282 | 5.7e-75 |
| CAN89631.1 | putative\_polyketide\_synthase | BGC0001070 | Kirromycin\_biosynthetic\_gene\_cluster | 34 | 23.4118169702 | 282 | 5.7e-75 |
| AGS06824.1 | mixed\_type\_I\_polyketide\_synthase/non-ribosomal\_peptide\_synthetase,\_PedH-like\_protein | BGC0001092 | Diaphorin\_biosynthetic\_gene\_cluster | 34 | 21.9458018658 | 281 | 7.4e-75 |
| AEC04362.1 | polyketide\_synthase | BGC0000178 | Elansolid\_biosynthetic\_gene\_cluster | 34 | 23.5895157708 | 281 | 9.7e-75 |
| AEC04361.1 | polyketide\_synthase | BGC0000178 | Elansolid\_biosynthetic\_gene\_cluster | 34 | 24.0781874722 | 281 | 9.7e-75 |
| BAF50722.1 | polyketide\_synthase | BGC0001116 | Virginiamycin\_biosynthetic\_gene\_cluster | 36 | 21.7236783652 | 281 | 9.7e-75 |
| AAO65798.1 | monensin\_polyketide\_synthase\_modules\_3\_and\_4 | BGC0000100 | Monensin\_biosynthetic\_gene\_cluster | 32 | 27.4988893825 | 281 | 9.7e-75 |
| AGN11881.1 | TstDEF | BGC0001114 | Thailanstatin\_biosynthetic\_gene\_cluster | 35 | 23.32296757 | 281 | 9.7e-75 |
| ADN68482.1 | SorG | BGC0000184 | Sorangicin\_biosynthetic\_gene\_cluster | 34 | 25.6330519769 | 280 | 1.3e-74 |
| AEF33079.1 | polyketide\_synthase | BGC0001039 | Pyridomycin\_biosynthetic\_gene\_cluster | 29 | 37.3611728121 | 280 | 2.2e-74 |
| ERM18798.1 | polyketide\_synthase | BGC0000172 | Basiliskamides\_biosynthetic\_gene\_cluster | 33 | 25.8996001777 | 280 | 2.2e-74 |
| BAP05591.1 | CalC | BGC0000967 | Calyculin\_biosynthetic\_gene\_cluster | 35 | 23.011994669 | 279 | 4.8e-74 |
| CBW45692.1 | putative\_modular\_polyketide\_synthase\_(fragment) | BGC0000952\_c3 | Pristinamycin\_biosynthetic\_gene\_cluster | 34 | 23.4562416704 | 278 | 6.3e-74 |
| ACR33077.1 | polyketide\_synthase | BGC0000017 | Anatoxin\_biosynthetic\_gene\_cluster | 31 | 28.6983562861 | 278 | 8.2e-74 |
| AFL48521.1 | beta-ketosynthase | BGC0000084 | Laidlomycin\_biosynthetic\_gene\_cluster | 30 | 29.9422478898 | 278 | 8.2e-74 |
| AAF08795.1 | MycA | BGC0001103 | Mycosubtilin\_biosynthetic\_gene\_cluster | 33 | 23.32296757 | 278 | 8.2e-74 |
| BAC76474.1 | type\_I\_polyketide\_synthase\_LkcC | BGC0001100 | Lankacidin\_biosynthetic\_gene\_cluster | 34 | 23.678365171 | 278 | 8.2e-74 |
| CAG23967.1 | polyketide\_synthase\_type\_I | BGC0000181 | Macrolactin\_biosynthetic\_gene\_cluster | 33 | 23.4562416704 | 278 | 8.2e-74 |
| AAF26923.1 | polyketide\_synthase | BGC0000988 | Epothilone\_biosynthetic\_gene\_cluster | 28 | 36.0728565082 | 277 | 1.1e-73 |
| ADN68480.1 | SorE | BGC0000184 | Sorangicin\_biosynthetic\_gene\_cluster | 33 | 24.344735673 | 277 | 1.4e-73 |
| AIJ04684.1 | polyketide\_synthase | BGC0001383 | Macrolactin\_biosynthetic\_gene\_cluster | 33 | 23.4562416704 | 277 | 1.4e-73 |
| ABM63528.1 | BryC | BGC0000174 | Bryostatin\_biosynthetic\_gene\_cluster | 34 | 24.3003109729 | 276 | 2.4e-73 |
| AAM12909.2 | MmpA | BGC0000182 | Mupirocin\_biosynthetic\_gene\_cluster | 35 | 24.2558862728 | 276 | 2.4e-73 |
| ADA82585.1 | hybrid\_trans-AT\_polyketide\_synthase\_-\_nonribosomal\_peptide\_synthetase | BGC0001110 | Psymberin\_biosynthetic\_gene\_cluster | 35 | 23.5006663705 | 276 | 3.1e-73 |
| ADA69239.2 | trans-AT\_hybrid\_polyketide\_synthase-NRPS | BGC0001071 | Nosperin\_biosynthetic\_gene\_cluster | 34 | 23.678365171 | 276 | 3.1e-73 |
| ADN68479.1 | SorD | BGC0000184 | Sorangicin\_biosynthetic\_gene\_cluster | 35 | 23.1896934696 | 276 | 3.1e-73 |
| ACB46197.1 | polyketide\_synthase | BGC0000989 | Epothilone\_biosynthetic\_gene\_cluster | 28 | 36.0728565082 | 275 | 4.1e-73 |
| CAL69888.1 | RhiA\_protein | BGC0001112 | Rhizoxins\_biosynthetic\_gene\_cluster | 36 | 23.4562416704 | 275 | 5.3e-73 |
| AGN11882.1 | TstGH | BGC0001114 | Thailanstatin\_biosynthetic\_gene\_cluster | 34 | 23.32296757 | 275 | 6.9e-73 |
| AFK83820.1 | PtzD | BGC0001107\_c2 | Patellazole\_biosynthetic\_gene\_cluster | 33 | 23.989338072 | 274 | 9e-73 |
| AAV97877.1 | OnnI | BGC0001105 | Onnamide\_biosynthetic\_gene\_cluster | 34 | 24.4780097734 | 274 | 1.2e-72 |
| CAJ57410.1 | polyketide\_synthase\_type\_I | BGC0000176 | Difficidin\_biosynthetic\_gene\_cluster | 35 | 23.9004886717 | 274 | 1.2e-72 |
| AAM12911.1 | MmpB | BGC0000182 | Mupirocin\_biosynthetic\_gene\_cluster | 34 | 23.5895157708 | 274 | 1.5e-72 |
| AAP42872.1 | NanA9 | BGC0000105 | Nanchangmycin\_biosynthetic\_gene\_cluster | 31 | 29.7201243892 | 274 | 1.5e-72 |
| AIJ04686.1 | polyketide\_synthase | BGC0001383 | Macrolactin\_biosynthetic\_gene\_cluster | 32 | 23.678365171 | 273 | 2e-72 |
| AGN74893.1 | nonribosomal\_peptide\_synthetase/polyketide\_synthase\_hybrid\_protein | BGC0000459 | Griseoviridin\_/\_viridogrisein\_biosynthetic\_gene\_cluster | 33 | 24.7445579742 | 273 | 2e-72 |
| CAL69893.1 | RhiE\_protein | BGC0001112 | Rhizoxins\_biosynthetic\_gene\_cluster | 33 | 23.3673922701 | 273 | 2e-72 |
| AAN85522.1 | hybrid\_nonribosomal\_peptide\_synthetase\_/\_polyketide\_synthase | BGC0001101 | Leinamycin\_biosynthetic\_gene\_cluster | 36 | 23.5895157708 | 272 | 3.4e-72 |
| AMH40423.1 | PKS | BGC0001350 | Phormidolide\_biosynthetic\_gene\_cluster | 34 | 24.2114615726 | 272 | 3.4e-72 |
| CAG23968.1 | polyketide\_synthase\_type\_I | BGC0000181 | Macrolactin\_biosynthetic\_gene\_cluster | 32 | 23.678365171 | 272 | 4.5e-72 |
| ABC35522.1 | thiotemplate\_mechanism\_natural\_product\_synthetase | BGC0000186 | Thailandamide\_biosynthetic\_gene\_cluster | 33 | 24.1226121724 | 271 | 7.7e-72 |
| ABM63537.1 | BryA | BGC0000174 | Bryostatin\_biosynthetic\_gene\_cluster | 35 | 24.2558862728 | 270 | 1.3e-71 |
| ABC34154.1 | thiotemplate\_mechanism\_natural\_product\_synthetase | BGC0000961 | Bactobolin\_biosynthetic\_gene\_cluster | 34 | 23.6339404709 | 270 | 1.3e-71 |
| AFX60318.1 | polyketide\_synthase | BGC0001031 | Oocydin\_A\_biosynthetic\_gene\_cluster | 33 | 24.2558862728 | 270 | 2.2e-71 |
| ABM63527.1 | BryB | BGC0000174 | Bryostatin\_biosynthetic\_gene\_cluster | 35 | 23.6339404709 | 269 | 2.9e-71 |
| AFN27482.1 | pks\_BonC | BGC0000173 | Bongkrekic\_acid\_biosynthetic\_gene\_cluster | 33 | 24.4335850733 | 269 | 2.9e-71 |
| CAN93349.1 | polyketide\_synthase | BGC0000179 | Etnangien\_biosynthetic\_gene\_cluster | 34 | 23.678365171 | 269 | 3.8e-71 |
| ABC34176.1 | polyketide\_synthase,\_putative | BGC0000186 | Thailandamide\_biosynthetic\_gene\_cluster | 33 | 24.8334073745 | 269 | 3.8e-71 |
| AFX60341.1 | polyketide\_synthase | BGC0001032 | Oocydin\_A\_biosynthetic\_gene\_cluster | 31 | 32.2967569969 | 269 | 3.8e-71 |
| ADH01489.1 | type\_I\_polyketide\_synthase | BGC0000995 | FR901464\_biosynthetic\_gene\_cluster | 33 | 23.32296757 | 269 | 5e-71 |
| AIC32694.1 | FR9GH | BGC0001113 | Spliceostatin\_/\_FR901464\_biosynthetic\_gene\_cluster | 33 | 23.32296757 | 269 | 5e-71 |
| AFN27483.1 | pks\_BonD | BGC0000173 | Bongkrekic\_acid\_biosynthetic\_gene\_cluster | 35 | 23.5006663705 | 268 | 6.5e-71 |
| ADB12493.1 | EpoF | BGC0000990 | Epothilone\_biosynthetic\_gene\_cluster | 31 | 28.342958685 | 268 | 8.5e-71 |
| BAB69698.1 | iturin\_A\_synthetase\_A | BGC0001098 | Iturin\_biosynthetic\_gene\_cluster | 33 | 23.2785428698 | 268 | 8.5e-71 |
| CAJ57408.1 | polyketide\_synthase\_type\_I | BGC0000176 | Difficidin\_biosynthetic\_gene\_cluster | 35 | 21.2794313638 | 268 | 8.5e-71 |
| CAE52339.1 | Polyketide\_non-ribosomal\_peptide\_synthase | BGC0001088 | Albicidin\_biosynthetic\_gene\_cluster | 31 | 23.3673922701 | 267 | 1.1e-70 |
| ABS74181.1 | BmyA | BGC0001090 | Bacillomycin\_biosynthetic\_gene\_cluster | 33 | 23.2785428698 | 267 | 1.4e-70 |
| AEC04356.1 | polyketide\_synthase | BGC0000178 | Elansolid\_biosynthetic\_gene\_cluster | 33 | 22.9675699689 | 266 | 2.5e-70 |
| ABB86408.1 | GelA | BGC0000067 | Geldanamycin\_biosynthetic\_gene\_cluster | 30 | 30.8307418925 | 266 | 2.5e-70 |
| ABC34675.1 | polyketide\_synthase,\_putative | BGC0000186 | Thailandamide\_biosynthetic\_gene\_cluster | 32 | 23.9004886717 | 266 | 3.2e-70 |
| CAJ57411.1 | polyketide\_synthase\_type\_I | BGC0000176 | Difficidin\_biosynthetic\_gene\_cluster | 32 | 23.3673922701 | 265 | 4.2e-70 |
| CAE45671.1 | borrelidin\_polyketide\_synthase,\_type\_I | BGC0000031 | Borrelidin\_biosynthetic\_gene\_cluster | 35 | 23.5006663705 | 265 | 4.2e-70 |
| AFN27481.1 | pks\_BonB | BGC0000173 | Bongkrekic\_acid\_biosynthetic\_gene\_cluster | 32 | 24.6112838738 | 265 | 4.2e-70 |
| AHD05679.1 | putative\_non-ribosomal\_peptide\_ligase/\_polyketide\_synthase\_hybrid | BGC0000402 | Paenilarvins\_biosynthetic\_gene\_cluster | 33 | 23.5006663705 | 265 | 5.5e-70 |
| CAN93352.1 | polyketide\_synthase | BGC0000179 | Etnangien\_biosynthetic\_gene\_cluster | 33 | 24.0781874722 | 264 | 9.4e-70 |
| EAA65602.1 | hypothetical\_protein | BGC0000022 | Asperfuranone\_biosynthetic\_gene\_cluster | 27 | 42.6921368281 | 264 | 1.2e-69 |
| ACG60781.1 | PKS(KS/AT/MT/KR/ACP) | BGC0001058 | Zorbamycin\_biosynthetic\_gene\_cluster | 32 | 30.4309195913 | 264 | 1.6e-69 |
| AAO06916.1 | GdmAI | BGC0000066 | Geldanamycin\_biosynthetic\_gene\_cluster | 29 | 30.8307418925 | 264 | 1.6e-69 |
| CAE46850.1 | Type\_I\_modular\_polyketide\_synthase | BGC0000103 | Mycolactone\_biosynthetic\_gene\_cluster | 32 | 30.4753442914 | 263 | 2.1e-69 |
| ABC35796.1 | polyketide\_synthase,\_putative | BGC0001102 | Malleilactone\_biosynthetic\_gene\_cluster | 32 | 23.011994669 | 262 | 3.6e-69 |
| AAY32966.1 | DszC | BGC0001093 | Disorazole\_biosynthetic\_gene\_cluster | 34 | 24.1670368725 | 262 | 4.6e-69 |
| CBJ89766.1 | Polyketide\_synthase\_involved\_in\_xenocoumacin\_synthesis | BGC0001054 | Xenocoumacin\_biosynthetic\_gene\_cluster | 27 | 37.9386939138 | 261 | 7.9e-69 |
| AKQ22697.1 | malonyl\_CoA-acyl\_carrier\_protein\_transacylase | BGC0001186 | Misakinolide\_biosynthetic\_gene\_cluster | 33 | 24.1670368725 | 260 | 1.8e-68 |
| ABM63530.1 | BryD | BGC0000174 | Bryostatin\_biosynthetic\_gene\_cluster | 34 | 24.3891603732 | 258 | 6.7e-68 |
| AAY28225.1 | HbmAI | BGC0000074 | Herbimycin\_biosynthetic\_gene\_cluster | 30 | 30.6974677921 | 258 | 8.8e-68 |
| CAG23983.1 | polyketide\_synthase\_type\_I | BGC0000176 | Difficidin\_biosynthetic\_gene\_cluster | 33 | 23.1452687694 | 257 | 1.5e-67 |
| ADB23403.1 | polyketide\_synthase\_type\_I | BGC0001062 | TLN-05220\_biosynthetic\_gene\_cluster | 32 | 27.9431363838 | 255 | 4.3e-67 |
| ABC38101.1 | polyketide\_synthase | BGC0000964 | Burkholdac\_biosynthetic\_gene\_cluster | 31 | 24.7001332741 | 255 | 4.3e-67 |
| ctg1\_orf255 |  | BGC0001200 | Lorneic\_acid\_A\_biosynthetic\_gene\_cluster | 31 | 30.4309195913 | 255 | 7.4e-67 |
| AAG23265.1 | polyketide\_synthase\_extender\_module\_2 | BGC0000148 | Spinosad\_biosynthetic\_gene\_cluster | 30 | 31.5415370946 | 254 | 1.3e-66 |
| CUI25673.1 | Thiolactomycin\_Polyketide\_synthase | BGC0001353 | Thiolactomycin\_biosynthetic\_gene\_cluster | 33 | 23.3673922701 | 254 | 1.3e-66 |
| ABS90471.1 | PKS\_type\_I | BGC0001106 | Oxazolomycin\_biosynthetic\_gene\_cluster | 34 | 22.2567747668 | 253 | 1.7e-66 |
| AFL48529.1 | laidlomycin\_polyketide\_synthase\_(module\_5\_and\_module\_6) | BGC0000084 | Laidlomycin\_biosynthetic\_gene\_cluster | 29 | 35.8507330076 | 252 | 3.7e-66 |
| ABV83230.1 | CppC | BGC0000116 | Nystatin-like\_Pseudonocardia\_polyene\_biosynthetic\_gene\_cluster | 31 | 29.98667259 | 252 | 6.3e-66 |
| ALV82446.1 | putative\_type\_I\_polyketide\_synthase | BGC0001382 | Svaricin\_biosynthetic\_gene\_cluster | 34 | 23.2785428698 | 251 | 8.2e-66 |
| ABC84459.1 | NigAIV | BGC0000114 | Nigericin\_biosynthetic\_gene\_cluster | 31 | 29.8533984896 | 251 | 8.2e-66 |
| AAZ77694.1 | ChlA2 | BGC0000036 | Chlorothricin\_biosynthetic\_gene\_cluster | 32 | 25.4109284762 | 251 | 8.2e-66 |
| ABF88102.1 | polyketide\_synthase | BGC0001025 | Myxovirescin\_biosynthetic\_gene\_cluster | 33 | 23.32296757 | 251 | 8.2e-66 |
| ADC45538.1 | modular\_polyketide\_synthase | BGC0000093 | Meilingmycin\_biosynthetic\_gene\_cluster | 31 | 29.7201243892 | 251 | 8.2e-66 |
| BAF50721.1 | hybrid\_non\_ribosomal\_peptide\_synthetase-polyketide\_synthase | BGC0001116 | Virginiamycin\_biosynthetic\_gene\_cluster | 35 | 23.8116392714 | 251 | 1.1e-65 |
| AAD03047.1 | type\_I\_polyketide\_synthase | BGC0000041 | Coronafacic\_acid\_biosynthetic\_gene\_cluster | 31 | 33.5850733008 | 251 | 1.1e-65 |
| BAP05597.1 | CalI | BGC0000967 | Calyculin\_biosynthetic\_gene\_cluster | 34 | 21.1905819636 | 251 | 1.1e-65 |
| ABK32260.1 | AmbF | BGC0000014 | Ambruticin\_biosynthetic\_gene\_cluster | 32 | 23.8560639716 | 250 | 1.8e-65 |
| BAP05590.1 | CalB | BGC0000967 | Calyculin\_biosynthetic\_gene\_cluster | 33 | 23.3673922701 | 249 | 3.1e-65 |
| BAQ25482.1 | type\_I\_polyketide\_synthase | BGC0001288 | Maklamicin\_biosynthetic\_gene\_cluster | 30 | 31.2749888938 | 249 | 3.1e-65 |
| ABW96541.1 | type\_I\_modular\_polyketide\_synthase | BGC0000159 | Tautomycin\_biosynthetic\_gene\_cluster | 30 | 31.2305641937 | 248 | 5.3e-65 |
| ADH01488.1 | polyketide\_synthase | BGC0000995 | FR901464\_biosynthetic\_gene\_cluster | 33 | 24.4780097734 | 248 | 9.1e-65 |
| AAO65799.1 | monensin\_polyketide\_synthase\_modules\_5\_and\_6 | BGC0000100 | Monensin\_biosynthetic\_gene\_cluster | 28 | 34.4291426033 | 248 | 9.1e-65 |
| AAP42873.1 | NanA11 | BGC0000105 | Nanchangmycin\_biosynthetic\_gene\_cluster | 29 | 36.2505553088 | 247 | 1.2e-64 |
| ABI94380.1 | tautomycetin\_biosynthetic\_PKS | BGC0000157 | Tautomycetin\_biosynthetic\_gene\_cluster | 28 | 32.2079075966 | 245 | 4.5e-64 |
| ACN69991.1 | polyketide\_synthase | BGC0000079 | Indanomycin\_biosynthetic\_gene\_cluster | 30 | 29.8089737894 | 245 | 4.5e-64 |
| AHI59107.1 | locillomycin\_synthase\_D | BGC0001005 | Locillomycin\_biosynthetic\_gene\_cluster | 32 | 23.6339404709 | 245 | 4.5e-64 |
| ABV91287.1 | type\_I\_modular\_polyketide\_synthase | BGC0000158 | Tautomycetin\_biosynthetic\_gene\_cluster | 29 | 30.7863171924 | 245 | 5.9e-64 |
| BAQ25513.1 | type\_I\_polyketide\_synthase | BGC0001288 | Maklamicin\_biosynthetic\_gene\_cluster | 30 | 31.0528653932 | 245 | 7.7e-64 |
| CAG23961.2 | polyketide\_synthase | BGC0001089 | Bacillaene\_biosynthetic\_gene\_cluster | 31 | 26.2549977788 | 244 | 1.3e-63 |
| CAE02605.1 | polyketide\_synthase\_type\_I | BGC0000024 | Aureothin\_biosynthetic\_gene\_cluster | 31 | 27.676588183 | 243 | 1.7e-63 |
| CUW01173.1 | Type\_I\_polyketide\_synthase | BGC0001359 | PM100117\_/\_PM100118\_biosynthetic\_gene\_cluster | 29 | 31.3638382941 | 243 | 1.7e-63 |
| CAD15512.1 | probable\_polyketide\_synthase\_protein | BGC0001014 | Micacocidin\_biosynthetic\_gene\_cluster | 33 | 24.0337627721 | 243 | 2.2e-63 |
| AEC04364.1 | polyketide\_synthase | BGC0000178 | Elansolid\_biosynthetic\_gene\_cluster | 32 | 23.2341181697 | 243 | 2.9e-63 |
| AGZ15474.1 | putative\_type\_I\_polyketide\_synthase | BGC0001036 | Polyoxypeptin\_biosynthetic\_gene\_cluster | 35 | 22.4344735673 | 243 | 2.9e-63 |
| CBF79143.1 | polyketide\_synthase,\_putative\_(JCVI) | BGC0000013 | Alternariol\_biosynthetic\_gene\_cluster | 29 | 26.4326965793 | 241 | 8.5e-63 |
| AFL48528.1 | laidlomycin\_polyketide\_synthase\_(module\_7\_and\_module\_8) | BGC0000084 | Laidlomycin\_biosynthetic\_gene\_cluster | 33 | 22.1679253665 | 240 | 1.9e-62 |
| AHH99921.1 | PKS\_I | BGC0000002 | Aculeximycin\_biosynthetic\_gene\_cluster | 29 | 29.6312749889 | 239 | 3.2e-62 |
| BAP05594.1 | CalF | BGC0000967 | Calyculin\_biosynthetic\_gene\_cluster | 30 | 23.4562416704 | 238 | 5.5e-62 |
| CAL69894.1 | RhiF\_protein | BGC0001112 | Rhizoxins\_biosynthetic\_gene\_cluster | 29 | 27.7210128832 | 237 | 1.2e-61 |
| ABS90472.1 | PKS | BGC0001106 | Oxazolomycin\_biosynthetic\_gene\_cluster | 31 | 25.3665037761 | 237 | 2.1e-61 |
| AFL48526.1 | laidlomycin\_polyketide\_synthase\_(module\_2) | BGC0000084 | Laidlomycin\_biosynthetic\_gene\_cluster | 28 | 32.2079075966 | 235 | 4.7e-61 |
| CUW01174.1 | Type\_I\_polyketide\_synthase | BGC0001359 | PM100117\_/\_PM100118\_biosynthetic\_gene\_cluster | 27 | 36.4726788094 | 234 | 1.4e-60 |
| BAP05596.1 | CalH | BGC0000967 | Calyculin\_biosynthetic\_gene\_cluster | 32 | 23.5006663705 | 233 | 1.8e-60 |
| ABB88521.1 | polyketide\_synthase\_type\_I | BGC0000050 | E-837\_biosynthetic\_gene\_cluster | 32 | 23.2785428698 | 233 | 3e-60 |
| ADH01485.1 | putative\_mixed\_polyketide\_synthase/non-ribosomal\_peptide\_synthetase | BGC0000995 | FR901464\_biosynthetic\_gene\_cluster | 34 | 25.9440248778 | 232 | 3.9e-60 |
| BAC76470.1 | type\_I\_polyketide\_synthase\_LkcG | BGC0001100 | Lankacidin\_biosynthetic\_gene\_cluster | 32 | 23.4562416704 | 231 | 8.8e-60 |
| CCP20048.1 | divL1\_protein | BGC0001119 | Divergolide\_biosynthetic\_gene\_cluster | 29 | 31.0972900933 | 231 | 8.8e-60 |
| ABS90478.1 | PKS | BGC0001106 | Oxazolomycin\_biosynthetic\_gene\_cluster | 31 | 23.32296757 | 231 | 1.1e-59 |
| BAB69194.1 | modular\_polyketide\_synthase | BGC0000117 | Oligomycin\_biosynthetic\_gene\_cluster | 28 | 31.2305641937 | 230 | 1.5e-59 |
| AAP42857.1 | NanA3 | BGC0000105 | Nanchangmycin\_biosynthetic\_gene\_cluster | 34 | 22.3011994669 | 230 | 2.6e-59 |
| AAO65797.1 | monensin\_polyketide\_synthase\_module\_2 | BGC0000100 | Monensin\_biosynthetic\_gene\_cluster | 28 | 30.0310972901 | 230 | 2.6e-59 |
| AAP42856.1 | NanA2 | BGC0000105 | Nanchangmycin\_biosynthetic\_gene\_cluster | 29 | 30.2976454909 | 229 | 4.4e-59 |
| AGY62754.1 | EbeB | BGC0000051 | Ebelactone\_biosynthetic\_gene\_cluster | 29 | 33.0519768992 | 229 | 4.4e-59 |
| AHD05620.1 | putative\_polyketide\_synthase\_subunit | BGC0001033 | Paenilamicin\_biosynthetic\_gene\_cluster | 27 | 30.4309195913 | 228 | 7.4e-59 |
| ABC84457.1 | NigAII | BGC0000114 | Nigericin\_biosynthetic\_gene\_cluster | 28 | 31.985784096 | 228 | 9.7e-59 |
| ADO69378.1 | beta-ketoacyl\_synthase-like\_protein,\_Acyl\_transferase\_subunit | BGC0000044 | Dawenol\_biosynthetic\_gene\_cluster | 28 | 33.007552199 | 228 | 9.7e-59 |
| AAM12925.2 | MmpE | BGC0000182 | Mupirocin\_biosynthetic\_gene\_cluster | 32 | 20.1243891604 | 228 | 9.7e-59 |
| ABC84460.1 | NigAV | BGC0000114 | Nigericin\_biosynthetic\_gene\_cluster | 29 | 30.0755219902 | 227 | 1.3e-58 |
| AIC32692.1 | FR9C | BGC0001113 | Spliceostatin\_/\_FR901464\_biosynthetic\_gene\_cluster | 33 | 20.4797867614 | 226 | 2.8e-58 |
| ADH01484.1 | putative\_type-I\_PKS | BGC0001096 | FR901464\_biosynthetic\_gene\_cluster | 33 | 20.4797867614 | 226 | 2.8e-58 |
| CUI25743.1 | Polyketide\_synthase | BGC0001354 | U-68204\_biosynthetic\_gene\_cluster | 32 | 22.656597068 | 226 | 3.7e-58 |
| BAF85838.1 | modular\_polyketide\_synthase | BGC0000109 | Nemadectin\_biosynthetic\_gene\_cluster | 29 | 30.6086183918 | 224 | 1.4e-57 |
| AGJ76601.1 | HglE | BGC0000869 | Heterocyst\_glycolipids\_biosynthetic\_gene\_cluster | 26 | 42.2478898267 | 223 | 2.4e-57 |
| ACY06292.1 | modular\_polyketide\_synthase | BGC0001042 | Sanglifehrin\_A\_biosynthetic\_gene\_cluster | 27 | 34.7401155042 | 221 | 9.1e-57 |
| AAM12934.1 | MmpF | BGC0000182 | Mupirocin\_biosynthetic\_gene\_cluster | 27 | 32.4300310973 | 220 | 2e-56 |
| ACY06288.1 | type\_I\_polyketide\_synthase | BGC0001042 | Sanglifehrin\_A\_biosynthetic\_gene\_cluster | 26 | 39.1381608174 | 218 | 7.7e-56 |
| AAZ94389.1 | modular\_polyketide\_synthase | BGC0000040 | Concanamycin\_A\_biosynthetic\_gene\_cluster | 28 | 30.4753442914 | 216 | 2.9e-55 |
| AEH59110.1 | polyketide\_synthase\_of\_type\_I | BGC0000385 | Lysobactin\_biosynthetic\_gene\_cluster | 30 | 24.3003109729 | 216 | 3.8e-55 |
| ACY06286.1 | polyketide\_synthase | BGC0001042 | Sanglifehrin\_A\_biosynthetic\_gene\_cluster | 28 | 30.1643713905 | 213 | 2.5e-54 |
| CAQ52623.1 | type\_I\_polyketide\_synthase,\_module\_6 | BGC0001066 | Kendomycin\_biosynthetic\_gene\_cluster | 28 | 30.342070191 | 213 | 3.2e-54 |
| AIC32695.1 | FR9I | BGC0001113 | Spliceostatin\_/\_FR901464\_biosynthetic\_gene\_cluster | 31 | 21.9013771657 | 211 | 9.4e-54 |
| ADH01490.1 | type\_I\_polyketide\_synthase | BGC0000995 | FR901464\_biosynthetic\_gene\_cluster | 31 | 21.9013771657 | 211 | 9.4e-54 |
| CAJ76289.1 | putative\_hybrid\_non-ribosomal\_peptide-polyketide\_synthetase | BGC0000972 | Colibactin\_biosynthetic\_gene\_cluster | 29 | 23.5006663705 | 209 | 3.6e-53 |
| CBK62718.1 |  | BGC0001115 | Thiomarinol\_biosynthetic\_gene\_cluster | 29 | 23.6339404709 | 208 | 8e-53 |
| AGN11883.1 | TstI | BGC0001114 | Thailanstatin\_biosynthetic\_gene\_cluster | 30 | 21.3682807641 | 207 | 1.4e-52 |
| BAE93722.1 | type\_I\_polyketide\_synthase | BGC0000164 | Tetronomycin\_biosynthetic\_gene\_cluster | 28 | 30.0310972901 | 207 | 1.8e-52 |
| ABP57746.1 | DepB | BGC0000993 | FK228\_biosynthetic\_gene\_cluster | 30 | 24.7445579742 | 205 | 8.8e-52 |
| CAJ88186.1 | putative\_modular\_polyketide\_synthase | BGC0000151 | Stambomycin\_biosynthetic\_gene\_cluster | 28 | 30.1643713905 | 202 | 5.7e-51 |
| ABC38737.1 | polyketide\_synthase | BGC0000964 | Burkholdac\_biosynthetic\_gene\_cluster | 30 | 23.5006663705 | 196 | 4.1e-49 |
| AAK57185.1 | MxaB1 | BGC0001022 | Myxalamid\_biosynthetic\_gene\_cluster | 27 | 29.9422478898 | 190 | 2.9e-47 |
| CCE88377.1 | non-ribosomal\_peptide\_synthetase/polyketide\_synthase | BGC0001034 | Pellasoren\_biosynthetic\_gene\_cluster | 28 | 31.0528653932 | 187 | 1.5e-46 |
| ALD82524.1 | polyketide\_synthase | BGC0001212 | Nannocystin\_biosynthetic\_gene\_cluster | 26 | 32.4744557974 | 187 | 1.5e-46 |
| CBK62729.1 |  | BGC0001115 | Thiomarinol\_biosynthetic\_gene\_cluster | 28 | 20.6130608618 | 182 | 6.1e-45 |
| CAJ76285.1 | putative\_polyketide\_synthase | BGC0000972 | Colibactin\_biosynthetic\_gene\_cluster | 26 | 26.4326965793 | 173 | 2.8e-42 |
